# PSMA-Oriented Target Delivery of Novel Anticancer Prodrugs: Design, Synthesis, and Biological Evaluations of Oligopeptide-Camptothecin Conjugates

**DOI:** 10.3390/ijms19103251

**Published:** 2018-10-19

**Authors:** Bing Xu, Fei Zhou, Meng-Meng Yan, De-Sheng Cai, Wen-Bo Guo, Yu-Qin Yang, Xiao-Hui Jia, Wen-Xi Zhang, Tong Li, Tao Ma, Peng-Long Wang, Hai-Min Lei

**Affiliations:** School of Chinese Pharmacy, Beijing University of Chinese Medicine, Beijing 100102, China; weichenxubing@126.com (B.X.); zf116318@163.com (F.Z.); yanmengmeng@bucm.edu.cn (M.-M.Y.); cai1225366978@163.com (D.-S.C.); wb_guo@126.com (W.-B.G.); yangyq5@163.com (Y.-Q.Y.); 18811508665@163.com (X.-H.J.); zhangwxnn@126.com (W.-X.Z.); lt1755258545@163.com (T.L.)

**Keywords:** camptothecin, oligopeptide derivatives, prostate-specific membrane antigen, structure-activity relationships, apoptosis

## Abstract

Clinical applications of camptothecin (**CPT**) have been heavily hindered due to its non-targeted toxicity, active lactone ring instability, and poor water solubility. Targeted drug delivery systems may offer the possibility to overcome the above issues as reported. In this research, a series of prostate-specific membrane antigen (PSMA)-activated **CPT** prodrugs were designed and synthesized by coupling water-soluble pentapeptide, a PSMA hydrolyzing substrate, to **CPT** through an appropriate linker. The cytotoxicity of **CPT** prodrugs was masked temporarily until they were hydrolyzed by the PSMA present within the tumor sites, which restored cytotoxicity. The in vitro selective cytotoxic activities of the prodrugs were evaluated against PSMA-expressing human prostate cancer cells LNCaP-FGC and non-PSMA-expressing cancer cells HepG2, Hela, MCF-7, DU145, PC-3 and normal cells MDCK, LO2 by standard methylthiazol tetrazolium (MTT) assay. Most of the newly synthesized **CPT** prodrugs showed excellent selective toxicity to PSMA-producing prostate cancer cells LNCaP-FGC with improved water solubility. From among the library, **CPT-HT-J-ZL_12_** showed the best cytotoxic selectivity between the PSMA-expressing and the non-PSMA-expressing cancer cells. For example, the cytotoxicity of **CPT-HT-J-ZL_12_** (IC_50_ = 1.00 ± 0.20 µM) against LNCaP-FGC (PSMA^+^) was 40-fold, 40-fold, 21-fold, 5-fold and 40-fold, respectively, higher than that against the non-PSMA-expressing cells HepG2 (IC_50_ > 40.00 µM), Hela (IC_50_ > 40.00 µM), MCF-7 (IC_50_ = 21.68 ± 4.96 µM), DU145 (IC_50_ = 5.40 ± 1.22 µM), PC-3 (IC_50_ = 42.96 ± 3.69 µM) cells. Moreover, **CPT-HT-J-ZL_12_** exhibited low cytotoxicity (IC_50_ > 40 μM) towards MDCK and LO2 cells. The cellular uptake experiment demonstrated the superior PSMA-targeting ability of the **CPT-HT-J-ZL_12_**, which was significantly accumulated in LNCaP-FGC (PSMA^+^), while it was minimized in HepG2 (PSMA^−^) cells. Further cell apoptosis analyses indicated that it showed a dramatically higher apoptosis-inducing activity in LNCaP-FGC (PSMA^+^) cells than in HepG2 (PSMA^−^) cells. Cell cycle analysis indicated that **CPT-HT-J-ZL_12_** could induce cell cycle arrest at the S phase.

## 1. Introduction

Cancer remains one of the major diseases that severely threatens human life and health. Chemotherapy is still one of the main clinical treatments for cancer, either alone or in combination with other treatment options [1,2]. The low selectivity of conventional chemotherapeutic drugs induces serious adverse events and systemic toxicities [3,4,5,6]. Therefore, it is of great importance to develop targeted anti-cancer drug delivery.

Camptothecin (**CPT**), a natural alkaloid from *Camptotheca acuminate*, possesses remarkable antitumor properties against a variety of cancers via inhibition of DNA enzyme topoisomerase I (topo I) [7,8,9]. Even though **CPT** exhibits potent anticancer activities in vitro and vivo, its therapeutic application is severely hindered due to its poor aqueous solubility and high lipophilicity, active lactone ring instability, and non-targeted toxicity [10,11,12]. Previous research has shown that structural modifications at the 20-OH of camptothecin could enhance the stability of the lactone ring, and prodrugs derivatized at this site have been vigorously pursued [13,14,15,16].

The prostate-specific membrane antigen (PSMA) is a 750-amino acid type II transmembrane peptidase enzyme that is encoded by the folate hydrolase 1 (*FOLH1*) gene. Previous studies demonstrated that PSMA was highly expressed in prostate cancer cells and in tumor endothelial cells (ECs) of a variety of non-prostatic solid tumor types but was not expressed by ECs in normal tissues. In addition, PSMA is also known as glutamate carboxypeptidase II, *N*-acetyl-l-aspartyl-l-glutamate peptidase I, and *N*-acetylaspartylglutamate peptidase. It contains a binuclear zinc site and is active as a glutamate carboxypeptidase, catalyzing the hydrolytic cleavage of α- or γ-linked glutamates from peptides or small molecules [17,18,19,20]. Based on the above, PSMA is becoming one of the current research hotspots and is an excellent target for the anti-angiogenesis targeted therapy [19,20,21,22,23]. The enzymatic activity of PSMA can be exploited for the design of prodrugs, in which an inactive form of the cytotoxic drug is selectively cleaved and thereby activated only at cells that express PSMA within the tumor microenvironment.

In view of the unique carboxypeptidase activity and restricted expression of the PSMA, we accomplished the synthesis of several PSMA-activated camptothecin pentapeptide prodrugs **CPT-HT-J-ZLn**, by introducing an oriented oligopeptide **HT-J** (Glu*Glu*Glu*Asp-Glu) (“*” mean the γ-glutamyl linkage; “-” mean the α-glutamyl linkage) to the 20-OH of **CPT** via carbonate linkers with different lengths, in order to improve their tumor targeting, stability, and aqueous solubility. All newly synthesized compounds were fully characterized by ^1^H-NMR, ^13^C-NMR, and HRMS; they were also tested for selective cytotoxic activity against PSMA-expressing human prostate cancer cell LNCaP-FGC and non-PSMA-expressing cells HepG2, Hela, MCF-7, DU145, and PC-3, respectively [24,25,26,27,28] and normal cells MDCK, LO2 by standard MTT assay. Meanwhile, the preliminary anti-tumor mechanisms of the most selectivity compound **CPT-HT-J-ZLn** were also investigated by fluorescence staining observation and flow cytometric analysis in the present study. In addition, the structure-activity relationships of these derivatives are briefly discussed.

## 2. Results

### 2.1. Chemistry

**Scheme 1:**
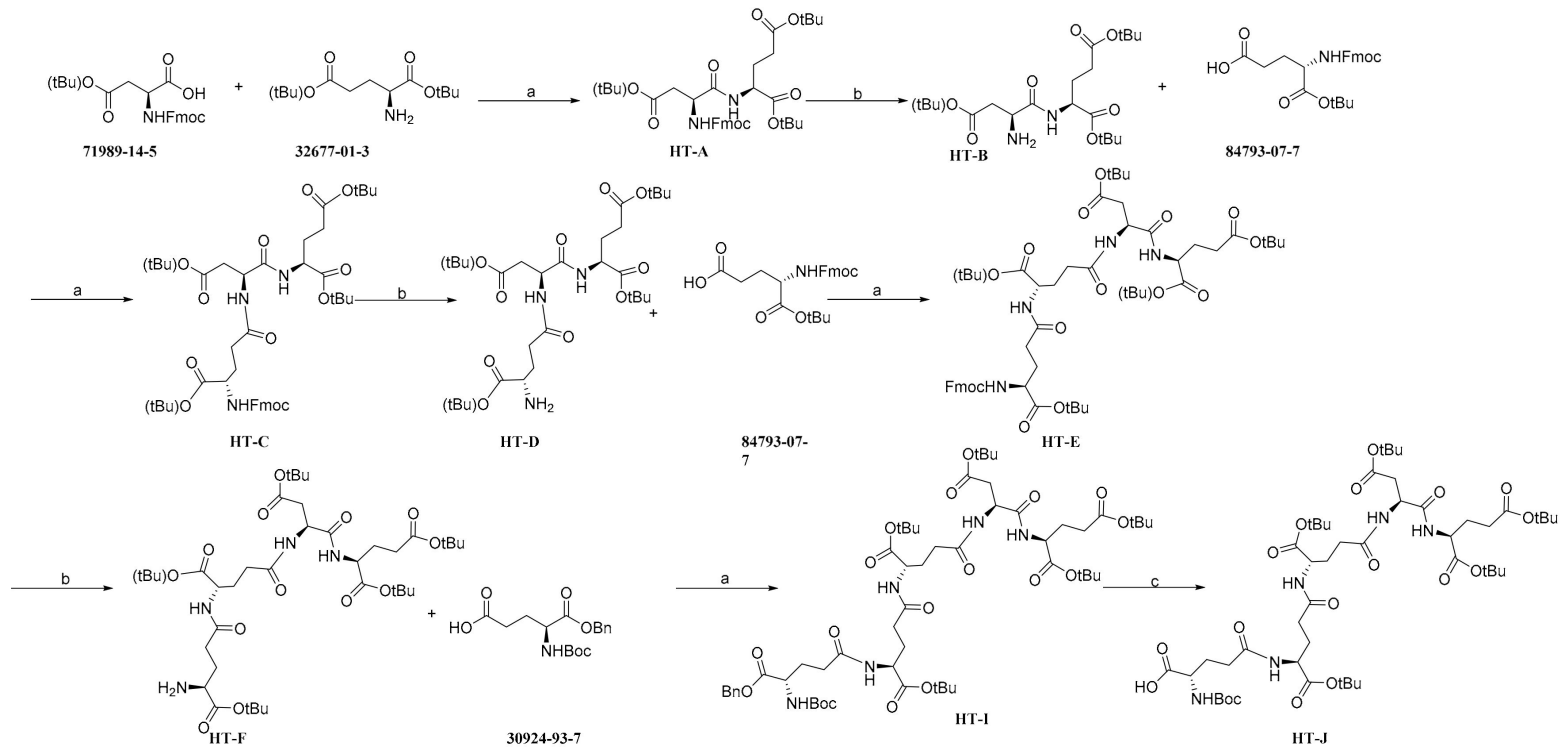
Synthesis of the pentapeptide **HT-J**. Reagents and Conditions: (**a**) EDCI, HOBt, DIPEA, DCM, 25 °C, 3 h; (**b**) Piperidine, dimethylformamide (DMF), 1 h; (**c**) Wet Pd/C (5%), MeOH, 12 h.

**Scheme 2:**
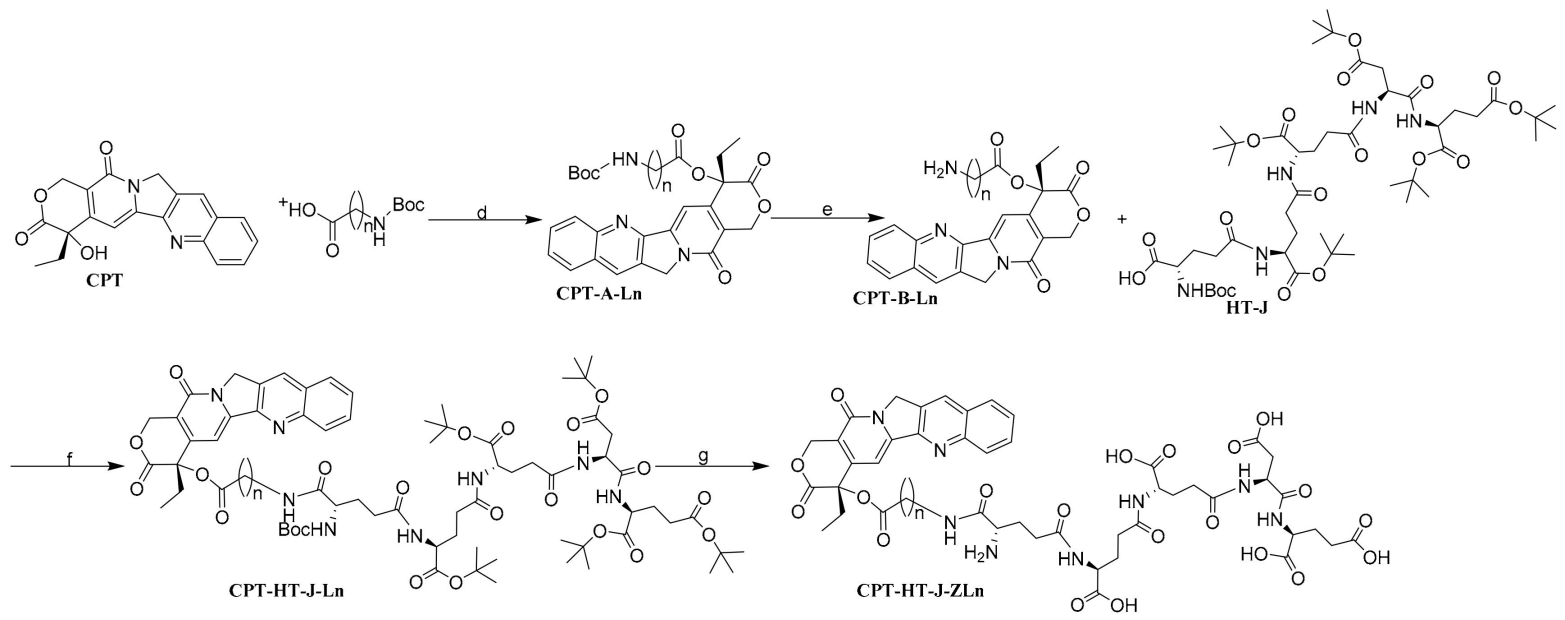
Synthesis of the PSMA-activated prodrugs **CPT-HT-J-ZLn**. Reagents and Conditions: (**d**) EDCI, DMAP, DCM, 25 °C, 12 h; (**e**) TFA, DCM, 25 °C, 2 h; (**f**) EDCI, HOBt, DIPEA, DCM, 25 °C, 3 h; (**g**) Trifluoroacetic (TFA):Thioanisole:H_2_O = 95:2.5:2.5, 25 °C, 3 h.

**Scheme 3:**
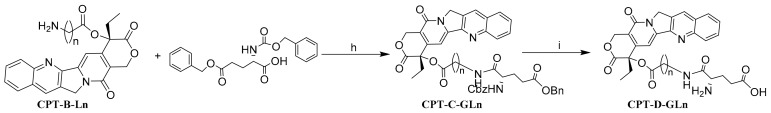
Synthesis of the prostate-specific membrane antigen (PSMA) hydrolysates **CPT-D-GLn**. Reagents and Conditions: (**h**) EDCI, HOBt, DIPEA, DCM, 25 °C, 3 h; (**i**) Wet Pd/C (5%), MeOH, 12 h.

The designed derivatives were prepared following the procedures in Scheme 1, Scheme 2 and Scheme 3. **HT-J** (Glu*Glu*Glu*Asp-Glu), the pentapeptide used to target PSMA, was synthesized by liquid-phase peptide chemistry starting with Fmoc-Asp(OtBu)-OH (Scheme 1).

The intermediate **CPT-A-Ln** was subsequently prepared by 1-(3-dimethylaminopropyl)-3-ethylcarbodiimide hydrochloride (EDCI)-mediated esterification from **CPT**. Then **CPT-A-Ln** was further treated with trifluoroacetic (TFA) in dry dichloromethane (DCM) to remove the Boc-protective group, and the key intermediate **CPT-B-Ln** was successfully obtained (Scheme 2).

For the synthesis of the PSMA-activated prodrug **CPT-HT-J-ZLn**, reaction of **CPT-B-Ln** with **HT-J** in the presence of EDCI, *N*,*N*-Diisopropylethylamine (DIPEA), and 1-hydroxybenzotriazole (HOBT) in DCM afforded the corresponding intermediate **CPT-HT-J-Ln**, which was further treated with TFA to remove the protective groups, yielding the final prodrug **CPT-HT-J-ZLn** (Scheme 2). It is worth noting that the tert-butyl cation released in the decomposition of the tert-butyl ester was a powerful electrophile that might react with the substrate. To avoid the formation of undesirable by-products, the nucleophilic scavenger thioanisole was added to TFA.

Previous studies have shown that PSMA is an exopeptidase, and hydrolytic processing of any prodrug would result in a product consisting of a cytotoxin coupled to the acidic amino acids glutamate or aspartate [19]. To better study the PSMA-targeting ability of the prodrug **CPT-HT-J-ZL_12_**, the PSMA hydrolysate **CPT-D-Ln** was prepared as well, according to the procedures in Scheme 3 in this study. In brief, the intermediate **CPT-B-Ln** underwent coupling reaction with *N*-Cbz-l-glutamic acid 5-benzyl ester in the presence of EDCI, HOBt, and DIPEA in dry DCM to afford the intermediate **CPT-C-GLn**, which was further treated with Pd/C (10%) in methanol (MeOH) to give the final compound **CPT-D-GLn**. The structures of all target derivatives were confirmed by spectral (^1^H-NMR, ^13^C-NMR, and HRMS) analysis (Figures S1–S122).

### 2.2. Cytotoxicity

**Figure 1:**
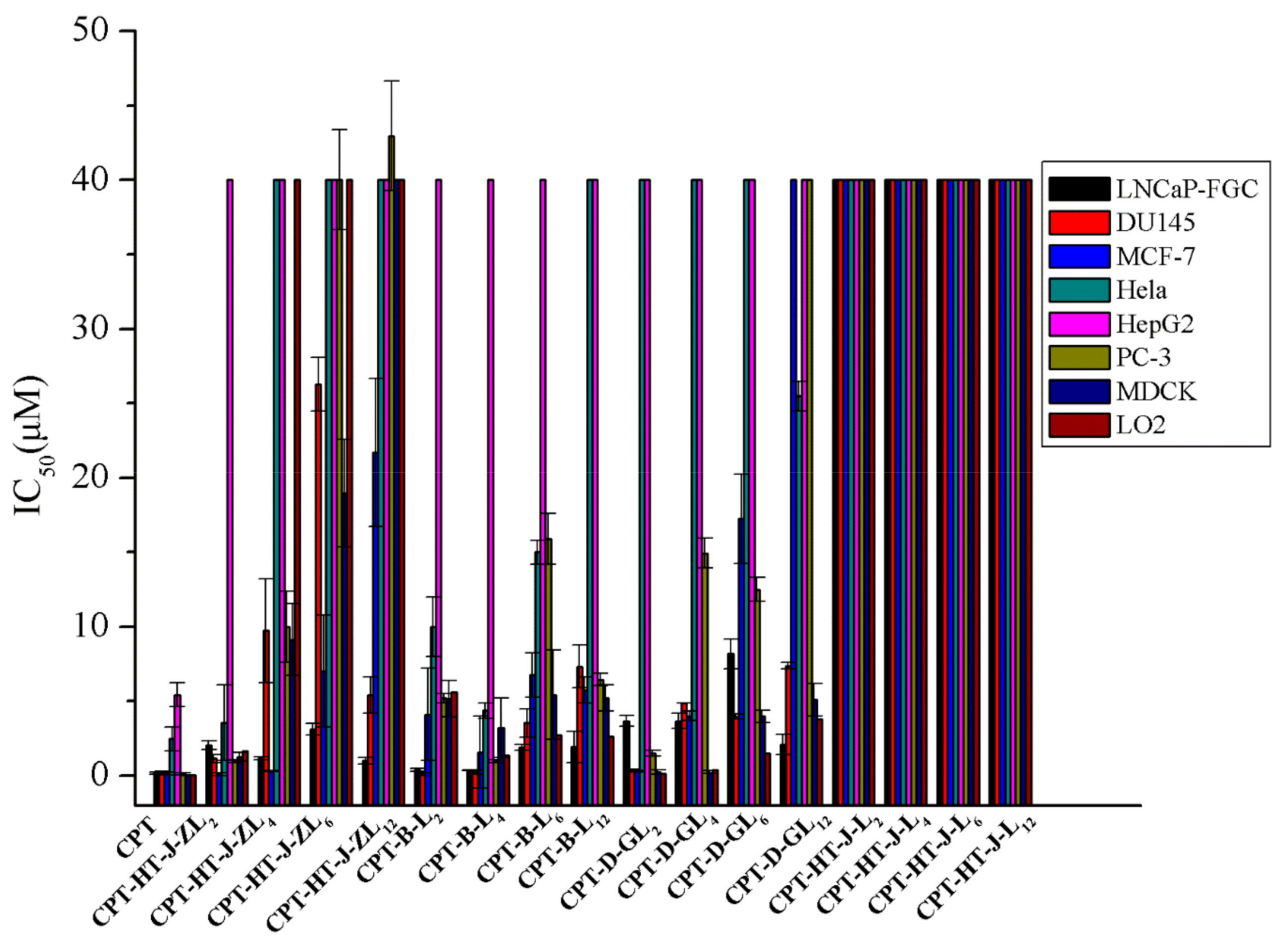
The cytotoxicities of the **CPT-HT-J-ZLn** and the PSMA hydrolysate **CPT-D-GLn** to different tumor and normal cells.

The cytotoxicities of PSMA-activated prodrug **CPT-HT-J-ZLn** and PSMA hydrolysate **CPT-D-GLn** were evaluated by MTT assays on PSMA-expressing (PSMA^+^) cancer cell LNCaP-FGC, non-PSMA-expressing (PSMA^−^) cancer cells HepG2, MCF-7, Hela, DU145, and PC-3, and normal cells MDCK, LO2, respectively, using **CPT** as a positive control. The IC_50_ values are summarized in Table 1 and Figure 1.

As shown in Table 1 and Figure 1, most of the PSMA-activated prodrug **CPT-HT-J-ZLn** showed moderate to potent cytotoxicity against all the tested tumor cell lines. However, their cytotoxicity was much lower than the parent compound **CPT**. The cytotoxicity detection also revealed that most of the prodrug **CPT-HT-J-ZLn** exhibited better anti-proliferative activities against the PSMA-expressing cell line LNCaP-FGC than the non-PSMA-expressing cell lines HepG2, Hela, MCF-7, DU145, and PC-3.

**Figure 2:**
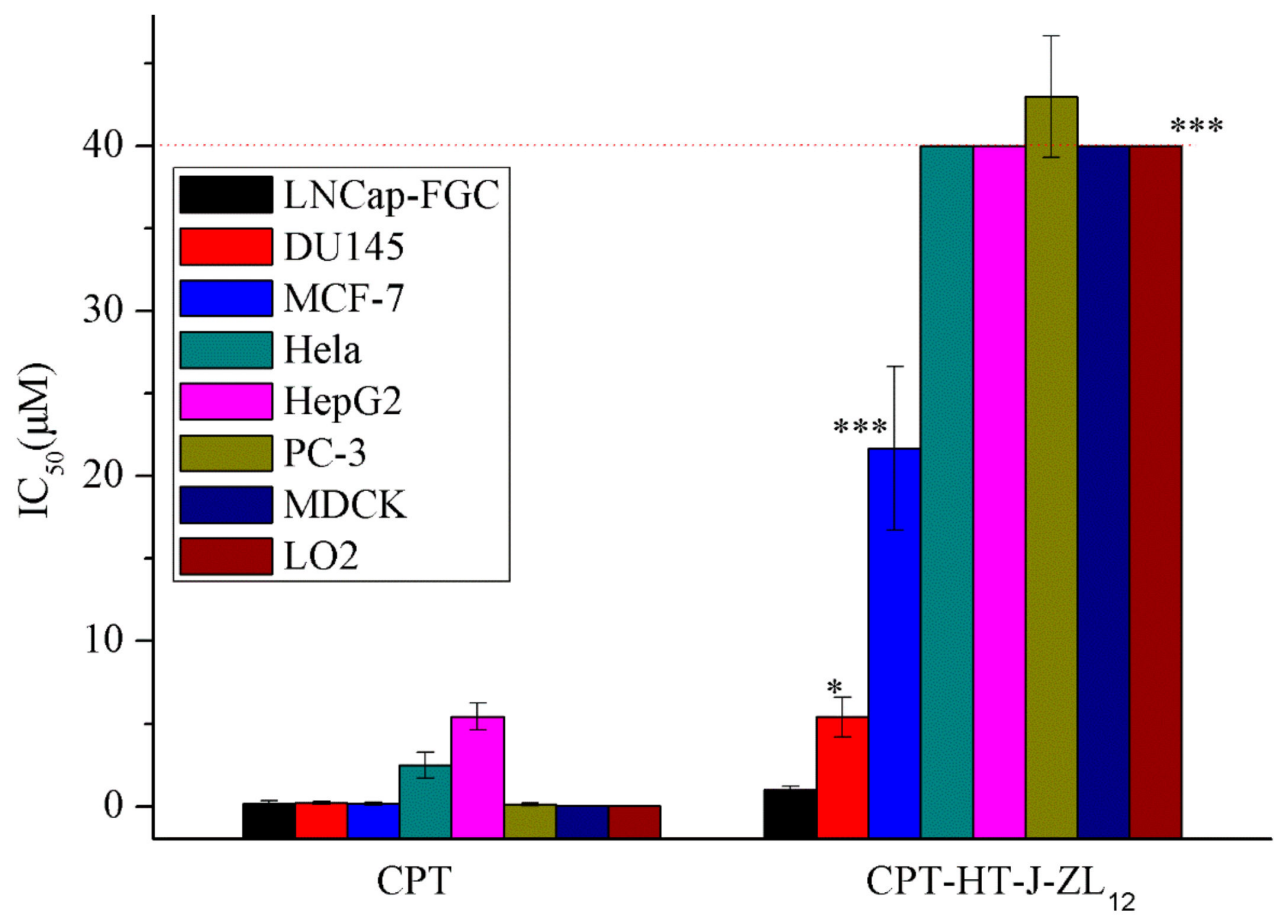
The IC_50_ values of **CPT** and the prodrug **CPT-HT-J-ZL_12_** on different cell lines. * *p* < 0.05, *** *p* < 0.001, vs. LNCaP-FGC cells.

From among the library, **CPT-HT-J-ZL_12_** showed the most cytotoxic selectivity against the PSMA-expressing and the non-PSMA-expressing cancer cells. For example, the cytotoxicity of **CPT-HT-J-ZL_12_** (IC_50_ = 1.00 ± 0.20 µM) against LNCaP-FGC (PSMA^+^) was 40-fold, 40-fold, 21-fold, 5-fold and 40-fold, respectively, higher than that against the non-PSMA-expressing cells HepG2 (IC_50_ > 40.00 µM), Hela (IC_50_ > 40.00 µM), MCF-7 (IC_50_ = 21.68 ± 4.96 µM), DU145 (IC_50_ = 5.40 ± 1.22 µM), PC-3 (IC_50_ = 42.96 ± 3.69 µM) cells. Moreover, **CPT-HT-J-ZL_12_** exhibited low cytotoxicity (IC_50_ > 40.00 µM) towards MDCK and LO2 cells (Figure 2). This was probably because LNCaP-FGC had expression of PSMA, while the others did not. The PSMA present at the membrane of the tumor cells was able to hydrolyze the pentapeptide, and then the subsequent active compound was able to enter the cells and exert the cytotoxic effects. Interestingly, among the non-PSMA-expressing cells, the prodrug **CPT-HT-J-ZLn**, showed much higher cytotoxicities against MCF-7, DU145, and PC-3 cell lines than other cell lines (Hela and HepG2).

Furthermore, it was observed that the length of the linker chain between **CPT** and the pentapeptide **HT-J** affected the cytotoxicity observably. With the extension of the linker chain, the cytotoxicity decreased, as exemplified by **CPT-HT-J-ZL_2_**, **CPT-HT-J-ZL_4_** > **CPT-HT-J-ZL_6_**, **CPT-HT-J-ZL_12_**. The cytotoxicity detection also revealed that most of the PSMA hydrolysate **CPT-D-GLn** and the intermediate **CPT-B-Ln** exhibited antiproliferative activities against all tested cancer cells, but their cytotoxic selectivity against the PSMA-expressing and the non-PSMA-expressing cancer cells was lower than the prodrugs **CPT-HT-J-ZLn**. Structure-activity relationship analysis among **CPT-D-GLn** and **CPT-B-Ln** also revealed that the length of the linker chain affected the cytotoxicity observably. With the extension of the linker chain, cytotoxicity was also decreased, as exemplified by **CPT-B-L_2_**, **CPT-B-L_4_** > **CPT-B-L_6_**, **CPT-B-L_12_**; **CPT-D-GL_2_**, **CPT-D-GL_4_** > **CPT-D-GL_6_**, **CPT-D-GL_12_**; therefore, the most selective compound **CPT-HT-J-ZL_12_** was picked out for further studies to investigate its mechanism of growth inhibition on HepG2 and LNCaP-FGC cell lines.

### 2.3. Computer Simulation of ClogP and the Aqueous Solubility Study

LogP, the logarithm of the octanol/water partition coefficient, is often used as the common quantitative descriptor of lipophilicity [29]. Optimization of solubility and hydrophobicity played an important role in the drug discovery process on account of their being closely associated with the absorption, distribution, metabolism, and excretion (ADME) properties of the compounds [30,31,32]. In this work, the logP values for these derivatives were calculated using the Sybyl-X 2.0 software (Tripos, Certara Inc., St. Louis, MO, USA). As shown in Table 1, all the prodrug **CPT-HT-J-ZLn** showed lower ClogP than **CPT**. Meanwhile the aqueous solubility of the prodrug **CPT-HT-J-ZLn** (19.00 mg/mL in PBS, pH = 7.4), which was much higher than **CPT** (0.003 mg/mL in PBS, pH = 7.4).

### 2.4. In Vitro Cellular Uptake

**Figure 3:**
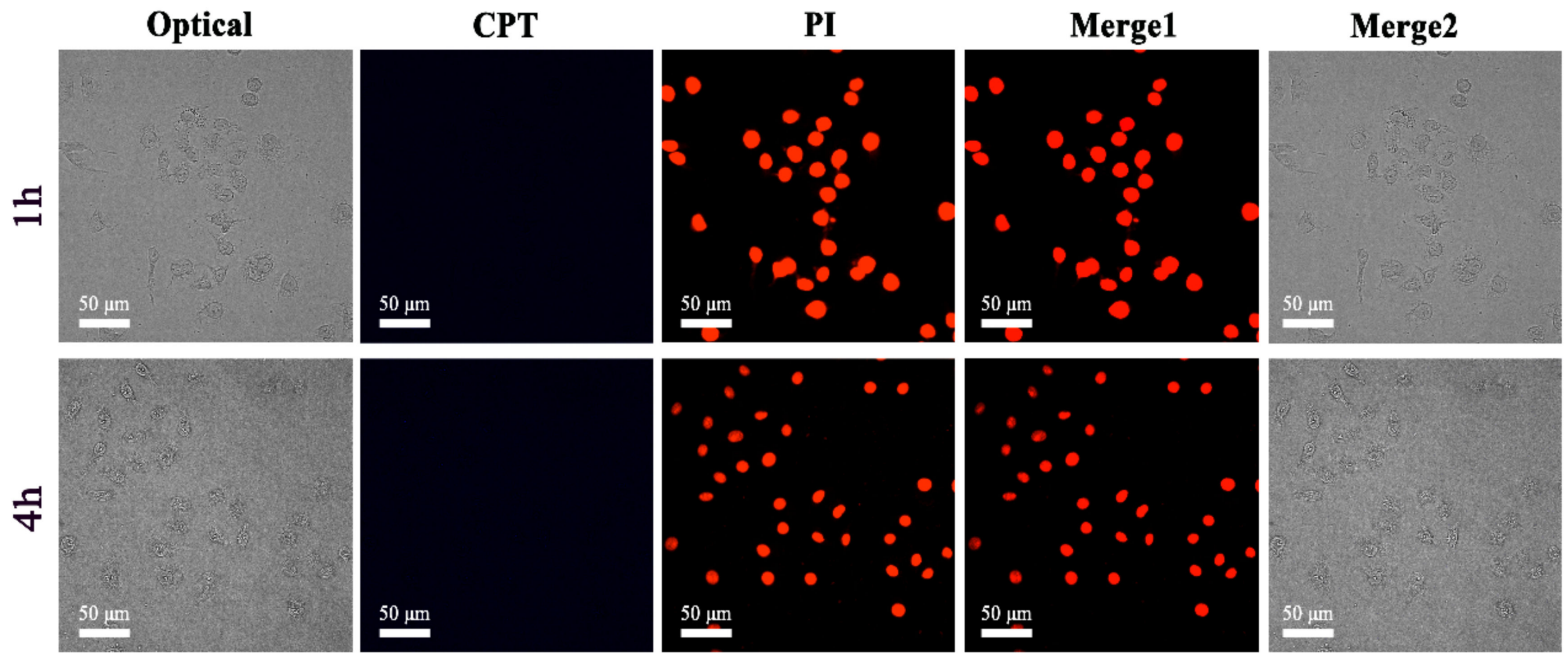
Cellular uptake of **CPT-HT-J-ZL_12_** in HepG2 (no expression of PSMA) cells. CLSM images of HepG2 cells incubated with **CPT-HT-J-ZL_12_** (10 μM) for 1 and 4 h. Nuclei were stained by PI (red), the blue color was indicative of **CPT**. **CPT**: false-color green. For **CPT**: λex = 405 nm, band-pass filter λ = 500–550 nm. For PI: λex = 561 nm, band-pass filter λ = 575–625 nm.

**Figure 4:**
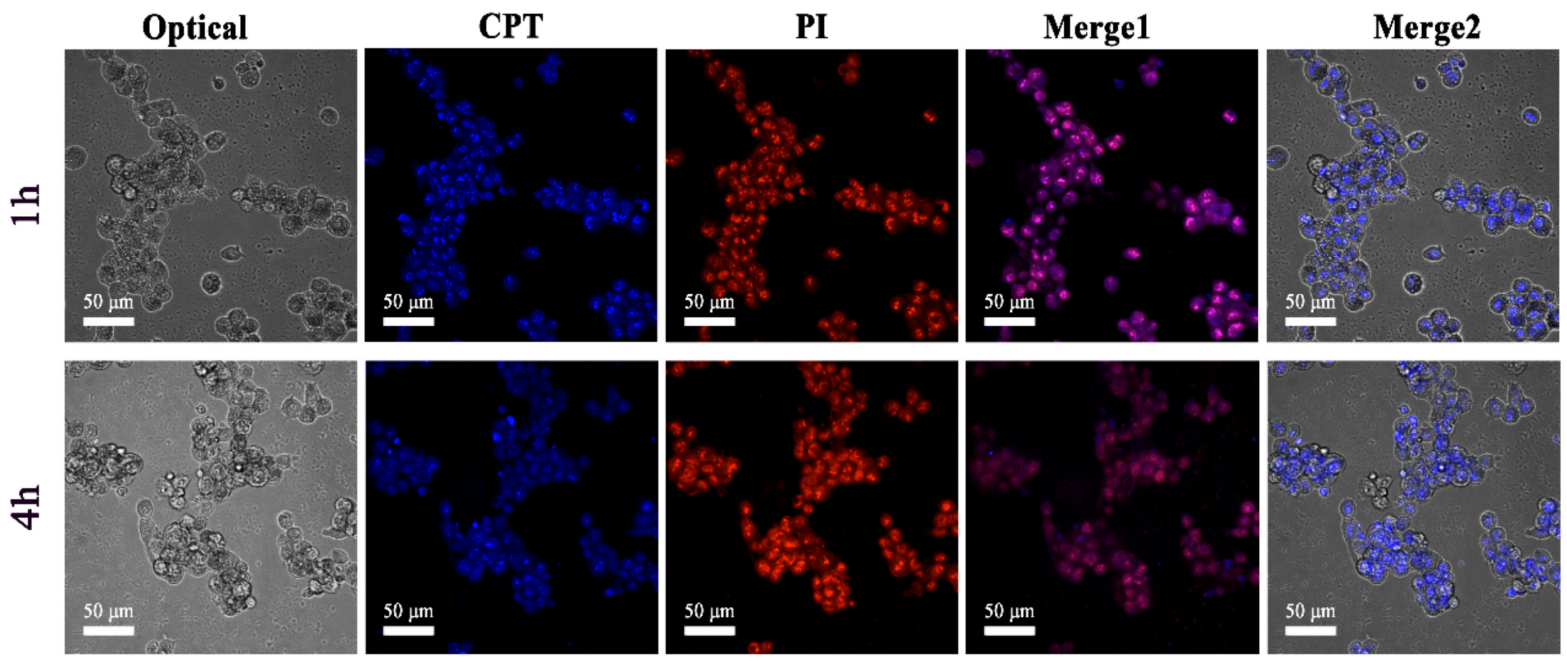
Cellular uptake of **CPT-HT-J-ZL_12_** in LNCaP-FGC (high expression of PSMA) cells. CLSM images of LNCaP-FGC cells incubated with **CPT-HT-J-ZL_12_** (10 µM) for 1 and 4 h. Nuclei were stained by PI (red); the blue color was indicative of **CPT**. **CPT**: false-color green. For **CPT**: λex = 405 nm, band-pass filter λ = 500–550 nm. For PI: λex = 561 nm, band-pass filter λ = 575–625 nm.

**Figure 5:**
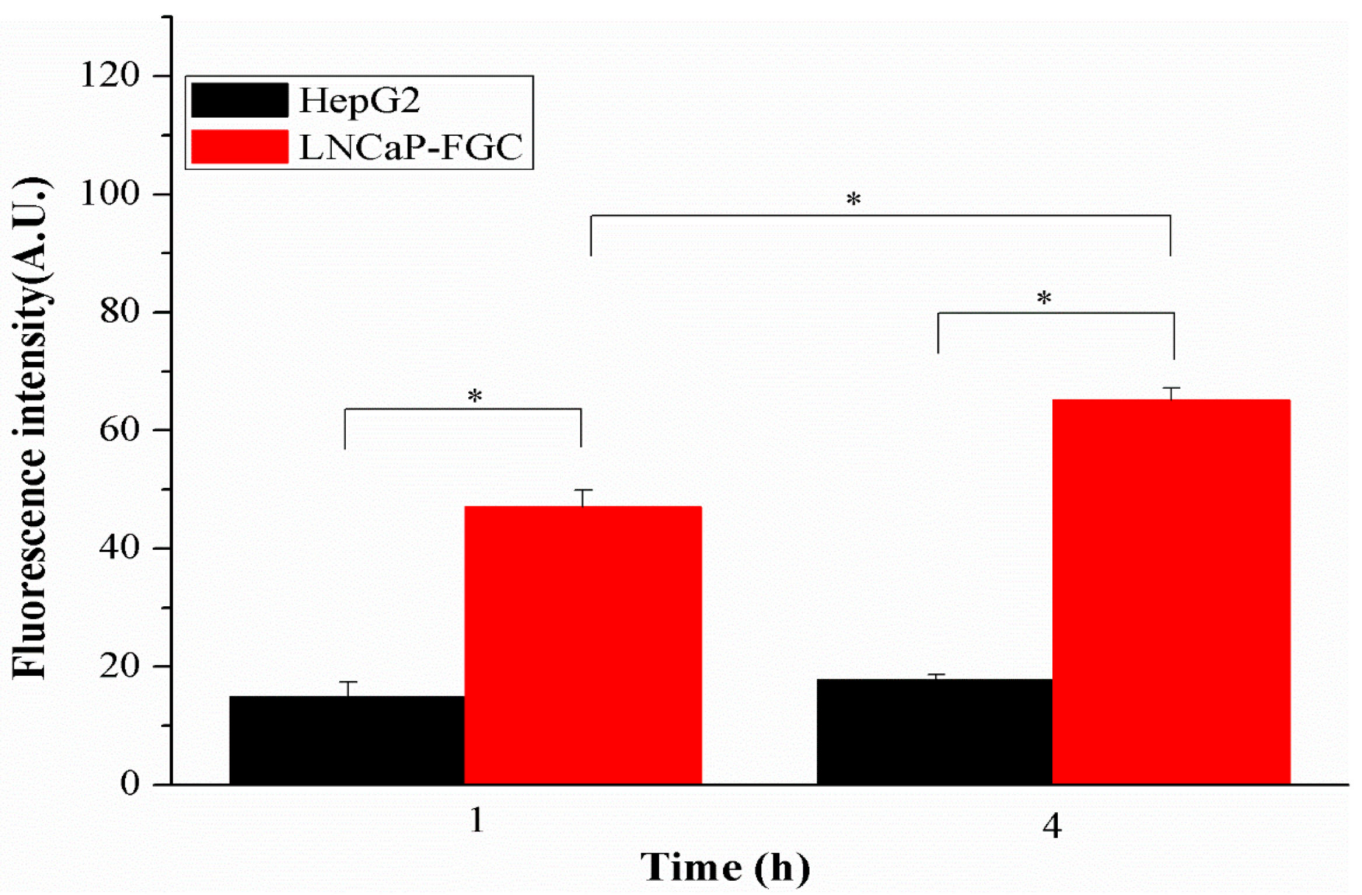
Mean fluorescence intensity of **CPT-HT-J-ZL_12_** internalized by LNCaP-FGC (high expression of PSMA) and HepG2 (none expression of PSMA) cells after incubation for 1 and 4 h. * *p* < 0.05.

In addition to solubilization, the pentapeptide serves to mask the cytotoxicity by preventing nonspecific uptake of drug into cells due to the presence of negative charged amino acids. Upon cleavage of the prodrug by PSMA within the PSMA-expressing cells, the active compound was able to enter cells. To prove our assumption, the cellular uptake behavior of **CPT-HT-J-ZL_12_** in LNCAP-FGC (PSMA^+^) and HepG2 (PSMA^−^) was investigated by confocal laser scanning microscope (CLSM). The nucleus was stained with PI (red) and the fluorescence of **CPT** was pseudo-colored with blue. As shown in Figure 3, there was almost no blue fluorescence when treated with **CPT-HT-J-ZL_12_** in HepG2 (PSMA^−^) cells for 1 h and 4 h, demonstrating the poor uptake of **CPT-HT-J-ZL_12_**. On the contrary, the blue fluorescence intensity in LNCAP-FGC (PSMA^+^) for 1 h was stronger, indicating that the active compound was able to enter the cancer cells. In addition, the blue fluorescence in LNCAP-FGC (PSMA^+^) increased with the incubation time (4 h) (Figure 4 and Figure 5), implying that prolonged treatment facilitated cellular uptake of the subsequent active compound.

### 2.5. Detection of Apoptosis Using Annexin V-FITC/PI Staining

**Figure 6:**
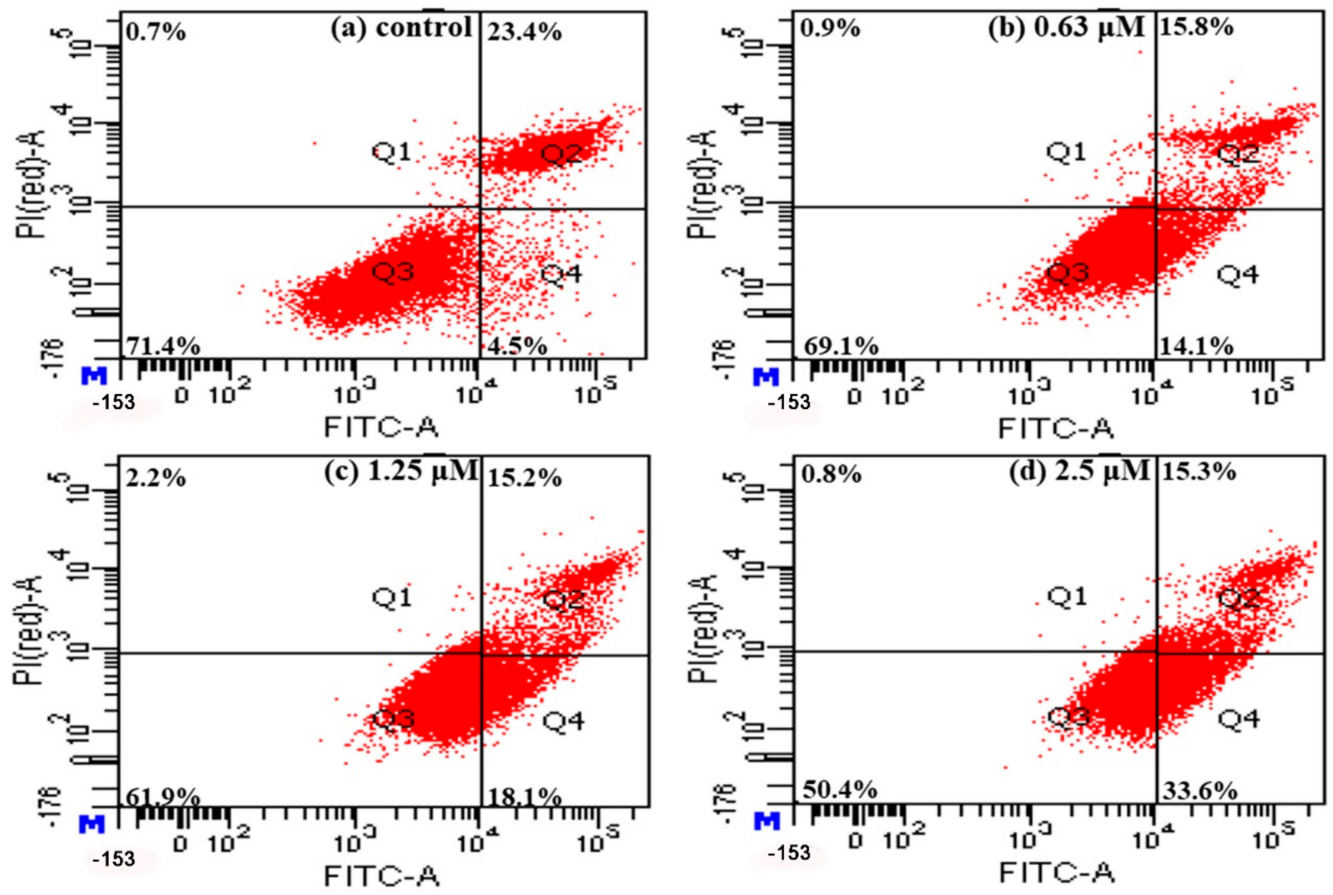
Flow cytometry analysis for apoptosis of LNCaP-FGC cells (high expression of PSMA) induced by **CPT-HT-J-ZL_12_**: (**a**) control group; (**b**) 0.63 µM; (**c**) 1.25 µM; and (**d**) 2.5 µM.

**Figure 7:**
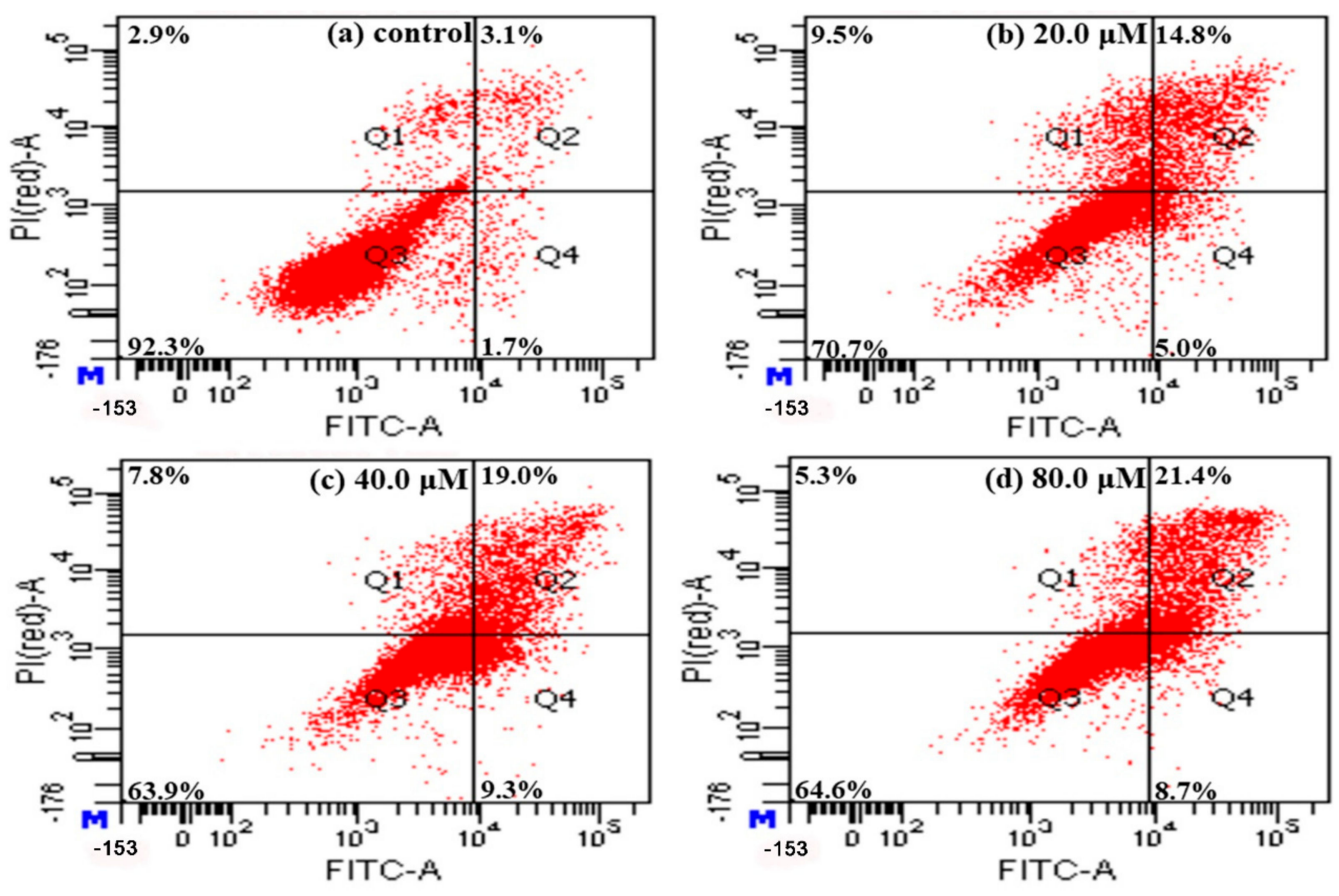
Flow cytometry analysis for apoptosis of HepG2 (none expression of PSMA) cells induced by **CPT-HT-J-ZL_12_**: (**a**) control group; (**b**) 20.0 µM; (**c**) 40.0 µM; and (**d**) 80.0 µM.

To further evaluate the selective cytotoxicity on LNCaP-FGC (PSMA^+^) and HepG2 (PSMA^−^) after treatment with **CPT-HT-J-ZL_12_**, apoptotic rates were analyzed by flow cytometry using an Annexin V-FITC/PI staining. It was observed that the LNCaP-FGC (PSMA^+^) cell demonstrated a remarkable response to the apoptotic effect of **CPT-HT-J-ZL_12_** in a dose-dependent manner. The apoptotic effect was determined by counting the apoptosis ratios (including the early and late apoptosis ratios). Following different concentrations (0.63, 1.25, 2.50 µM) of **CPT-HT-J-ZL_12_** treatment, the apoptosis ratios increased from 29.90% to 33.30% and 48.90%, respectively (Figure 6). On the contrary, the drug-induced apoptosis for HepG2 (PSMA^−^) cell demonstrated a totally different trend. The percentage of apoptotic HepG2 (PSMA^−^) cell was evaluated to be 19.80%, 28.30%, and 30.10% after treatment with 20.00 µM, 40.00 µM and 80.00 µM of **CPT-HT-J-ZL_12_**, respectively (Figure 7). These results demonstrated **CPT-HT-J-ZL_12_** showed PSMA-oriented targeting activity.

### 2.6. Detection of Cell Cycle Using PI Staining

**Figure 8:**
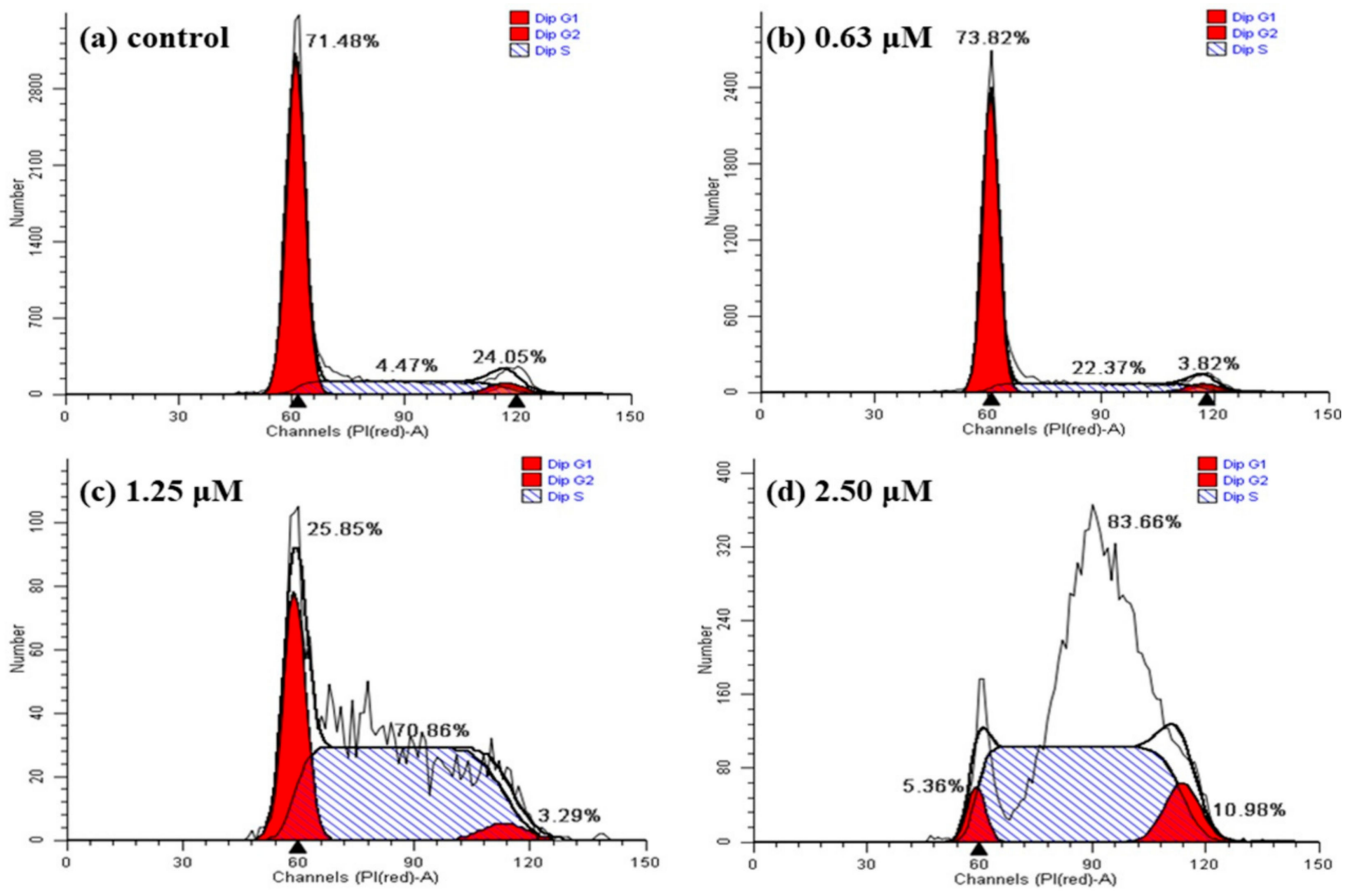
Effect of **CPT-HT-J-ZL_12_** on cell cycle progression in LNCaP-FGC cells (high expression of PSMA): (**a**) control group; (**b**) 0.63 µM; (**c**) 1.25 µM; and (**d**) 2.5 µM.

To further study **CPT-HT-J-ZL_12_**-induced growth inhibition, the cell cycle changes were evaluated on LNCaP-FGC (PSMA^+^) using flow cytometric methods. As shown in Figure 8, with the concentration of **CPT-HT-J-ZL_12_** increased from 0.63 to 1.25 to 2.50 µM, the percentage of LNCaP-FGC cells in S phase increased dramatically (from 4.47% to 70.86% to 83.66%), while the cells in G0/G1 phases decreased. This indicated that **CPT-HT-J-ZL_12_** was able to induce cell cycle arrest at the S phase.

## 3. Discussion

In this article, four novel water-soluble PSMA-activated **CPT** prodrugs were successfully achieved by coupling pentapeptide **HT-J**, a PSMA hydrolyzing substrate, to 20-OH of **CPT**. Their selective cytotoxic activities were determined on several PSMA-positive and PSMA-negative cancer cell and normal cell lines by the standard MTT assay. The results indicated that the prodrug **CPT-HT-J-ZLn** showed significantly selective cytotoxicity against the PSMA-positive and PSMA-negative cell lines. **CPT-HT-J-ZL_12_**, the lead PSMA-activated prodrug identified herein, was ~40-fold more toxic in vitro to PSMA-positive cell LNCaP-FGC (IC_50_ = 1.00 ± 0.20 µM) compared to PSMA-negative cells HepG2 (IC_50_ > 40.00 µM) and Hela (IC_50_ > 40.00 µM). Moreover, **CPT-HT-J-ZL_12_** exhibited low cytotoxicity (IC_50_ > 40 µM) towards MDCK and LO2 cells. The in vitro cellular uptake study and the cell apoptosis analyses demonstrated the PSMA-oriented targeting of **CPT-HT-J-ZL_12_**. The cell cycle analysis indicated that **CPT-HT-J-ZL_12_** could induce cell cycle arrest at the S phase. The PSMA present at the membrane of the PSMA-positive cells was able to hydrolyze the pentapeptide; then the subsequent active compound was able to enter cells and exert the toxic effect. The preclinical results presented here suggested that the attempt to discover tumor-targeting lead compounds by using this PSMA-activated prodrug strategy was viable. Consequently, our original design concept should be perfectly illustrated by the biological evaluations above. In addition, the compound **CPT-HT-J-ZL_12_** was selected for further pharmacodynamics and pharmacokinetic evaluation, including in vivo antitumor activity against LNCaP-FGC human prostate cancer mouse xenograft and MCF-7 human breast cancer xenograft, in vivo pharmacokinetics, in the hope of producing drug candidates for drug development.

## 4. Materials and Methods

### 4.1. Chemistry

Materials were obtained from commercial suppliers and used without further purification unless otherwise mentioned. Reactions were monitored by TLC using silica gel-coated aluminum sheets (Qingdao Haiyang Chemical Co., Qingdao, China). All melting points were determined on a micro melting point apparatus and were uncorrected. NMR spectra were recorded on a BRUKER AVANCE 500 and 400 MHz spectrometer (Fällanden, Switzerland) with tetramethylsilane (TMS) as an internal standard; chemical shifts *δ* were given in ppm and coupling constants *J* in Hz. HR-MS were acquired using a Thermo Sientific TM LTQ Orbitrap XL hybrid FTMS instrument (Thermo Technologies, New York, NY, USA).

#### 4.1.1. General Synthetic Procedure of Condensation Reaction (Method A)

The corresponding intermediate (1 equiv.) was dissolved in dry DCM (20 mL), EDCI (1.5 equiv.) and the corresponding protected amino acid (1.1 equiv.) were added. After addition of HOBT (1.5 equiv.) and DIPEA (2.5 equiv.), the mixture was stirred at 25 °C for 4 h. The reaction mixture was diluted with 50 mL CH_2_Cl_2_, then successively washed with water and brine (10 mL each), dried over sodium sulfate and filtered, and the solvent was evaporated. Then the crude product was purified by flash chromatography (silica gel, petroleum ether:ethyl acetate =10:1 to 3:1).

#### 4.1.2. General Synthetic Procedure of Deprotection (Method B)

To a solution of the Fmoc-protected compound (1 equiv.) in dry DMF (20 mL), piperidine (3 equiv.) was added. The mixture was allowed to stir at room temperature for 0.5 h. After completion of the reaction (as monitored by TLC), the solution was evaporated, and the residue was dissolved with ethyl acetate (50 mL). The organic extracts were washed with distilled water (3 × 25 mL) and brine (20 mL), dried over sodium sulfate, filtrated and evaporated. Purification was performed by flash chromatography (silica gel, petroleum ether:ethyl acetate = 3:1 to 0:1).

#### 4.1.3. General Synthetic Procedure of Deprotection (Method C)

Pd/C (10%; 80 mg) was added to a solution of the Cbz-protected compound in 30 mL MeOH. The mixture was stirred at room temperature for 12 h. After completion of the reaction (as monitored by TLC), the solvent was filtered to remove Pd/C. The filtrate was concentrated in vacuum and the residue was purified by flash chromatography (silica gel, dichloromethane:methanol = 40:1 to 10:1).

*Characterization of di-tert-butyl ((S)-2-((((9H-fluoren-9-yl)methoxy)carbonyl)amino)-4-(tert-butoxy)-4-oxobutanoyl)-l-glutamate* (**HT-A**). Obtained from Fmoc-l-aspartic acid β-tert-butyl ester by method A as a white powder; m.p. 116.6–116.9 °C; Yield: 85.8%; ^1^H-NMR (500 MHz, CDCl_3_): *δ* 1.44 (m, 27 H, 9 × -CH_3_), 1.90 (m, 1H), 2.16 (m, 1H), 2.31 (m, 2H), 2.60 (m, 1H), 2.99 (m, 1 H), 4.24 (t, *J* = 7.1 Hz, 1H), 4.42 (m, 2H), 4.45 (m, 1H), 4.56 (m, 1H), 5.99 (d, *J* = 8.5 Hz, 1H), 7.14 (d, *J* = 7.6 Hz, 1H), 7.32 (t, *J* = 7.5 Hz, 2H), 7.40 (t, *J* = 7.5 Hz, 2H), 7.60 (m, 2H), 7.76 (d, *J* = 7.6 Hz, 2H). ^13^C-NMR (126 MHz, CDCl_3_): *δ* 27.8, 28.1, 28.2, 31.4, 37.7, 47.2, 51.2, 52.6, 67.4, 80.7, 82.0, 82.4, 120.1, 125.2, 127.2, 127.9, 141.4, 144.0, 156.1, 170.4, 170.6, 171.5, 172.3; HRMS (ESI) *m*/*z*: [M + H]^+^ 653.3429, calcd. for C_36_H_48_N_2_O_9_ 652.33598.

*Characterization of di-tert-butyl ((S)-2-amino-4-(tert-butoxy)-4-oxobutanoyl)-l-glutamate* (**HT-B**). Obtained from **HT-A** by method B as a white oily liquid; Yield: 87.9%; ^1^H-NMR (500 MHz, CDCl_3_): *δ* 1.43 (s, 9H, 3 × -CH_3_), 1.44 (s, 9H, 3 × -CH_3_), 1.46 (s, 9H, 3 × -CH_3_), 1.89 (m, 2H), 2.29 (m, 2H), 2.58 (dd, *J* = 16.7, 8.0 Hz, 1H), 2.79 (dd, *J* = 16.7, 3.7 Hz, 1H), 3.66 (dd, *J* = 8.0, 3.7 Hz, 1H), 4.45 (td, *J* = 8.4, 4.9 Hz, 1H), 7.89 (d, *J* = 8.4 Hz, 1H). ^13^C-NMR (126 MHz, CDCl_3_): *δ* 28.1, 28.2, 28.3, 29.8, 31.6, 40.6, 52.1, 52.2, 80.7, 81.4, 82.3, 171.2, 171.3, 172.3, 173.5; HRMS (ESI) *m*/*z*: [M + H]^+^ 431.2751, calcd. for C_21_H_38_N_2_O_7_ 430.26790.

*Characterization of di-tert-butyl ((S)-2-((S)-4-((((9H-fluoren-9-yl)methoxy)carbonyl)amino)-5-(tert-butoxy)-5-oxopentanamido)-4-(tert-butoxy)-4-oxobutanoyl)-l-glutamate* (**HT-C**). Obtained from **HT-B** by method A as a white powder; m.p. 106.3–107.3 °C; Yield: 88.1%; ^1^H-NMR (500 MHz, CDCl_3_): *δ* 1.41 (s, 9H, 3 × -CH_3_), 1.42 (s, 9H, 3 × -CH_3_), 1.43 (s, 9H, 3 × -CH_3_), 1.47 (s, 9H, 3 × -CH_3_), 1.91 (m, 3H), 2.12 (m, 1H), 2.28 (m, 5H), 2.60 (dd, *J* = 17.1, 6.3 Hz, 1H), 2.91 (dd, *J* = 17.2, 3.8 Hz, 1H), 4.22 (dd, 1H), 4.40 (m, 3H), 4.78 (d, *J* = 4.3 Hz, 1H), 5.63 (d, *J* = 7.7 Hz, 1H), 6.94 (d, *J* = 7.4 Hz, 1H), 7.17 (d, *J* = 7.7 Hz, 1H), 7.31 (t, *J* = 7.3 Hz, 2H), 7.39 (t, *J* = 7.3 Hz, 2H), 7.61 (d, *J* = 7.3 Hz, 2H), 7.75 (d, *J* = 7.4 Hz, 2H). ^13^C-NMR (126 MHz, CDCl_3_): *δ* 27.7, 28.1, 28.2, 28.8, 31.5, 32.4, 37.2, 47.3, 49.4, 52.5, 53.9, 67.1, 80.7, 81.9, 82.3, 82.6, 120.1, 125.3, 127.2, 127.8, 141.4, 143.9, 144.0, 156.3, 170.5, 171.2, 171.6, 171.9, 172.2. HRMS (ESI) *m*/*z*: [M + H]^+^ 838.4416, calcd. for C_45_H_63_N_3_O_12_ 837.44117.

*Characterization of di-tert-butyl ((S)-2-((S)-4-amino-5-(tert-butoxy)-5-oxopentanamido)-4-(tert-butoxy)-4-oxobutanoyl)-l-glutamate* (**HT-D**). Obtained from **HT-C** by method B as a white oily liquid; Yield: 90.4%; ^1^H-NMR (500 MHz, CDCl_3_): *δ* 1.43 (d, 36H, 12 × -CH_3_), 1.82 (m, 2H), 1.91 (m, 2H), 2.08 (m, 2H), 2.28 (m, 2H), 2.42 (m, 1H), 2.51 (m, 1H), 2.98 (m, 1H), 3.30 (m, 1H), 4.44 (d, *J* = 4.5 Hz, 1H), 4.80 (d, *J* = 3.7 Hz, 1H), 7.06 (d, *J* = 8.5 Hz, 1H), 7.49 (d, *J* = 8.0 Hz, 1H). ^13^C-NMR (126 MHz, CDCl_3_): *δ* 28.0, 28.1, 28.2, 29.7, 29.8, 31.5, 33.9, 37.1, 49.1, 52.4, 55.4, 80.6, 81.4, 81.8, 82.2, 170.7, 170.8, 171.8, 172.3, 172.7, 174.9. HRMS (ESI) *m*/*z*: [M + H]^+^ 616.3830, calcd. for C_30_H_53_N_3_O_10_ 615.37309.

*Characterization of tetra-tert-butyl (5S,10S,15S,18S)-15-(2-(tert-butoxy)-2-oxoethyl)-1-(9H-fluoren-9-yl)-3,8,13,16-tetraoxo-2-oxa-4,9,14,17-tetraazaicosane-5,10,18,20-tetracarboxylate* (**HT-E**). Obtained from **HT-D** by method B as a white powder; m.p. 91.6–92.2 °C; Yield: 86.7%; ^1^H-NMR (500 MHz, CDCl_3_): *δ* 1.40 (s, 9H, 3 × -CH_3_), 1.41 (s, 9H, 3 × -CH_3_), 1.43 (s, 9H, 3 × -CH_3_), 1.45 (s, 9H, 3 × -CH_3_), 1.46 (s, 9H, 3 × -CH_3_), 1.89 (m, 4H), 2.25 (m, 10H), 2.62 (dd, *J* = 17.2, 6.8 Hz, 1H), 2.84 (dd, *J* = 17.2, 4.7 Hz, 1H), 4.23 (m, 1H), 4.40 (m, 2H), 4.48 (m, 1H), 4.76 (m, 1H), 5.62 (d, *J* = 8.2 Hz, 1H), 6.58 (d, *J* = 7.6 Hz, 1H), 7.13 (d, *J* = 7.7 Hz, 1H), 7.18 (d, *J* = 7.7 Hz, 1H), 7.30 (m, 2H), 7.38 (m, 2H), 7.62 (d, *J* = 7.4 Hz, 2H), 7.75 (d, *J* = 7.5 Hz, 2H). ^13^C-NMR (126 MHz, CDCl_3_): *δ* 27.7, 28.0, 28.1, 28.2, 28.6, 28.8, 31.5, 32.1, 32.3, 37.2, 42.1, 47.3, 49.5, 52.3, 52.5, 53.9, 67.1, 80.7, 81.8, 82.2, 82.5, 82.6, 120.1, 125.3, 127.2, 127.8, 141.4, 141.5, 143.8, 144.1, 156.5, 170.5, 170.6, 171.2, 171.3, 171.3, 172.0, 172.2, 172.2. HRMS (ESI) *m*/*z*: [M + H]^+^ 1023.5528, calcd. for C_54_H_78_N_4_O_15_ 1022.54637.

*Characterization of di-tert-butyl ((S)-2-((S)-4-((S)-4-amino-5-(tert-butoxy)-5-oxopentanamido)-5-(tert-butoxy)-5-oxopentanamido)-4-(tert-butoxy)-4-oxobutanoyl)-l-glutamate* (**HT-F**). Obtained from **HT-E** by method B as a white oily liquid; Yield: 87.3%; ^1^H-NMR (500 MHz, CDCl_3_): *δ* 1.41 (s, 9H, 3 × -CH_3_), 1.42 (s, 9H, 3 × -CH_3_), 1.43 (s, 9H, 3 × -CH_3_), 1.44 (s, 9H, 3 × -CH_3_), 1.45 (s, 9H, 3 × -CH_3_), 1.89 (m, 4H), 2.09 (m, 2H), 2.22 (m, 2H), 2.31 (t, *J* = 7.3 Hz, 2H), 2.38 (t, *J* = 7.0 Hz, 2H), 2.61 (dd, *J* = 17.2, 6.6 Hz, 1H), 2.86 (m, 1H), 3.36 (m, *J* = 8.4, 4.8 Hz, 1H), 4.42 (m, 2H), 4.76 (m, 1H), 6.89 (d, *J* = 7.6 Hz, 1H), 7.13 (d, *J* = 7.8 Hz, 1H), 7.22 (d, *J* = 7.8 Hz, 1H). ^13^C-NMR (126 MHz, CDCl_3_): *δ* 27.7, 28.1, 28.2, 28.5, 30.2, 31.5, 32.5, 32.9, 37.2, 49.5, 52.3, 52.5, 54.5, 80.7, 81.5, 81.8, 82.3, 82.4, 170.5, 170.6, 171.2, 171.4, 172.2, 172.3, 172.8, 174.8. HRMS (ESI) *m*/*z*: [M + H]^+^ 801.4883, calcd. for C_39_H_68_N_4_O_13_ 800.47829.

*Characterization of 6-benzyl 11,16,24,26-tetra-tert-butyl (6S,11S,16S,21S,24S)-21-(2-(tert-butoxy)-2-oxoethyl)-2,2-dimethyl-4,9,14,19,22-pentaoxo-3-oxa-5,10,15,20,23-pentaazahexacosane-6,11,16,24,26-pentacarboxylate* (**HT-I**). Obtained from **HT-F** by method A as a white powder; m.p. 107.0–107.7 °C; Yield: 83.6%; ^1^H-NMR (500 MHz, CDCl_3_): *δ* 1.39 (s, 27H, 9 × -CH_3_), 1.41 (s, 18H, 6 × -CH_3_), 1.42 (s, 9H, 3 × -CH_3_), 1.74 (m, 2H), 2.13 (s, 6H), 2.14 (s, 2H), 2.22 (m, 2H), 2.27 (m, 4H), 2.62 (dd, *J* = 17.1, 6.9 Hz, 1H), 2.78 (m, *J* = 17.0, 4.8 Hz, 1H), 4.41 (m, 3H), 4.75 (dd, *J* = 12.8, 6.8 Hz, 1H), 5.14 (m, 2H), 5.57 (d, *J* = 8.3 Hz, 1H), 6.52 (m, 1H), 6.86 (d, *J* = 7.5 Hz, 1H), 7.21 (d, *J* = 7.8 Hz, 1H), 7.32 (m, 5H). ^13^C-NMR (126 MHz, CDCl_3_): *δ* 27.7, 28.0, 28.1, 28.4, 31.0, 31.5, 31.6, 32.0, 32.2, 37.3, 49.5, 52.0, 52.2, 52.4, 53.1, 67.2, 80.1, 80.6, 81.6, 82.1, 82.4, 128.4, 128.5, 128.7, 135.5, 156.0, 170.6, 170.6, 171.0, 171.1, 171.3, 171.9, 172.1, 172.2, 172.3. HRMS (ESI) *m*/*z*: [M + H]^+^ 1120.6292, calcd. for C_56_H_89_N_5_O_18_ 1119.62026.

*Characterization of (7S,10S,15S,20S,25S)-10-(2-(tert-butoxy)-2-oxoethyl)-7,15,20-tris(tert-butoxycarbonyl)-25-((tert-butoxycarbonyl)amino)-2,2-dimethyl-4,9,12,17,22-pentaoxo-3-oxa-8,11,16,21-tetraazahexacosan-26-oic acid* (**HT-J**). Obtained from **HT-I** by method C as a white powder; m.p. 94.3–95.0 °C; Yield: 92.2%; ^1^H-NMR (500 MHz, CDCl_3_): *δ* 1.41 (s, 9H, 3 × -CH_3_), 1.42 (s, 27H, 9 × -CH_3_), 1.44 (s, 9H, 3 × -CH_3_), 1.45 (s, 9H, 3 × -CH_3_), 1.87 (m, 3H), 2.08 (m, 1H), 2.24 (m, 4H), 2.33 (m, 4H), 2.40 (m, 4H), 2.73 (m, 2H), 4.29 (m, 1H), 4.43 (m, 2H), 4.54 (t, *J* = 7.3 Hz, 1H), 4.79 (dd, *J* = 12.7, 7.2 Hz, 1H), 5.56 (d, *J* = 7.3 Hz, 1H), 6.57 (s, 1H), 7.10 (d, *J* = 7.8 Hz, 1H), 7.34 (d, *J* = 7.9 Hz, 1H), 7.53 (d, *J* = 7.8 Hz, 1H). ^13^C-NMR (126 MHz, CDCl_3_): *δ* 27.7, 28.1, 28.2, 28.3, 28.5, 31.2, 31.5, 32.0, 32.4, 37.3, 49.5, 51.7, 52.5, 52.6, 52.8, 80.3, 81.2, 81.8, 82.1, 82.3, 82.6, 156.2, 170.7, 170.8, 171.2, 171.4, 172.2, 172.3, 172.8, 173.0, 174.0. HRMS (ESI) *m*/*z*: [M + H]^+^ 1130.5793, calcd. for C_49_H_83_N_5_O_18_ 1029.57331.

#### 4.1.4. General Synthetic Procedure of the Preparation of PSMA-Activated Prodrug **CPT-HT-J-ZLn**

##### General Synthetic Procedure of the Intermediate **CPT-A-Ln** (Method D)

The compound camptothecin (1 equiv.) was dissolved in dry DCM (25 mL), DMAP (0.1 equiv.) and the corresponding *N*-boc-amino aliphatic acid (1.2 equiv.) were added. After addition of EDCI (1.5 equiv.), the mixture was stirred at 25 °C for 12 h. The reaction mixture was diluted with 50 mL CH_2_Cl_2_, then successively washed with water and brine (10 mL each), dried over sodium sulfate and filtered, and the solvent was evaporated. Then the crude product was purified by flash chromatography (silica gel, dichloromethane:methanol = 100:1 to 40:1).

##### General Synthetic Procedure of the Intermediate **CPT-B-Ln** (Method E)

To a solution of the Boc-protected compound **CPT-B-Ln** in dry DCM (30 mL), TFA (3 mL/10 mL DCM) was added. The mixture was stirred in an ice bath for 2 h. After completion of the reaction (as monitored by TLC), the solution was evaporated and the residue was dissolved with DCM (50 mL). The organic extracts were washed with distilled water (3 × 25 mL) and brine (20 mL), dried over sodium sulfate, filtrated and evaporated. Purification was performed by flash chromatography (silica gel, dichloromethane:methanol = 20:1 to 5:1).

##### General Synthetic Procedure of the Intermediate **CPT-HT-J-Ln** (Method F)

The corresponding intermediate **CPT-B-Ln** (1 equiv.) was dissolved in dry DCM (20 mL), EDCI (1.5 equiv.) and **HT-J** (1.1 equiv.) were added. After addition of HOBT (1.5 equiv.) and DIPEA (2.5 equiv.), the mixture was stirred at 25 °C for 4 h. The reaction mixture was diluted with 50 mL CH_2_Cl_2_, then successively washed with water and brine (10 mL each), dried over sodium sulfate and filtered, and the solvent was evaporated. Then the crude product was purified by flash chromatography (silica gel, dichloromethane:methanol = 1:0 to 50:1).

##### General Synthetic Procedure of the Prodrug **CPT-HT-J-ZLn** (Method G)

To a solution of the intermediate **CPT-HT-J-Ln** in 20 mL mixed solution (THF:Thioanisole:H_2_O = 95:2.5:2.5), the mixture was stirred at room temperature for 2.5 h. After completion of the reaction (as monitored by RP-HPLC), the filtrate was concentrated in vacuum and the residue was purified by PR-HPLC (A = H_2_O (0.2% TFA), and B = CH_3_CN (0.2% TFA); solvent gradient: 35–80% B in 25 min).

*Characterization of (S)-4-ethyl-3,14-dioxo-3,4,12,14-tetrahydro-1H-pyrano [3′,4′:6,7]indolizino[1,2-b]quinolin-4-yl(tert-butoxycarbonyl)glycinate* (**CPT-A-L_2_**). Obtained from **CPT** by method D as a faint yellow powder; mp > 220 °C; Yield: 65.0%; ^1^H-NMR (500 MHz, CDCl_3_): *δ* 0.98 (t, *J* = 7.4 Hz, 3H, -CH_3_), 1.42 (s, 9H, 3 × -CH_3_), 2.16 (m, 2H), 4.06 (dd, *J* = 18.0, 4.8 Hz, 1H), 4.21 (dd, *J* = 18.4, 6.1 Hz, 1H), 5.07 (s, 1H), 5.25 (m, 2H), 5.38 (d, *J* = 17.1 Hz, 1H), 5.67 (d, *J* = 17.1 Hz, 1H), 7.30 (s, 1H), 7.65 (t, *J* = 7.4 Hz, 1H), 7.81 (t, *J* = 7.5 Hz, 1H), 7.91 (d, *J* = 8.1 Hz, 1H), 8.23 (d, *J* = 8.4 Hz, 1H), 8.37 (s, 1H). ^13^C-NMR (126 MHz, CDCl_3_): *δ* 7.7, 28.4, 31.8, 42.5, 50.1, 67.2, 80.3, 96.5, 120.2, 128.2, 128.3, 128.5, 129.8, 130.8, 131.3, 145.7, 146.5, 148.9, 152.3, 155.7, 157.4, 167.3, 169.7. HRMS (ESI) *m*/*z*: [M + H]^+^ 506.1917, calcd. for C_27_H_27_N_3_O_7_ 505.18490.

*Characterization of (S)-4-ethyl-3,14-dioxo-3,4,12,14-tetrahydro-1H-pyrano[3′,4′:6,7]indolizino[1,2-b]quinolin-4-yl glycinate* (**CPT-B-L_2_**). Obtained from **CPT-A-L_2_** by method E as a faint yellow powder; m.p. 194.8–195.2 °C; Yield: 61.2%; ^1^H-NMR (500 MHz, CD_3_OD): *δ* 1.08 (t, *J* = 7.5 Hz, 3H, -CH_3_), 2.27 (m, 2H), 4.18 (m, 1H), 4.29 (m, 1H), 5.35 (m, 2H), 5.50 (d, *J* = 16.7 Hz, 1H), 5.64 (d, *J* = 16.7 Hz, 1H), 7.44 (s, 1H), 7.1 (t, *J* = 7.3 Hz, 1H), 7.88 (t, *J* = 7.5 Hz, 1H), 8.07 (d, *J* = 8.0 Hz, 1H), 8.15 (d, *J* = 8.0 Hz, 1H), 8.64 (s, 1H). ^13^C-NMR (126 MHz, CD_3_OD): *δ* 8.0, 31.9, 41.0, 51.6, 67.7, 79.6, 97.7, 120.6, 129.3, 129.8, 130.0, 130.9, 132.1, 133.1, 133.5, 147.4, 147.9, 149.6, 153.4, 159.0, 168.1, 168.6. HRMS (ESI) *m*/*z*: [M + H]^+^ 406.1397, calcd. for C_22_H_19_N_3_O_5_ 405.13247.

*Characterization of 14,22,24,9-tetra-tert-butyl 1-((S)-4-ethyl-3,14-dioxo-3,4,12,14-tetrahydro-1H-pyrano[3′,4′:6,7]indolizino[1,2-b]quinolin-4-yl) (4S,9S,14S,19S,22S)-19-(2-(tert-butoxy)-2-oxoethyl)-4-((tert-butoxycarbonyl)amino)-3,7,12,17,20-pentaoxo-2,8,13,18,21-pentaazatetracosane-1,9,14,22,24-pentacarboxylate* (**CPT-HT-J-L_2_**). Obtained from **CPT-B-L_2_** by method F as a faint yellow powder; m.p. 167.4–168.1 °C; Yield: 30.0%; ^1^H-NMR (500 MHz, CDCl_3_): *δ* 0.97 (t, *J* = 7.5 Hz, 3H, -CH_3_), 1.26 (s, 2H), 1.35 (s, 9H, 3 × -CH_3_), 1.37 (s, 9H, 3× -CH_3_), 1.42 (s, 36H, 12 × -CH_3_), 1.90 (m, 2H), 2.19 (m, 14H), 2.64 (m, 1H), 2.83 (m, 1H), 4.16 (m, 2H), 4.42 (m, 4H), 4.18 (s, 1H), 5.29 (s, 2H), 5.38 (d, *J* = 17.4 Hz, 1H), 5.67 (d, *J* = 17.4 Hz, 1H), 6.67 (m, 1H), 7.11 (m, 1H), 7.33 (m, 4H), 7.67 (t, *J* = 6.9 Hz, 1H), 7.83 (t, *J* = 7.7 Hz, 1H), 7.94 (d, *J* = 7.9 Hz, 1H), 8.24 (d, *J* = 8.3 Hz, 1H), 8.40 (s, 1H). ^13^C-NMR (126 MHz, CDCl_3_): *δ* 7.7, 27.7, 28.0, 28.1, 28.2, 28.4, 29.4, 31.6, 31.9, 32.5, 37.4, 41.4, 49.4, 50.2, 51.8, 52.3, 52.5, 53.9, 67.2, 79.9, 80.7, 81.6, 82.0, 82.3, 82.5, 82.6, 96.5, 120.3, 128.2, 128.3, 128.6, 129.7, 130.8, 131.4, 145.6, 146.4, 148.8, 152.3, 156.0, 157.4, 167.2, 169.5, 170.9, 171.0, 171.7, 172.3, 172.6, 172.7, 172.9. HRMS (ESI) *m*/*z*: [M + H]^+^ 1417.7008, calcd. for C_71_H_100_N_8_O_22_ 1416.69522.

*Characterization of ((S)-4-((S)-4-((S)-4-amino-5-((2-(((S)-4-ethyl-3,14-dioxo-3,4,12,14-tetrahydro-1H-pyrano[3′,4′:6,7]indolizino[1,2-b]quinolin-4-yl)oxy)-2-oxoethyl)amino)-5-oxopentanamido)-4-carboxybutanamido)-4-carboxybutanoyl)-l-aspartyl-l-glutamic acid* (**CPT-HT-J-ZL_2_**). Obtained from **CPT-HT-J-L_2_** by method G as a faint yellow powder; m.p. 179.0–179.5 °C; Yield: 19.9%; ^1^H-NMR (500 MHz, DMSO): *δ* 0.93 (t, *J* = 7.4 Hz, 3H, -CH_3_), 1.52 (d, *J* = 6.6 Hz, 2H), 1.76 (s, 3H), 1.96 (s, 6H), 2.16 (s, 6H), 2.26 (m, 5H), 4.15 (m, 4H), 4.58 (m, 2H), 5.27 (s, 1H), 5.35 (s, 1H), 7.18 (s, 1H), 7.73 (t, *J* = 7.5 Hz, 1H), 7.88 (t, *J* = 7.5 Hz, 1H), 8.06 (d, *J* = 7.1 Hz, 1H), 8.16 (m, 6H), 8.27 (d, *J* = 6.6 Hz, 1H), 8.71 (s, 1H), 8.93 (s, 1H). ^13^C-NMR (101 MHz, DMSO): *δ* 7.6, 26.2, 27.1, 29.9, 30.4, 31.5, 31.7, 36.2, 36.5, 49.3, 51.3, 51.5, 51.7, 66.3, 76.6, 95.1, 119.0, 127.8, 128.0, 128.7, 128.9, 129.8, 130.5, 131.7, 145.0, 146.1, 147.9, 152.4, 156.5, 167.0, 168.6, 169.0, 171.0, 171.2, 171.4, 171.7, 173.1, 173.3, 173.4, 173.8; HRMS (ESI) *m*/*z*: [M + H]^+^ 1037.3386, calcd. for C_46_H_52_N_8_O_20_ 1036.330.

*Characterization of (S)-4-ethyl-3,14-dioxo-3,4,12,14-tetrahydro-1H-pyrano[3′,4′:6,7]indolizino[1,2-b]quinolin-4-yl 4-((tert-butoxycarbonyl)amino)butanoate* (**CPT-A-L_4_**). Obtained from **CPT** by method D as a faint yellow powder; m.p. 167.8–170.2 °C; Yield: 46.0%; ^1^H-NMR (500 MHz, CDCl_3_): *δ* 0.98 (t, *J* = 7.5 Hz, 3H, -CH_3_), 1.42 (s, 9H, 3 × -CH_3_), 1.86 (m, 2H), 2.14 (m, 2H), 2.54 (m, 2H), 3.14 (m, 2H), 5.28 (m, 1H), 5.38 (m, 1H), 5.66 (m, 1H), 7.23 (m, 1H), 7.66 (m, 1H), 7.82 (m, 1H), 7.93 (m, 1H), 8.25 (m, 1H), 8.40 (m, 1H). ^13^C-NMR (126 MHz, CDCl_3_): *δ* 7.7, 25.1, 28.5, 31.1, 31.8, 39.6, 50.1, 67.2, 76.0, 79.3, 96.1, 120.3, 128.2, 128.3, 128.6, 129.6, 130.9, 131.5, 146.1, 146.3, 148.8, 152.3, 156.2, 157.5, 167.7, 172.3; HRMS (ESI) *m*/*z*: [M + H]^+^ 534.2231, calcd. for C_29_H_31_N_3_O_7_ 533.21620.

*Characterization of (S)-4-ethyl-3,14-dioxo-3,4,12,14-tetrahydro-1H-pyrano[3′,4′:6,7]indolizino[1,2-b]quinolin-4-yl 4-aminobutanoate* (**CPT-B-L_4_**). Obtained from **CPT-A-L_4_** by method E as a faint yellow powder; mp > 220 °C; Yield: 65.3%; ^1^H-NMR (500 MHz, DMSO): *δ* 0.93 (t, *J* = 7.4 Hz, 3H, -CH_3_), 1.84 (m, 2H), 2.16 (m, 2H), 2.70 (m, 2H), 2.83 (m, 2H), 5.28 (s, 2H), 5.53 (m, 2H), 7.06 (s, 1H), 7.71 (t, *J* = 6.0 Hz, 1H), 7.86 (d, *J* = 6.7 Hz, 1H), 8.13 (d, *J* = 7.5 Hz, 1H), 8.16 (d, *J* = 7.5 Hz, 1H), 8.69 (s, 1H). ^13^C-NMR (126 MHz, DMSO): *δ* 7.7, 22.5, 30.33, 38.0, 50.3, 66.4, 76.1, 94.8, 118.9, 127.8, 128.1, 128.7, 129.0, 129.9, 130.5, 131.7, 145.4, 146.2, 148.0, 152.4, 156.6, 167.4, 171.6; HRMS (ESI) *m*/*z*: [M + H]^+^ 434.1715, calcd. for C_24_H_23_N_3_O_5_ 433.16377.

*Characterization of 1,11,16,3-tetra-tert-butyl 26-((S)-4-ethyl-3,14-dioxo-3,4,12,14-tetrahydro-1H-pyrano[3′,4′:6,7]indolizino[1,2-b]quinolin-4-yl) (3S,6S,11S,16S,21S)-6-(2-(tert-butoxy)-2-oxoethyl)-21-((tert-butoxycarbonyl)amino)-5,8,13,18,22-pentaoxo-4,7,12,17,23-pentaazahexacosane-1,3,11,16,26-pentacarboxylate* (**CPT-HT-J-L_4_**). Obtained from **CPT-B-L_4_** by method F as a faint yellow powder; m.p. 129.0–129.6 °C; Yield: 49.3%; ^1^H-NMR (500 MHz, CDCl_3_): *δ* 0.95 (t, *J* = 7.4 Hz, 3H, -CH_3_), 1.39 (s, 9H, 3 × -CH_3_), 1.40 (s, 18H, 6 × -CH_3_), 1.41 (s, 18H, 6 × -CH_3_), 1.44 (s, 9H, 3 × -CH_3_), 1.72 (m, 2H), 1.84 (m, 4H), 2.15 (m, 2H), 2.26 (m, 4H), 2.37 (m, 8H), 2.56 (m, 2H), 2.83 (m, 1H), 3.30 (m, 2H), 3.64 (m, 1H), 4.11 (m, 1H), 4.43 (m, 4H), 4.79 (m, 1H), 5.29 (s, 2H), 5.39 (d, *J* = 17.2 Hz, 1H), 5.65 (d, *J* = 17.2 Hz, 1H), 5.79 (m, 1H), 6.38 (d, *J* = 8.4 Hz, 1H), 7.20 (d, *J* = 7.7 Hz, 1H), 7.33 (m, 2H), 7.53 (s, 1H), 7.66 (t, *J* = 7.4 Hz, 1H), 7.83 (t, *J* = 7.4 Hz, 1H), 7.93 (d, *J* = 8.0 Hz, 1H), 8.23 (d, *J* = 8.0 Hz, 1H), 8.40 (s, 1H). ^13^C-NMR (126 MHz, CDCl_3_): *δ* 7.7, 24.5, 24.8, 27.6, 28.1, 28.2, 28.4, 28.5, 29.0, 31.3, 31.4, 31.6, 31.9, 32.5, 37.4, 38.7, 49.4, 50.1, 51.7, 52.3, 52.5, 53.2, 53.4, 67.2, 76.0, 79.9, 80.6, 81.6, 81.9, 82.2, 82.4, 96. 4, 120.5, 128.2, 128.3, 128.7, 129.7, 130.9, 131.5, 145.9, 146.2, 148.8, 152.4, 156.2, 157.5, 167.7, 170.9, 171.0, 171.1, 171.5, 172.0, 172.1, 172.2, 172.5, 172.6, 172.7; HRMS (ESI) *m*/*z*: [M + H]^+^ 1445.7339, calcd. for C_73_H_104_N_8_O_22_ 1444.72652.

*Characterization of (3S,6S,11S,16S,21S)-21-amino-6-(carboxymethyl)-27-(((S)-4-ethyl-3,14-dioxo-3,4,12,14-tetrahydro-1H-pyrano[3′,4′:6,7]indolizino[1,2-b]quinolin-4-yl)oxy)-5,8,13,18,22,27-hexaoxo-4,7,12,17,23-pentaazaheptacosane-1,3,11,16-tetracarboxylic acid* (**CPT-HT-J-ZL_4_**). Obtained from **CPT-HT-J-L_4_** by method G as a faint yellow powder; m.p. 189.0–189.5 °C; Yield: 19.9%; ^1^H-NMR (500 MHz, DMSO): *δ* 0.92 (t, *J* = 7.4 Hz, 3H, -CH_3_), 1.73 (m, 4H), 1.92 (s, 6H), 2.16 (s, 6H), 2.22 (m, 12H), 2.63 (m, 2H), 4.11 (m, 3H), 4.55 (s, 1H), 5.31 (s, 2H), 5.48 (d, *J* = 16.9 Hz, 1H), 5.52 (d, *J* = 17.1 Hz, 1H), 7.07 (s, 1H), 7.72 (t, *J* = 7.4 Hz, 1H), 7.87 (t, *J* = 7.6 Hz, 1H), 7.93 (s, 1H), 8.09 (s, 1H), 8.15 (m, 4H), 8.24 (s, 2H), 8.48 (s, 1H), 8.70 (s, 1H). ^13^C-NMR (126 MHz, DMSO): *δ* 7.6, 24.3, 27.1, 27.2, 27.6, 29.6, 30.3, 30.6, 30.8, 31.5, 31.7, 36.3, 38.1, 50.3, 51.3, 51.4, 51.7, 51.9, 52.1, 66.4, 75.9, 94.7, 118.9, 127.8, 128.0, 128.6, 129.0, 129.9, 130.5, 131.7, 145.4, 146.1, 147.9, 152.4, 156.6, 167.4, 168.5, 170.7, 171.1, 171.5, 171.8, 172.4, 172.9, 173.6. HRMS (ESI) *m*/*z*: [M + H]^+^ 1065.3690, calcd. for C_48_H_56_N_8_O_20_ 1064.36109.

*Characterization of (S)-4-ethyl-3,14-dioxo-3,4,12,14-tetrahydro-1H-pyrano[3′,4′:6,7]indolizino[1,2-b]quinolin-4-yl 6-((tert-butoxycarbonyl)amino)hexanoate* (**CPT-A-L_6_**). Obtained from **CPT** by method D as a faint yellow powder; m.p. 152.0–152.5 °C; Yield: 55.0%; ^1^H-NMR (500 MHz, CDCl_3_): *δ* 0.97 (t, *J* = 7.5 Hz, 3H, -CH_3_), 1.39 (s, 9H, 3 × -CH_3_), 1.50 (m, 2H), 1.66 (m, 4H), 2.14 (m, 2H), 2.49 (m, 2H), 3.07 (m, 2H), 5.29 (s, 2H), 5.39 (d, *J* = 17.0 Hz, 1H), 5.67 (d, *J* = 17.0 Hz, 1H), 7.23 (s, 1H), 7.67 (m, 1H), 7.83 (m, 1H), 7.94 (d, *J* = 8.0 Hz, 1H), 8.22 (d, *J* = 8.0 Hz, 1H), 8.40 (s, 1H). ^13^C-NMR (126 MHz, CDCl_3_): *δ* 7.7, 24.4, 26.2, 28.5, 29.9, 32.0, 33.8, 40.4, 50.1, 67.2, 75.9, 96.3, 120.5, 128.2, 128.3, 128.4, 128.6, 129.6, 130.9, 131.5, 146.2, 146.2, 148.9, 152.4, 157.5, 167.7, 172.7; HRMS (ESI) *m*/*z*: [M + H]^+^ 562.2548, calcd. for C_31_H_35_N_3_O_7_ 561.24750.

*Characterization of (S)-4-ethyl-3,14-dioxo-3,4,12,14-tetrahydro-1H-pyrano[3′,4′:6,7]indolizino[1,2-b]quinolin-4-yl 6-aminohexanoate* (**CPT-B-L_6_**). Obtained from **CPT-A-L_6_** by method E as a faint yellow powder; m.p. 207.5–208.0 °C; Yield: 65.0%; ^1^H-NMR (500 MHz, CD_3_OD): *δ* 1.05 (t, *J* = 7.4 Hz, 3H, -CH_3_), 1.48 (m, 2H), 1.70 (m, 4H), 2.23 (m, 2H), 2.62 (m, 2H), 2.91 (m, 2H), 5.27 (s, 2H), 5.47 (d, *J* = 16.7 Hz, 1H), 5.61 (d, *J* = 16.7 Hz, 1H), 7.35 (s, 1H), 7.70 (m, 1H), 7.87 (t, *J* = 7.0 Hz, 1H), 8.04 (d, *J* = 8.0 Hz, 1H), 8.15 (d, *J* = 8.0 Hz, 1H), 8.60 (s, 1H). ^13^C-NMR (126 MHz, CD_3_OD): *δ* 8.1, 25.1, 26.6, 28.2, 32.1, 34.2, 40.5, 51.5, 67.7, 77.5, 97.8, 120.6, 129.2, 129.7, 129.7, 130.0, 130.9, 132.0, 133.4, 147.5, 148.4, 149.5, 153.4, 159.0, 169.6, 173.9. HRMS (ESI) *m*/*z*: [M + H]^+^ 462.2030, calcd. for C_26_H_27_N_3_O_5_ 461.19507.

*Characterization of 1,11,16,3-tetra-tert-butyl 28-((S)-4-ethyl-3,14-dioxo-3,4,12,14-tetrahydro-1H-pyrano[3′,4′:6,7]indolizino[1,2-b]quinolin-4-yl) (3S,6S,11S,16S,21S)-6-(2-(tert-butoxy)-2-oxoethyl)-21-((tert-butoxycarbonyl)amino)-5,8,13,18,22-pentaoxo-4,7,12,17,23-pentaazaoctacosane-1,3,11,16,28-pentacarboxylate* (**CPT-HT-J-L_6_**). Obtained from **CPT-B-L_6_** by method F as a faint yellow powder; m.p. 138.5–139.0 °C; Yield: 45.2%; ^1^H-NMR (500 MHz, CDCl_3_): *δ* 0.96 (t, *J* = 7.5 Hz, 3H, -CH_3_), 1.41 (s, 27H, 9 × -CH_3_), 1.42 (s, 9H, 3 × -CH_3_), 1.43 (s, 9H, 3 × -CH_3_), 1.45 (s, 9H, 3 × -CH_3_), 1.52 (m, 2H), 1.76 (m, 6H), 2.12 (m, 4H), 2.32 (m, 12H), 2.48 (m, 2H), 2.61 (m, 1H), 2.86 (m, 1H), 3.21 (m, 2H), 4.11 (s, 1H), 4.45 (m, 3H), 4.80 (m, 1H), 5.30 (s, 2H), 5.40 (d, *J* = 17.2 Hz, 1H), 5.66 (d, *J* = 17.2 Hz, 1H), 5.84 (d, *J* = 7.0 Hz, 1H), 6.34 (d, *J* = 8.3 Hz, 1H), 7.23 (d, *J* = 7.4 Hz, 1H), 7.27 (s, 1H), 7.34 (t, *J* = 8.3 Hz, 1 H), 7.67 (t, *J* = 7.4 Hz, 1H), 7.84 (t, *J* = 7.2 Hz, H), 7.94 (d, *J* = 8.0 Hz, 1H), 8.25 (d, *J* = 8.5 Hz, 1 H), 8.41 (s, 1H). ^13^C-NMR (126 MHz, CDCl_3_): *δ* 7.7, 24.4, 26.3, 27.6, 27.9, 28.1, 28.2, 28.6, 29.0, 29.2, 31.6, 32.0, 32.1, 32.5, 33.7, 37.4, 39.4, 49.4, 50.1, 51.7, 52.3, 52.6, 53.3, 67.2, 75.8, 79.9, 80.6, 81.6, 81.9, 82.2, 82.5, 96.5, 120.6, 128.3, 128.4, 128.7, 129.5, 131.0, 131.7, 146.1, 148.6, 152.3, 156.2, 157.5, 167.7, 170.9, 171.1, 171.5, 171.7, 172.2, 172.5, 172.7, 172.8. HRMS (ESI) *m*/*z*: [M + H]^+^ 1473.7646, calcd. for C_75_H_108_N_8_O_22_ 1472.75782.

*Characterization of (3S,6S,11S,16S,21S)-21-amino-6-(carboxymethyl)-29-(((S)-4-ethyl-3,14-dioxo-3,4,12,14-tetrahydro-1H-pyrano[3′,4′:6,7]indolizino[1,2-b]quinolin-4-yl)oxy)-5,8,13,18,22,29-hexaoxo-4,7,12,17,23-pentaazanonacosane-1,3,11,16-tetracarboxylic acid* (**CPT-HT-J-ZL_6_**). Obtained from **CPT-HT-J-L_6_** by method G as a faint yellow powder; m.p. 190.0–190.4 °C; Yield: 25.0%; ^1^H-NMR (500 MHz, DMSO): *δ* 0.93 (t, *J* = 7.5 Hz, 3H, -CH_3_), 1.36 (m, 4H), 1.48 (m, 2H), 1.60 (m, 2H), 1.79 (m, 3H), 1.94 (m, 5H), 2.21 (m, 9H), 2.36 (m, 1H), 2.66 (m, 1H), 3.11 (m, 2H), 3.62(m, 1H), 3.72(s, 1H), 4.20 (m, 3H), 4.60 (m, 1H), 5.31 (s, 2H), 5.49 (m, 2H), 7.06 (s, 1H), 7.72 (t, *J* = 7.1 Hz, 1H), 7.87 (t, *J* = 7.7 Hz, 1H), 8.07 (d, *J* = 6.8 Hz, 1H), 8.15 (m, 5H), 8.33 (m, 2H), 8.70 (s, 1H). ^13^C-NMR (126 MHz, DMSO): *δ* 7.6, 24.0, 25.7, 27.0, 27.7, 28.5, 29.7, 30.0, 30.3, 30.4, 31.6, 31.7, 33.1, 36.2, 38.6, 49.3, 50.3, 51.3, 51.5, 51.7, 51.8, 54.9, 66.4, 75.7, 94.7, 118.9, 127.8, 128.1, 128.6, 129.0, 129.9, 130.5, 131.7, 145.5, 146.0, 147.9, 152.3, 156.6, 163.1, 167.4, 167.9, 170.9, 171.0, 171.2, 171.5, 171.7, 172.1, 172.8, 172.9, 173.1, 173.4, 173.8. HRMS (ESI) *m*/*z*: [M + H]^+^ 1093.4007, calcd. for C_50_H_60_N_8_O_20_ 1092.39239.

*Characterization of (S)-4-ethyl-3,14-dioxo-3,4,12,14-tetrahydro-1H-pyrano[3′,4′:6,7]indolizino[1,2-b]quinolin-4-yl 12-((tert-butoxycarbonyl)amino)dodecanoate* (**CPT-A-L_12_**). Obtained from **CPT** by method D as a faint yellow powder; m.p. 134.6–140.0 °C; Yield: 67.0%; ^1^H-NMR (500 MHz, CDCl_3_): *δ* 0.96 (t, *J* = 7.4 Hz, 3H, -CH_3_), 1.15 (m, 8H), 1.26 (m, 6H), 1.42 (s, 9H, 3 × -CH_3_), 1.62 (m, 2H), 2.16 (m, 1H), 2.27 (m, 1H), 2.47 (m, 2H), 3.06 (s, 2H), 5.26 (m, 2H), 5.40 (d, *J* = 17.1 Hz, 1H), 5.66 (d, *J* = 17.1 Hz, 1H), 7.22 (s, 1H), 7.66 (t, *J* = 7.3 Hz, 1H), 7.82 (t, *J* = 7.3 Hz, 1H), 7.93 (d, *J* = 8.1 Hz, 1H), 8.20 (d, *J* = 8.5 Hz, 1H), 8.39 (s, 1H). ^13^C-NMR (126 MHz, CDCl_3_): *δ* 7.7, 24.7, 26.9, 28.5, 29.1, 29.3, 29.5, 29.5, 30.1, 32.0, 33.9, 40.7, 50.0, 67.2, 75.7, 79.1, 96.2, 120.5, 128.2, 128.3, 128.3, 128.6, 129.6, 130.8, 131.4, 146.1, 146.2, 148.9, 152.4, 156.1, 157.5, 167.7, 172.9. HRMS (ESI) *m*/*z*: [M + H]^+^ 646.3484, calcd. for C_37_H_47_N_3_O_7_ 645.34140.

*Characterization of (S)-4-ethyl-3,14-dioxo-3,4,12,14-tetrahydro-1H-pyrano[3′,4′:6,7]indolizino[1,2-b]quinolin-4-yl 12-aminododecanoate* (**CPT-B-L_12_**). Obtained from **CPT-A-L_12_** by method E as a faint yellow powder; m.p. 215.5–216.0 °C; Yield: 72.0%; ^1^H-NMR (500 MHz, CD_3_OD): *δ* 1.04 (t, *J* = 7.4 Hz, 3H, -CH_3_), 1.23 (m, 8H), 1.33 (m, 6H), 1.63 (m, 4H), 2.23 (m, 2H), 2.54 (m, 2H), 2.89 (m, 2H), 5.27 (s, 2H), 5.47 (d, *J* = 16.8 Hz, 1H), 5.60 (d, *J* = 16.8 Hz, 1H), 7.35 (s, 1H), 7.69 (dd, *J* = 8.0, 7.1 Hz, 1H), 7.86 (t, *J* = 7.7 Hz, 1H), 8.04 (d, *J* = 8.2 Hz, 1H), 8.14 (d, *J* = 8.5 Hz, 1H), 8.58 (s, 1H). ^13^C-NMR (126 MHz, CD_3_OD): *δ* 8.0, 25.8, 27.4, 28.6, 30.0, 30.1, 30.3, 30.4, 30.5, 32.2, 34.6, 40.8, 51.5, 67.7, 77.3, 97.9, 120.7, 129.2, 129.7, 129.8, 130.0, 130.8, 131.9, 133.3, 147.5, 148.4, 149.6, 153.4, 159.0, 169.6, 174.1. HRMS (ESI) *m*/*z*: [M + H]^+^ 546.2972, calcd. for C_32_H_39_N_3_O_5_ 545.28897.

*Characterization of 1,11,16,3-tetra-tert-butyl 34-((S)-4-ethyl-3,14-dioxo-3,4,12,14-tetrahydro-1H-pyrano[3′,4′:6,7]indolizino[1,2-b]quinolin-4-yl) (3S,6S,11S,16S,21S)-6-(2-(tert-butoxy)-2-oxoethyl)-21-((tert-butoxycarbonyl)amino)-5,8,13,18,22-pentaoxo-4,7,12,17,23-pentaazatetratriacontane-1,3,11,16,34-pentacarboxylate* (**CPT-HT-J-L_12_**). Obtained from **CPT-B-L_12_** by method F as a faint yellow powder; m.p. 121.5–121.9 °C; Yield: 42.0%; ^1^H-NMR (500 MHz, CDCl_3_): *δ* 0.96 (t, *J* = 7.4 Hz, 3H, -CH_3_), 1.14 (m, 10H), 1.24 (s, 4H), 1.30 (m, 4H), 1.41 (s, 27H, 9 × -CH_3_), 1.42 (s, 15H, 5 × -CH_3_), 1.44 (s, 12H, 4 × -CH_3_), 1.62 (m, 4H), 1.86 (m, 2H), 2.07 (m, 4H), 2.26 (m, 6H), 2.37 (m, 4H), 2.48 (m, 4H), 2.60 (m, 1H), 2.87 (m, 1H), 3.10 (m, 1H), 3.22 (m, 1H), 4.13 (s, 1H), 4.42 (m, 2H), 4.52 (m, 1H), 4.80 (s, 1H), 5.28 (m, 2H), 5.40 (d, *J* = 17.0 Hz, 1H), 5.66 (d, *J* = 17.0 Hz, 1H), 5.87 (d, *J* = 7.1 Hz, 1H), 6.31 (d, *J* = 8.1 Hz, 1H), 7.21 (s, 1H), 7.28 (d, *J* = 7.5 Hz, 1H), 7.37 (t, *J* = 8.6 Hz, 1H), 7.44 (s, 1H), 7.66 (t, *J* = 7.4 Hz, 1H), 7.82 (t, *J* = 7.6 Hz, 1H), 7.94 (d, *J* = 8.1 Hz, 1H), 8.20 (d, *J* = 8.5 Hz, 1H), 8.40 (s, 1H). ^13^C-NMR (126 MHz, CDCl_3_): *δ* 7.7, 24.8, 27.0, 27.6, 27.8, 28.1, 28.2, 28.5, 29.0, 29.2, 29.4, 29.5, 29.6, 29.6, 31.5, 32.0, 32.1, 32.5, 33.9, 37.4, 39.8, 49.3, 50.1, 51.7, 52.2, 52.5, 53.3, 67.2, 75.7, 80.0, 80.6, 81.6, 81.9, 82.2, 82.5, 96.2, 120.5, 128.2, 128.3, 128.4, 128.6, 129.7, 130.8, 131.4, 146.1, 146.3, 149.0, 152.5, 156.3, 157.5, 167.7, 170.9, 171.1, 171.5, 171.7, 172.2, 172.5, 172.9, 172.9. HRMS (ESI) *m*/*z*: [M + H]^+^ 1557.8584, calcd. for C_81_H_120_N_8_O_22_ 1556.85172.

*Characterization of (3S,6S,11S,16S,21S)-21-amino-6-(carboxymethyl)-35-(((S)-4-ethyl-3,14-dioxo-3,4,12,14-tetrahydro-1H-pyrano[3′,4′:6,7]indolizino[1,2-b]quinolin-4-yl)oxy)-5,8,13,18,22,35-hexaoxo-4,7,12,17,23-pentaazapentatriacontane-1,3,11,16-tetracarboxylic acid* (**CPT-HT-J-ZL_12_**). Obtained from **CPT-HT-J-L_12_** by method G as a faint yellow powder; m.p. 183.9–184.3 °C; Yield: 24.8%; ^1^H-NMR (500 MHz, DMSO): *δ* 0.93 (t, *J* = 7.4 Hz, 3H, -CH_3_), 1.12 (m, 10H), 1.26 (m, 5H), 1.37 (m, 4H), 1.45 (m, 1H), 1.54 (m, 3H), 1.80 (m, 2H), 1.93 (m, 4H), 2.22 (m, 7H), 2.35 (m, 2H), 3.10 (m, 2H), 3.30 (m, 1H), 3.40 (m, 1H), 3.59 (s, 1H), 3.62 (s, 1H), 3.72 (s, 1H), 4.17 (m, 3H), 4.57 (m, 1H), 5.31 (s, 2H), 5.50 (m, 2H), 7.05 (s, 1H), 7.73 (t, *J* = 7.5 Hz, 1H), 7.87 (t, *J* = 7.5 Hz, 1H), 8.15 (m, 6H), 8.32 (m, 2H), 8.71 (s, 1H). ^13^C-NMR (126 MHz, DMSO): *δ* 7.6, 24.0, 24.5, 25.3, 26.0, 26.4, 26.7, 27.0, 27.6, 28.4, 28.7, 28.8, 28.9, 29.7, 30.3, 30.4, 33.2, 38.8, 42.0, 45.8, 50.3, 51.4, 51.6, 52.0, 66.3, 75.6, 94.7, 119.0, 127.8, 128.1, 128.6, 129.0, 129.8, 130.4, 131.6, 145.4, 146.0, 147.9, 152.3, 156.6, 163.1, 167.3, 167.9, 169.3, 171.5, 171.7, 172.1, 172.9, 172.8, 173.4, 173.6, 173.8. HRMS (ESI) *m*/*z*: [M + H]^+^ 1177.4963, calcd. for C_81_H_120_N_8_O_22_ 1176.48629.

#### 4.1.5. General Synthetic Procedure of the Preparation of PSMA Hydrolysate **CPT-D-GLn**

##### General Synthetic Procedure of the Intermediate **CPT-C-GLn** (Method H)

The corresponding intermediate **CPT-B-Ln** (1 equiv.) was dissolved in dry DCM (20 mL), EDCI (1.5 equiv.) and *N*-Cbz-l-glutamic acid 5-benzyl ester (1.1 equiv.) were added. After addition of HOBT (1.5 equiv.) and DIPEA (2.5 equiv.), the mixture was stirred at 25 °C for 4 h. The reaction mixture was diluted with 50 mL CH_2_Cl_2_, then successively washed with water and brine (10 mL each), dried over sodium sulfate and filtered, and the solvent was evaporated. Then the crude product was purified by flash chromatography (silica gel, dichloromethane:methanol = 1:0 to 50:1).

##### General Synthetic Procedure of PSMA Hydrolysate **CPT-D-GLn** (Method I)

The intermediate **CPT-C-GLn** was dissolved in 20 mL MeOH, Pd/C (10%, 80 mg) was added. The reaction mixture was stirred at room temperature for 12 h and filtered to remove Pd/C. The filtrate was concentrated in vacuum. Purification was performed by flash chromatography (silica gel, dichloromethane:methanol = 20:1 to 5:1).

*Characterization of benzyl (S)-4-(((benzyloxy)carbonyl)amino)-5-((2-(((S)-4-ethyl-3,14-dioxo-3,4,12,14-tetrahydro-1H-pyrano[3′,4′:6,7]indolizino[1,2-b]quinolin-4-yl)oxy)-2-oxoethyl)amino)-5-*oxopentanoate (**CPT-C-GL_2_**). Obtained from **CPT-B-L_2_** by method H as a faint yellow powder; m.p. 213.9–214.2 °C; Yield: 50.4%; ^1^H-NMR (500 MHz, CDCl_3_): *δ* 0.97 (t, *J* = 7.4 Hz, 3H, -CH_3_), 1.97 (dd, *J* = 14.3, 7.4 Hz, 1H), 2.14 (m, 2H), 2.26 (dd, *J* = 14.3, 7.4 Hz, 1H), 2.48 (m, 2H), 4.12 (m, 1H), 4.31 (m, 1H), 4.37 (dd, *J* = 18.2, 6.1 Hz, 1H), 4.93 (t, *J* = 14.5 Hz, 2H), 5.02 (t, *J* = 14.5 Hz, 2H), 5.13 (d, *J* = 18.9 Hz, 1H), 5.22 (d, *J* = 18.9 Hz, 1H), 5.37 (d, *J* = 17.1 Hz, 1H), 5.66 (d, *J* = 17.1 Hz, 1H), 6.99 (s, 1H), 7.18 (m, 2H), 7.24 (m, 2H), 7.28 (m, 6H), 7.63 (t, *J* = 7.5 Hz, 1H), 7.78 (t, *J* = 7.6 Hz, 1H), 7.89 (d, *J* = 8.2 Hz, 1H), 8.22 (d, *J* = 8.5 Hz, 1H), 8.30 (d, *J* = 8.5 Hz, 1H). ^13^C-NMR (126 MHz, CDCl_3_): 7.7, 28.1, 30.5, 31.8, 41.4, 50.1, 54.1, 66.7, 67.1, 67.2, 96.4, 120.2, 128.1, 128.2, 128.3, 128.4, 128.6, 128.7, 129.7, 130.8, 131.4, 135.7, 136.1, 145.6, 146.4, 148.8, 152.3, 156.4, 157.4, 167.2, 168.9, 171.7, 173.2. HRMS (ESI) *m*/*z*: [M + H]^+^ 759.2653, calcd. for C_42_H_38_N_4_O_10_ 758.25879.

*Characterization of (S)-4-amino-5-((2-(((S)-4-ethyl-3,14-dioxo-3,4,12,14-tetrahydro-1H-pyrano[3′,4′:6,7]indolizino[1,2-b]quinolin-4-yl)oxy)-2-oxoethyl)amino)-5-oxopentanoic acid* (**CPT-D-GL_2_**). Obtained from **CPT-C-GL_2_** by method I as a faint yellow powder; Yield: 39.5%; ^1^H-NMR (500 MHz, DMSO): *δ* 0.92 (s, 3H), 2.16 (d, *J* = 6.3 Hz, 2H), 2.42 (dd, *J* = 16.1, 7.8 Hz, 1H), 2.82 (d, *J* = 16.1 Hz, 1H), 3.47 (s, 1H), 4.08 (d, *J* = 17.6 Hz, 1H), 4.19 (d, *J* = 16.9 Hz, 1H), 5.27 (s, 2H), 5.50 (s, 2H), 7.19 (s, 1H), 7.71 (t, *J* = 6.5 Hz, 1H), 7.85 (d, *J* = 6.7 Hz, 1H), 8.11 (d, *J* = 7.5 Hz, 1H), 8.20 (d, *J* = 8.1 Hz, 1H), 8.67 (s, 1H), 9.02 (s, 1H). ^13^C-NMR (126 MHz, DMSO): *δ* 8.0, 28.0, 30.8, 36.4, 40.9, 50.7, 51.2, 66.8, 76.7, 95.9, 119.4, 128.2, 128.4, 129.0, 129.5, 130.2, 130.9, 132.1, 145.6, 146.4, 148.4, 152.8, 157.0, 167.6, 169.4, 171.7. HRMS (ESI) *m*/*z*: [M + H]^+^ 535.1800, calcd. for C_27_H_26_N_4_O_8_ 534.17506.

*Characterization of benzyl (S)-4-(((benzyloxy)carbonyl)amino)-5-((4-(((S)-4-ethyl-3,14-dioxo-3,4,12,14-tetrahydro-1H-pyrano[3′,4′:6,7]indolizino[1,2-b]quinolin-4-yl)oxy)-4-oxobutyl)amino)-5-oxopentanoate* (**CPT-C-GL_4_**). Obtained from **CPT-B-L_4_** by method H as a faint yellow powder; m.p. 118.0–118.7 °C; Yield: 60.0%; ^1^H-NMR (500 MHz, CDCl_3_): *δ* 0.96 (t, *J* = 7.4 Hz, 3H, -CH_3_), 1.82 (m, 2H), 1.93 (m, 1H), 2.13 (m, 2H), 2.25 (m, 1H), 2.47 (m, 4H), 3.28 (m, 2H), 4.22 (d, *J* = 4.8 Hz, 1H), 5.04 (s, 2H), 5.09 (s, 2H), 5.22 (d, *J* = 18.9 Hz, 1H), 5.27 (d, *J* = 18.9 Hz, 1H), 5.38 (d, *J* = 17.1 Hz, 1H), 5.68 (d, *J* = 17.1 Hz, 1H), 5.76 (d, *J* = 7.5 Hz, 1H), 6.58 (s, 1H), 7.23 (s, 1H), 7.30 (m, 10H), 7.65 (d, *J* = 7.5 Hz, 1H), 7.81 (dd, *J* = 11.3, 4.1 Hz, 1H), 7.92 (d, *J* = 8.1 Hz, 1H), 8.22 (d, *J* = 8.5 Hz, 1H), 8.36 (s, 1H). ^13^C-NMR (126 MHz, CDCl_3_): *δ* 7.7, 24.2, 28.2, 30.6, 31.2, 31.9, 38.7, 50.1, 54.4, 66.6, 67.1, 67.3, 76.2, 96.2, 120.2, 128.1, 128.2, 128.3, 128.4, 128.6, 128.7, 129.6, 130.9, 131.5, 135.8, 136.3, 146.0, 146.3, 148.8, 152.4, 156.4, 157.4, 168.0, 171.5, 172.3, 173.2. HRMS (ESI) *m*/*z*: [2M + H]^+^ 1573.7668, calcd. for C_44_H_42_N_4_O_10_ 786.29009.

*Characterization of (S)-4-amino-5-((4-(((S)-4-ethyl-3,14-dioxo-3,4,12,14-tetrahydro-1H-pyrano[3′,4′:6,7]indolizino[1,2-b]quinolin-4-yl)oxy)-4-oxobutyl)amino)-5-oxopentanoic acid* (**CPT-D-GL_4_**). Obtained from **CPT-C-GL_4_** by method I as a faint yellow powder; m.p. 127.5–128.1 °C; Yield: 42.6%; ^1^H-NMR (500 MHz, CDCl_3_): *δ* 0.93 (t, *J* = 7.4 Hz, 3H, -CH_3_), 1.69 (m, 2H), 1.74 (m, 1H), 1.88 (m, 1H), 2.16 (m, 2H), 2.23 (t, *J* = 7.7 Hz, 2H), 2.24 (m, 2H), 3.12 (d, *J* = 7.1 Hz, 2H), 3.33 (s, 1H), 3.95 (dd, *J* = 13.9, 8.3 Hz, 1H), 5.02 (s, 1H), 5.03 (s, 1H), 5.31 (m, 2H), 5.48 (d, *J* = 16.8 Hz, 1H), 5.52 (d, *J* = 16.8 Hz, 1H), 7.09 (s, 1H), 7.31 (dd, *J* = 8.5, 4.2 Hz, 1H), 7.36 (s, 1H), 7.73 (t, *J* = 7.1 Hz, 1H), 7.87 (t, *J* = 7.1 Hz, 1H), 7.98 (t, *J* = 5.6 Hz, 1H), 8.16 (dd, *J* = 18.4, 8.2 Hz, 2H), 8.70 (s, 1H). ^13^C-NMR (126 MHz, DMSO): *δ* 7.6, 24.4, 27.2, 30.2, 30.3, 30.7, 37.8, 50.3, 54.1, 65.4, 75.8, 94.8, 118.9, 127.7, 128.0, 128.3, 128.6, 129.0, 129.8, 130.4, 131.6, 145.4, 146.1, 147.9, 152.4, 156.6, 167.3, 171.4, 171.9, 173.9. HRMS (ESI) *m*/*z*: [M + H]^+^ 563.2149, calcd. for C_29_H_30_N_4_O_8_ 562.20636.

*Characterization of (S)-4-ethyl-3,14-dioxo-3,4,12,14-tetrahydro-1H-pyrano[3′,4′:6,7]indolizino[1,2-b]quinolin-4-yl 6-((S)-5-(benzyloxy)-2-(((benzyloxy)carbonyl)amino)-5-oxopentanamido)hexanoate* (**CPT**-**CPT-C-GL_6_**). Obtained from **CPT-B-L_6_** by method H as a faint yellow powder; m.p. 106.3–107.3 °C; Yield: 62.6%; ^1^H-NMR (400 MHz, CDCl_3_): *δ* 0.90 (t, *J* = 7.4 Hz, 3H, -CH_3_), 1.27 (m, 2H), 1.42 (m, 2H), 1.50 (m, 2H), 1.83 (m, 2H), 2.14 (m, 6H), 3.04 (m, 2H), 3.63 (s, 1H), 3.67 (s, 1H), 4.19 (m, 1H), 4.33 (m, 2H), 5.30 (d, *J* = 17.5 Hz, 1H), 5.49(d, *J* = 17.5 Hz, 1H), 7.32 (dd, *J* = 17.0, 9.6 Hz, 1H), 7.48 (d, *J* = 7.5 Hz, 1H), 7.71 (m, 1H), 7.88 (dd, *J* = 17.0, 9.6 Hz, 1H), 8.15 (m, 1H), 8.69 (s, 1H). ^13^C-NMR (101 MHz, CDCl_3_): 7.7, 24.3, 25.9, 27.8, 28.9, 30.6, 31.9, 33.7, 39.2, 50.1, 52.7, 54.3, 66.6, 67.1, 67.3, 75.9, 96.3, 120.4, 128.1, 128.3, 128.4, 128.7, 129.4, 131.0, 131.6, 135.9, 136.4, 146.1, 146.2, 148.7, 152.4, 157.4, 168.0, 171.1, 172.7, 173.2. HRMS (ESI) *m*/*z*: [M + H]^+^ 815.3279, calcd. for C_46_H_46_N_4_O_10_ 814.32139.

*Characterization of (S)-4-amino-5-((6-(((S)-4-ethyl-3,14-dioxo-3,4,12,14-tetrahydro-1H-pyrano[3′,4′:6,7]indolizino[1,2-b]quinolin-4-yl)oxy)-6-oxohexyl)amino)-5-oxopentanoic acid* (**CPT-D-GL_6_**). Obtained from **CPT-C-GL_6_** by method I as a faint yellow powder; m.p. 114.3–114.9 °C; Yield: 35.9%; ^1^H-NMR (500 MHz, CDCl_3_): *δ* 0.93 (t, *J* = 7.4 Hz, 3H, -CH_3_), 1.69 (m, 2H), 1.74 (m, 1H), 1.88 (m, 1H), 2.16 (m, 2H), 2.23 (t, *J* = 7.7 Hz, 2H), 2.24 (m, 2H), 3.12 (d, *J* = 7.1 Hz, 2H), 3.33 (s, 1H), 3.95 (dd, *J* = 13.9, 8.3 Hz, 1H), 5.02 (s, 1H), 5.03 (s, 1H), 5.31 (m, 2H), 5.48 (d, *J* = 16.8 Hz, 1H), 5.52 (d, *J* = 16.8 Hz, 1H), 7.09 (s, 1H), 7.31 (dd, *J* = 8.5, 4.2 Hz, 1H), 7.36 (s, 1H), 7.73 (t, *J* = 7.1 Hz, 1H), 7.87 (t, *J* = 7.1 Hz, 1H), 7.98 (t, *J* = 5.6 Hz, 1H), 8.16 (dd, *J* = 18.4, 8.2 Hz, 2H), 8.70 (s, 1H). ^13^C-NMR (101 MHz, DMSO): 7.6, 24.5, 25.4, 25.6, 28.7, 29.3, 30.3, 33.1, 38.3, 53.1, 54.7, 55.9, 65.5, 75.7, 94.8, 118.9, 127.3, 127.8, 128.1, 129.4, 129.9, 130.5, 131.7, 145.5, 146.0, 147.9, 152.3, 156.6, 167.3, 172.1, 173.4, 177.1, 177.4. HRMS (ESI) *m*/*z*: [M + H]^+^ 591.2467, calcd. for C_31_H_34N4_O_8_ 590.23766.

*Characterization of (S)-4-ethyl-3,14-dioxo-3,4,12,14-tetrahydro-1H-pyrano[3′,4′:6,7]indolizino[1,2-b]quinolin-4-yl 12-((S)-5-(benzyloxy)-2-(((benzyloxy)carbonyl)amino)-5-oxopentanamido)dodecanoate* (**CPT-C-GL_12_**). Obtained from **CPT-B-L_12_** by method H as a faint yellow powder; m.p. 90.7–91.5 °C; Yield: 48.6%; ^1^H-NMR (500 MHz, CDCl_3_): *δ* 0.97 (t, *J* = 7.5 Hz, 3H, -CH_3_), 1.16 (s, 10H), 1.25 (s, 2H), 1.30 (m, 2H), 1.40 (m, 2H), 1.63 (m, 2H), 1.95 (m, 1H), 2.14 (m, 1H), 2.28 (m, 1H), 2.46 (m, 4H), 3.17 (m, 2H), 4.20 (m, 1H), 5.07 (m, 2H), 5.11 (m, 2H), 5.24 (d, *J* = 19.0 Hz, 1H), 5.29 (d, *J* = 19.0 Hz, 1H), 5.40 (d, *J* = 17.0 Hz, 1H), 5.66 (d, *J* = 17.0 Hz, 1H), 5.71 (s, 1H), 6.24 (s, 1H), 7.23 (s, 1H), 7.32 (s, 6H), 7.32 (s, 4H), 7.66 (t, *J* = 7.4 Hz, 1H), 7.82 (t, *J* = 7.4 Hz, 1H), 7.93 (d, *J* = 8.1 Hz, 1H), 8.21 (d, *J* = 8.5 Hz, 1H), 8.38 (s, 1H). ^13^C-NMR (126 MHz, CDCl_3_): *δ* 7.7, 24.7, 26.9, 28.4, 29.1, 29.3, 29.3, 29.4, 29.5, 29.5, 30.5, 32.0, 33.9, 39.7, 50.0, 54.3, 66.7, 67.1, 67.2, 75.7, 96.2, 120.5, 128.1, 128.2, 128.3, 128.3, 128.4, 128.6, 128.6, 128.7, 129.6, 130.8, 131.4, 135.8, 136.3, 146.1, 146.2, 148.9, 152.4, 156.4, 157.5, 167.7, 171.0, 172.9, 173.3. HRMS (ESI) *m*/*z*: [M + H]^+^ 899.4223, calcd. for C_52_H_58_N_4_O_10_ 898.41529.

*Characterization of (S)-4-amino-5-((12-(((S)-4-ethyl-3,14-dioxo-3,4,12,14-tetrahydro-1H-pyrano[3′,4′:6,7]indolizino[1,2-b]quinolin-4-yl)oxy)-12-oxododecyl)amino)-5-oxopentanoic acid* (**CPT-D-GL_12_**). Obtained from **CPT-C-GL_12_** by method I as a faint yellow powder; m.p. 180.1–180.9 °C; Yield: 24.2%; ^1^H-NMR (500 MHz, DMSO): *δ* 0.91 (t, *J* = 7.4 Hz, 3H, -CH_3_), 1.26 (m, 16H), 1.54 (m, 2H), 1.65 (m, 2H), 1.78 (m, 2H), 2.13 (m, 4H), 2.24 (m, 2H), 3.03 (m, 2H), 3.31 (s, 1H), 5.26 (d, 2H), 5.44 (m, 1H), 5.49 (s, 1H), 6.82 (s, 1H), 7.04 (s, 1H), 7.31 (dd, *J* = 14.7, 7.1 Hz, 1H), 7.46 (d, *J* = 7.1 Hz, 1H), 7.71 (t, *J* = 7.5 Hz, 1H), 7.86 (t, *J* = 7.6 Hz, 1H), 8.04 (s, 1H), 8.13 (m, 2H), 8.69 (s, 1H). ^13^C-NMR (126 MHz, DMSO): *δ* 7.5, 24.5, 26.3, 26.4, 28.4, 28.7, 28.7, 28.8 28.9, 29.0, 29.4, 30.3, 32.3, 33.2, 38.4, 39.0, 39.2, 39.3, 39.5, 39.7, 39.8, 40.0, 50.2, 53.6, 66.3, 75.6, 94.7, 119.0, 127.7, 128.0, 128.6, 128.9, 129.8, 130.4, 131.6, 145.4, 146.0, 147.9, 152.3, 156.6, 165.1, 167.3, 172.1, 172.4, 174.7. HRMS (ESI) *m*/*z*: [M + H]^+^ 675.3390, calcd. for C_37_H_46_N_4_O_8_ 674.33156.

### 4.2. Bio-Evaluation Methods

#### 4.2.1. Cell Culture

The human prostate cancer cell line (LNCaP-FGC), human prostate cancer cell line (PC-3), human prostate cancer cell line (DU-145), human breast cancer cell line (MCF-7), human hepatocellular carcinoma cell line (HepG2), human cervical cancer cell line (Hela), were obtained from the Chinese Academy of Medical Sciences and Peking Union Medical College. The cultures of the cells were maintained as a monolayer in RPMI 1640 or DMEM supplemented with 10% (*v*/*v*) heat inactivated fetal bovine serum and 1% (*v*/*v*) enicillin/streptomycin (Corning, New York, NY, USA) and incubated at 37 °C in a humidified atmosphere with 5% CO_2_. The tested compounds were dissolved in DMSO (Sigma, St. Louis, MO, USA) and added at required concentrations to the cell culture.

#### 4.2.2. Cytotoxicity Assay

The cytotoxicity of all the tested compounds was evaluated in vitro via the MTT method against PSMA-expressing LNCaP-FGC cells and non-PSMA-expressing cells HepG2, Hela, MCF-7, DU145, PC-3 cells using **CPT** as the positive control. Tumor cells growing in the logarithmic phase were seeded in 96-well plates at a density 3 × 10^3^ cells/well and incubated overnight. The following day, cells were then treated with serial dilutions of the tested compounds for 72 h. At the end of this incubation, 20 µL of 5 mg/mL methylthiazol tetrazolium (MTT) was added to each well and incubation proceeded at 37 °C for another 4 h. After the supernatant medium was thrown away, 150 µL dimethylsulphoxide (DMSO) were added to each well and absorbance was measured at 490 nm using a plate reader (BIORAD 550 spectrophotometer, Bio-rad Life Science Development Ltd., Berkeley, CA, USA). Experiments were performed in triplicate and the values were the average of three (n = 3) independent experiments. The concentration of the compound that results in 50% growth inhibition corresponds to the IC_50_. Tumor cell growth inhibitory rate was calculated using Equation (1):

% inhibition = (1 − Sample group OD/Control group OD) × 100%(1)

#### 4.2.3. Aqueous Solubility Study

The equilibrium solubility of **CPT** and **CPT-HT-J-ZL_12_** was determined in triplicate in PBS, according to the reference method with some modifications. An excess amount (~5 mg) of the tested compounds were placed into tubes, and 1 mL PBS was added to the tube. Then the tubes were incubated at 37 °C for 24 h in an ermostated oscillator (HZQ-QX, Harbin Donglian Instrument Co., Ltd., Harbin, China). After incubation, samples (0.1 mL) were removed and centrifuged at 10,000× *g* for 10 min with high speed centrifuge (Sorvall™ Legend™ Micro 21, Thermo Fisher Scientific, Waltham, MA, USA). A 500 µL sample of the supernatant was diluted, and the concentration was measured with HPLC according the method below. **CPT** and **CPT-HT-J-ZL_12_** were measured by HPLC systems configured with a Waters 2695 separation module and a Waters 2695 ultraviolet (UV) detector with the following conditions: Xbridge C18 column (250 mm × 4.6 mm, 5 mm); temperature, 25 °C; elution flow rate, 1.0 mL/min; detection wavelength, 365 nm. mobile phase, 0.2% b in water (A) and 0.2% formic acid in acetonitrile (B) using a gradient elution of 10–60% B at 0–5.0 min, 60–80% B at 5.0–15.0 min, 80% B at 15.0–17.0 min, and 10% B at 17.1–23.0 min.

#### 4.2.4. In Vitro Cellular Uptake

To evaluate the cellular uptake of the **CPT-HT-J-ZL_12_**, LNCaP-FGC (PSMA^+^) and HepG2 (PSMA^−^) cells were seeded at a density of 1 × 10^4^ cells/well in 35 mm glass bottom dishes with 1 mL complete 1640 medium [28]. After incubation for 24 h, the culture medium was replaced with 2 mL of fresh medium containing 10 µM **CPT-HT-J-ZL_12_**. Then the cells were incubated further for different incubation time periods (1 and 4 h). Following the removal of the culture media, cells were washed with cold PBS, fixed with 70% ethanol for 30 min at 4 °C. Subsequently, the cells were stained with 5 μg/mL propidium iodide (excitation wavelength was 561 nm) for 5 min to visualize nucleus. The cells were observed by a UltraView RS confocal laser scanning microscopy (PerkinElmer, Waltham, MA, USA).

#### 4.2.5. Detection of Apoptosis Using Annexin V-FITC/PI Staining

To determine early apoptosis and secondary necrosis, LNCaP-FGC (PSMA^+^) and HepG2 (PSMA^−^) cells were stained with annexin-V FITC apoptosis detection kit (Beijing BioDee Biotech. Co., Ltd., Beijing, China) as per the manufacturer’s instructions. After exposure to different concentrations of **CPT-HT-J-ZL_12_** (0.63, 1.25, 2.50 µM for LNCaP-FGC; 20.00, 40.00, 80.00 µM for HepG2) for 72 h, the cells were collected, washed twice with cold PBS, and centrifuged at 3000× *g* for 5 min. The resulting pellet was mixed with 200 µL binding buffer of the Annexin V-FITC kit, then 5 µL FITC labeled annexin V was added and mixed gently. After incubation at 4 °C for 10 min in the dark, 5 µL PI was added and mixed gently. Then the cells were immediately analyzed with a flow cytometry.

#### 4.2.6. Detection of Cell Cycle Using PI Staining

The cell cycle was measured using PI staining and flow cytometry analysis according to the instructions of the manufacturer (Cell Cycle and Apoptosis Analysis Kit, Beyotime Biotechnology, Jiangsu, China). LNCaP-FGC cells (2 × 10^4^ cells/mL) in logarithmic growth phase were seeded in 6-well culture plate and incubated for 24 h at 37 °C in incubator with 5% CO_2_. After exposure to different concentrations of **CPT-HT-J-ZL_12_** (0.63, 1.25, 2.50 μM) for 72 h, cells were harvested and washed twice with cold PBS. Then the cells were centrifuged at 2400× *g* for 10 min, resuspended in 1 mL 70% cold ethanol and fixed for 12 h at −20 °C. After being washed twice with cold PBS, the cells were incubated with 0.5 mL propidium iodide for 30 min at 37 °C. Detection of the cell cycle was carried out by the Flow Cytometer at 488 nm excitation wavelength

#### 4.2.7. Statistical Analysis

Each experimental value is expressed as the mean ± SD (standard deviation). One-way analysis of variance (ANOVA) was performed to determine the significance between groups; *p* < 0.05 was considered to be statistically significant.

## Figures and Tables

**Table 1:** The anti-proliferative effects and ClogP values of the **CPT-HT-J-ZLn** and the prostate-specific membrane antigen (PSMA) hydrolysate **CPT-D-GLn**.

Compound	IC_50_ (µM)								CLogP
	MCF-7	PC-3	DU145	LNCaP-FGC	HepG2	HeLa	LO2	MDCK	
**CPT**	0.16 ± 0.10	0.13 ± 0.09	0.21 ± 0.09	0.18 ± 0.17	5.43 ± 0.81	2.48 ± 0.80	0.04 ± 0.01	0.02 ± 0.01	0.9
**CPT-HT-J-ZL_2_**	0.11 ± 0.08	1.00 ± 0.10	1.16 ± 0.28	2.04 ± 0.30	>40.00	3.54 ± 2.54	1.68 ± 0.45	1.27 ± 0.30	−7.45
**CPT-HT-J-ZL_4_**	0.32 ± 0.01	9.98 ± 2.38	9.73 ± 3.49	1.18 ± 0.10	>40.00	>40.00	>40.00	9.13 ± 2.40	−6.86
**CPT-HT-J-ZL_6_**	7.03 ± 3.76	40.00 ± 3.37	26.28 ± 1.81	3.13 ± 0.40	>40.00	>40.00	>40.00	18.96 ± 3.60	−6.60
**CPT-HT-J-ZL_12_**	21.68 ± 4.96	42.96 ± 3.69	5.40 ± 1.22	1.00 ± 0.20	>40.00	>40.00	>40.00	>40.00	−3.42
**CPT-B-L_2_**	4.11 ± 3.09	5.19 ± 0.30	0.12 ± 0.09	0.38 ± 0.10	>40.00	10.00 ± 2.00	5.61 ± 1.40	5.16 ± 1.20	0.54
**CPT-B-L_4_**	1.58 ± 2.42	1.00 ± 0.10	0.25 ± 0.13	0.37 ± 0.02	>40.00	4.37 ± 0.50	1.36 ± 0.30	3.20 ± 2.00	1.26
**CPT-B-L_6_**	6.76 ± 1.47	15.89 ± 1.73	3.55 ± 0.95	1.89 ± 0.20	>40.00	15.0 ± 0.80	2.71 ± 0.38	5.44 ± 3.00	1.76
**CPT-B-L_12_**	5.76 ± 0.86	6.45 ± 0.42	7.32 ± 1.43	1.93 ± 1.03	>40.00	>40.00	2.63 ± 0.84	5.2 ± 0.88	4.94
**CPT-D-GL_2_**	0.30 ± 0.05	1.5 ± 0.20	0.40 ± 0.05	3.68 ± 0.36	>40.00	>40.00	0.16 ± 0.07	0.26 ± 0.14	−2.88
**CPT-D-GL_4_**	4.03 ± 0.30	14.93 ± 1.00	4.89 ± 0.01	3.68 ± 0.50	>40.00	>40.00	0.40 ± 0.15	0.23 ± 0.10	−2.29
**CPT-D-GL_6_**	17.25 ± 3.00	12.50 ± 0.80	3.97 ± 0.16	8.17 ± 1.00	>40.00	>40.00	1.5 ± 0.60	4.0 ± 0.42	−2.03
**CPT-D-GL_12_**	>40.00	>40.00	7.38 ± 0.20	2.10 ± 0.68	>40.00	25.47 ± 1.0	3.8 ± 0.80	5.1 ± 1.10	1.15
**CPT-HT-J-L_2_**	>40.00	>40.00	>40.00	>40.00	>40.00	>40.00	>40.00	>40.00	4.04
**CPT-HT-J-L_4_**	>40.00	>40.00	>40.00	>40.00	>40.00	>40.00	>40.00	>40.00	4.62
**CPT-HT-J-L_6_**	>40.00	>40.00	>40.00	>40.00	>40.00	>40.00	>40.00	>40.00	4.89
**CPT-HT-J-L_12_**	>40.00	>40.00	>40.00	>40.00	>40.00	>40.00	>40.00	>40.00	8.07

## Supplementary Materials

Supplementary materials can be found at http://www.mdpi.com/1422-0067/19/10/3251/s1.
