# Supplementary material for: PSMA-Oriented Target Delivery of Novel Anticancer Prodrugs: Design, Synthesis, and Biological Evaluations of Oligopeptide-Camptothecin Conjugates
Source: Int J Mol Sci. 2018 Oct 19;19(10):3251. doi: 10.3390/ijms19103251 (PMC6214026; doi:10.3390/ijms19103251)
Supplement: Supplementary file 1 [file ijms-19-03251-s001.pdf]

Article

PSMA-Oriented Target Delivery of Novel Anticancer Prodrugs: Design, Synthesis, and Biological Evaluations of Oligopeptide-Camptothecin Conjugates

Bing Xu[1], Fei Zhou[1], Meng-Meng Yan[1], De-Sheng Cai[1], Wen-Bo Guo[1], Yu-Qin Yang[1], Xiao-Hui Jia[1], Wen-Xi Zhang[1], Tong Li[1], Tao Ma[1,*], Peng-Long Wang[1,*] and Hai-Min Lei[1,*]

[1] School of Chinese Pharmacy, Beijing University of Chinese Medicine, Beijing 100102, China; zf116318@163.com(F. Zhou); cai1225366978@163.com (D. Cai); wb_guo@126.com (W. Guo); yanmengmeng@bucm.edu.cn(M. Yan); yangyq5@163.com(Y. Yang); 18811508665@163.com(X. Jia); zhangwxnn@126.com(W. Zhang); lt17552258545@163.com (T. Li)

* Correspondence: mosesmatao@163.com (T. Ma), hm_lei@126.com (H. Lei), wpl581@126.com (P. Wang); Tel.: +010-84738645

Received: date; Accepted: date; Published: date

Supplementary Data

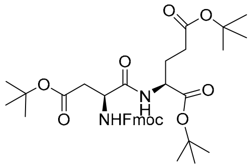

Figure S1: The structure of HT-A.

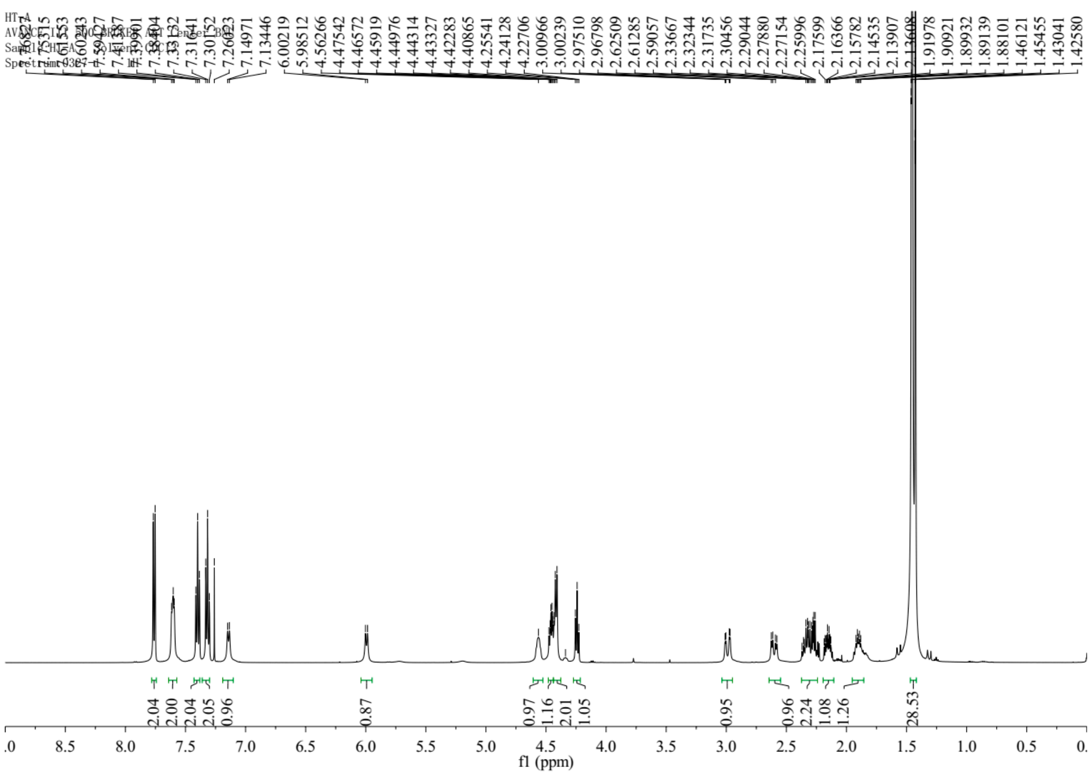

Figure S2: The ^{1}H-NMR of **HT-A**.

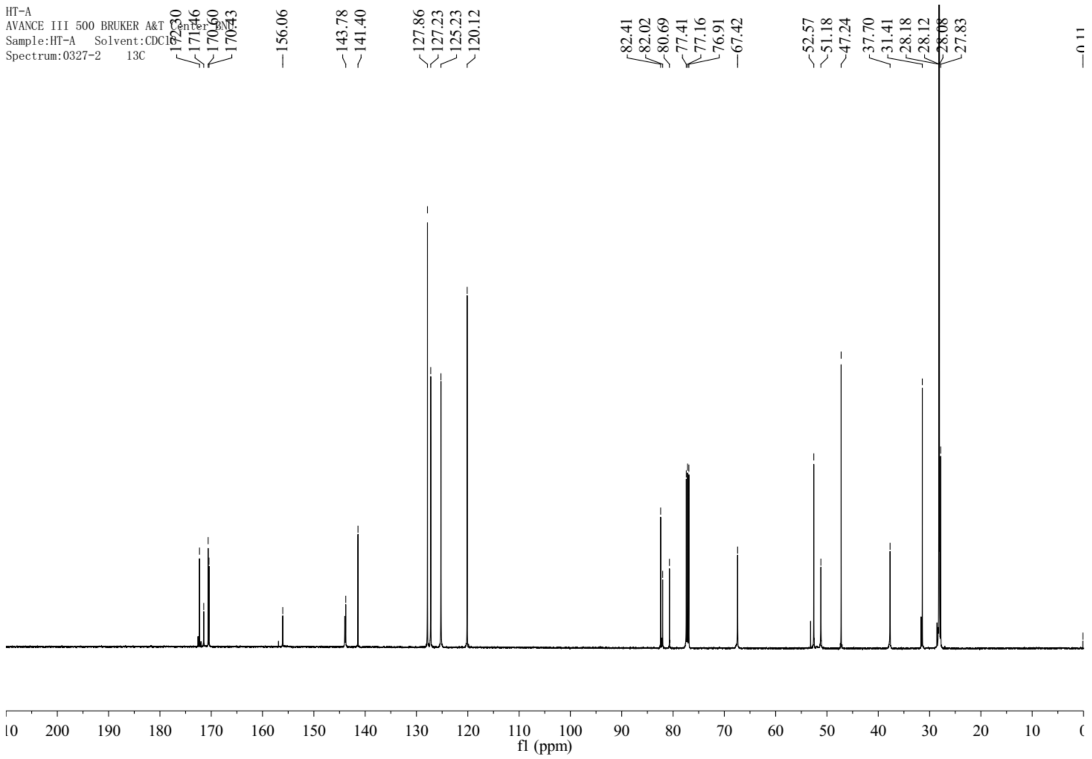

Figure S3: The ^{13}C-NMR of **HT-A**.

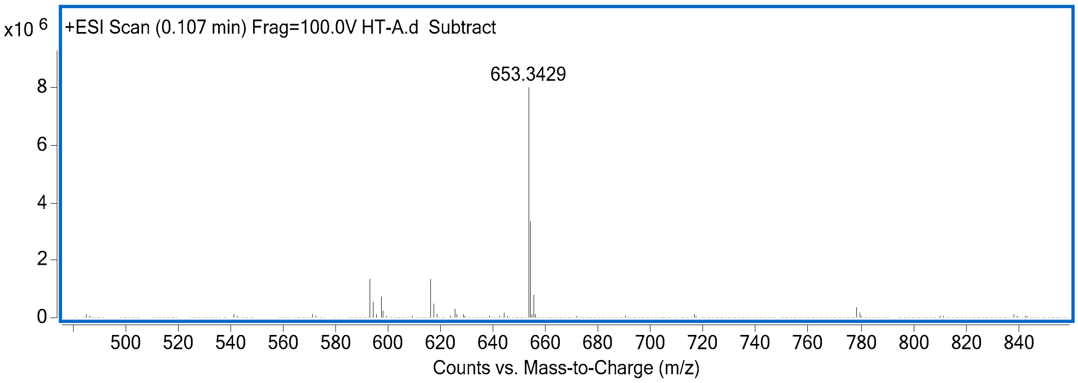

Figure S4: The HRMS of **HT-A**.

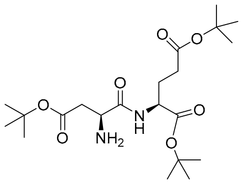

Figure S5: The structure of **HT-B**.

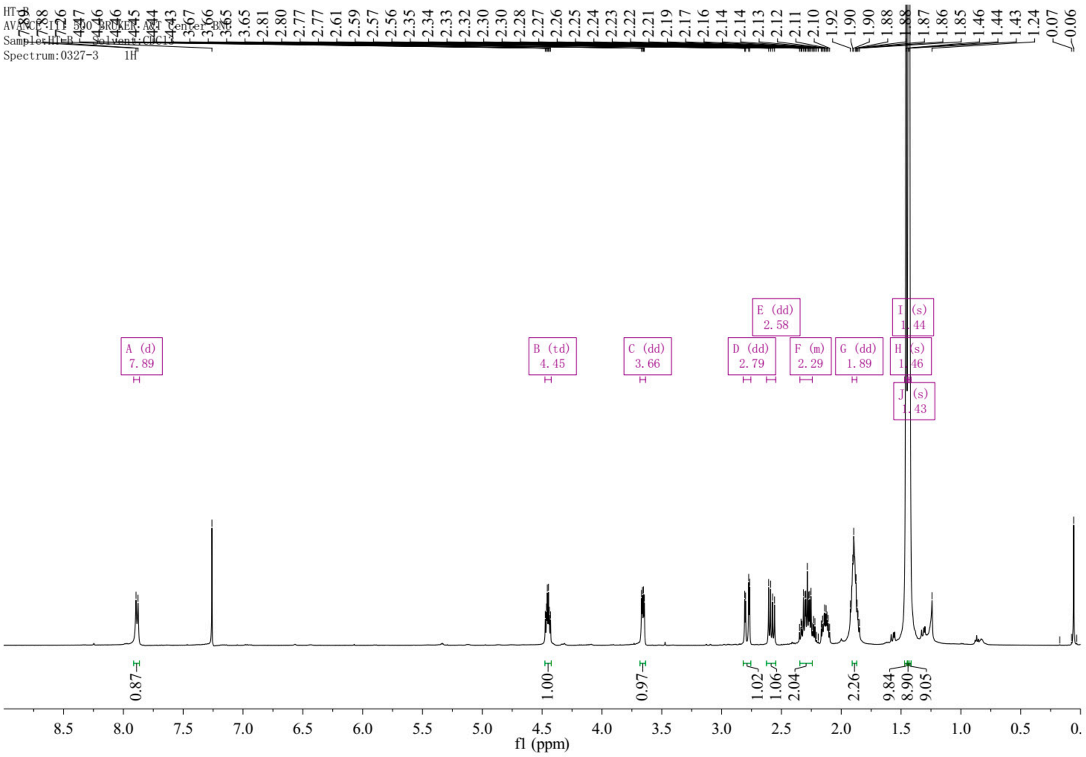

Figure S6: The ^{1}H-NMR of **HT-B**.

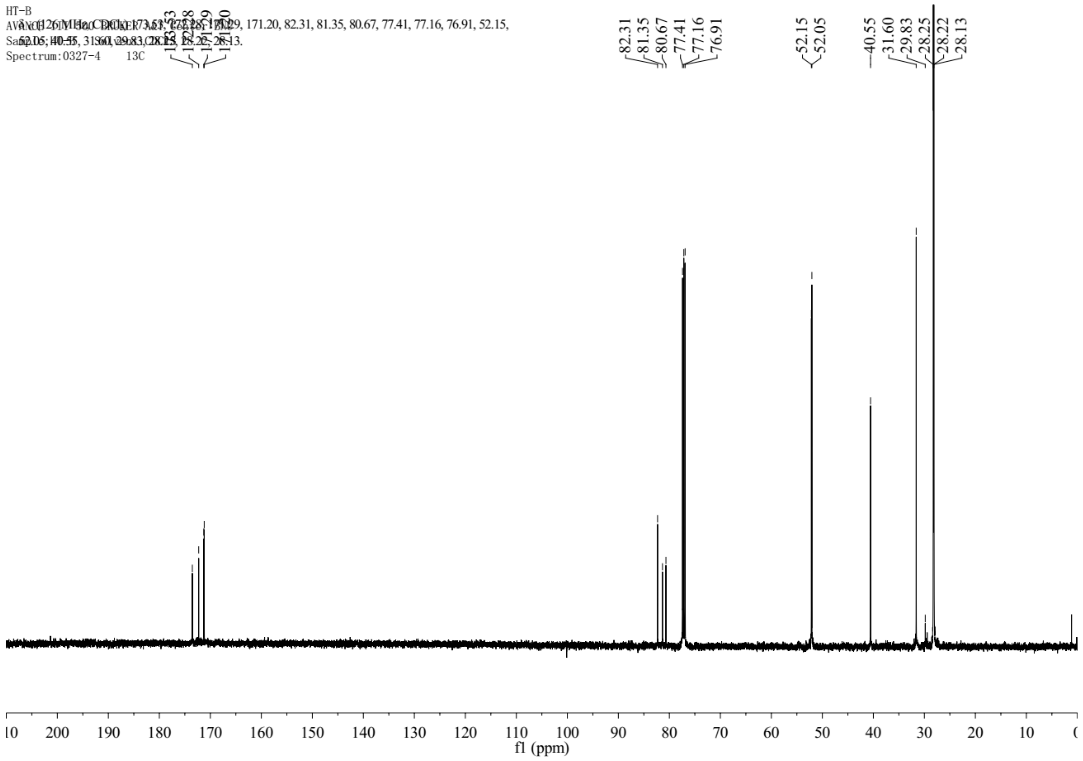

Figure S7: The ^{13}C-NMR of **HT-B**.

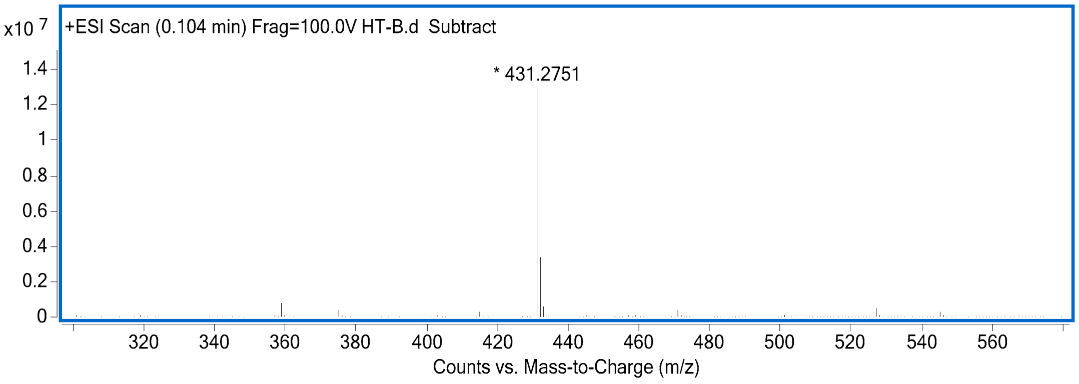

Figure S8: The HRMS of **HT-B**.

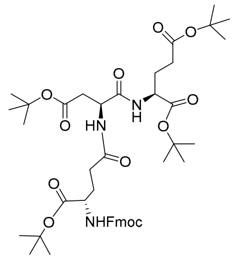

Figure S9: The structure of **HT-C**.

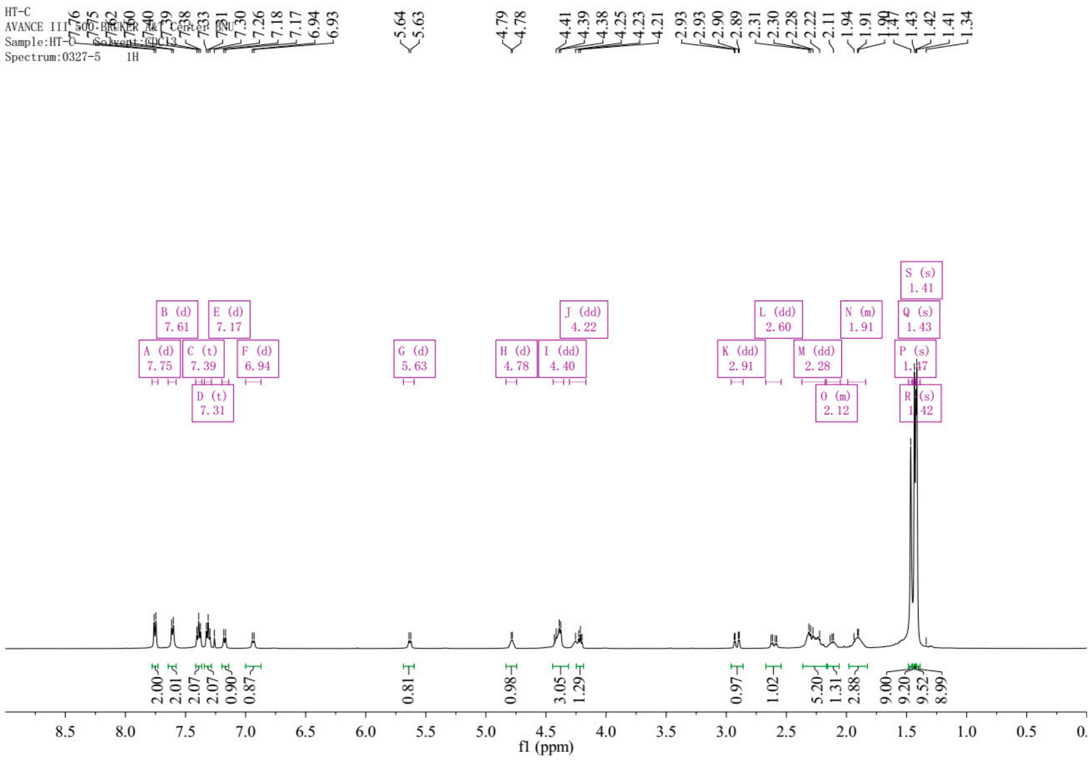

Figure S10: The ^{1}H-NMR of **HT-C**.

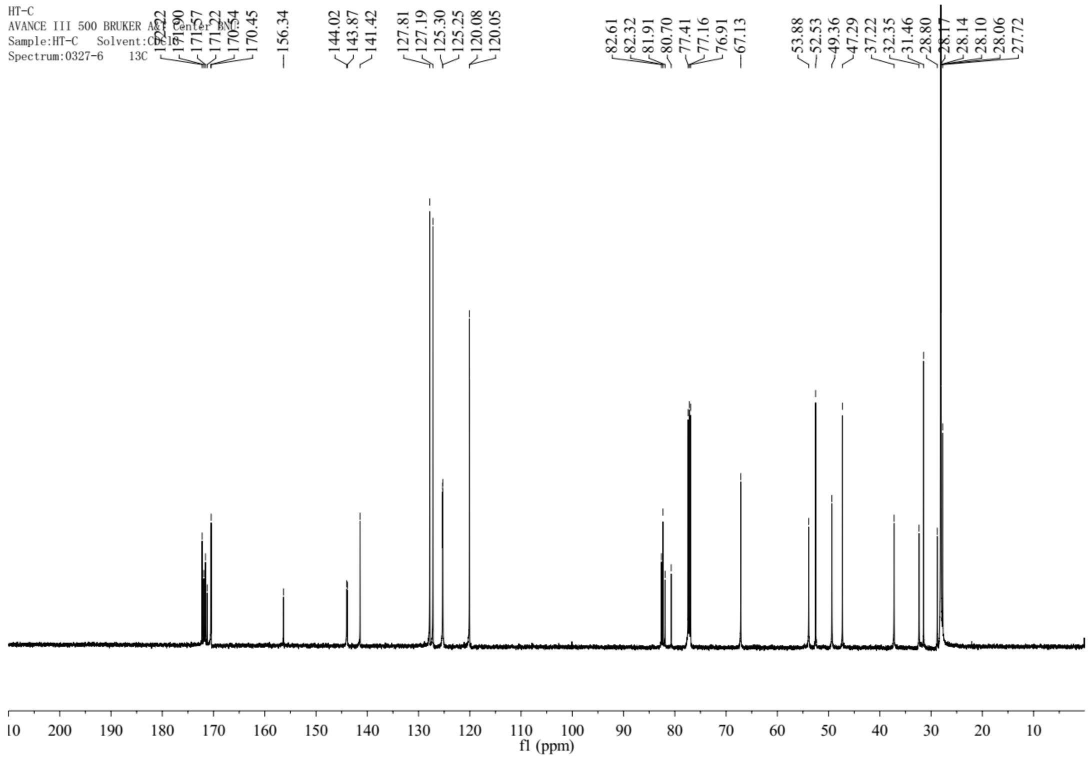

Figure S11: The ^{13}C-NMR of **HT-C**.

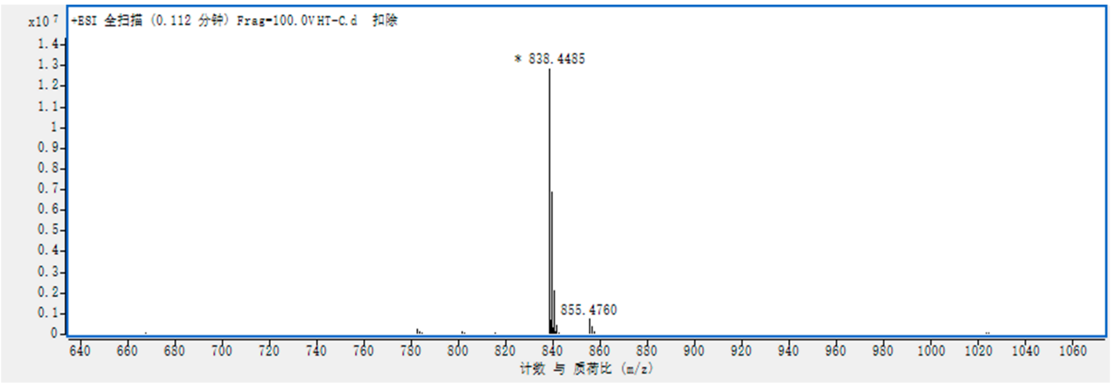

Figure S12: The HRMS of **HT-C**.

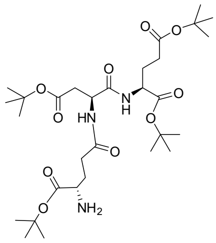

Figure S13: The structure of **HT-D**.

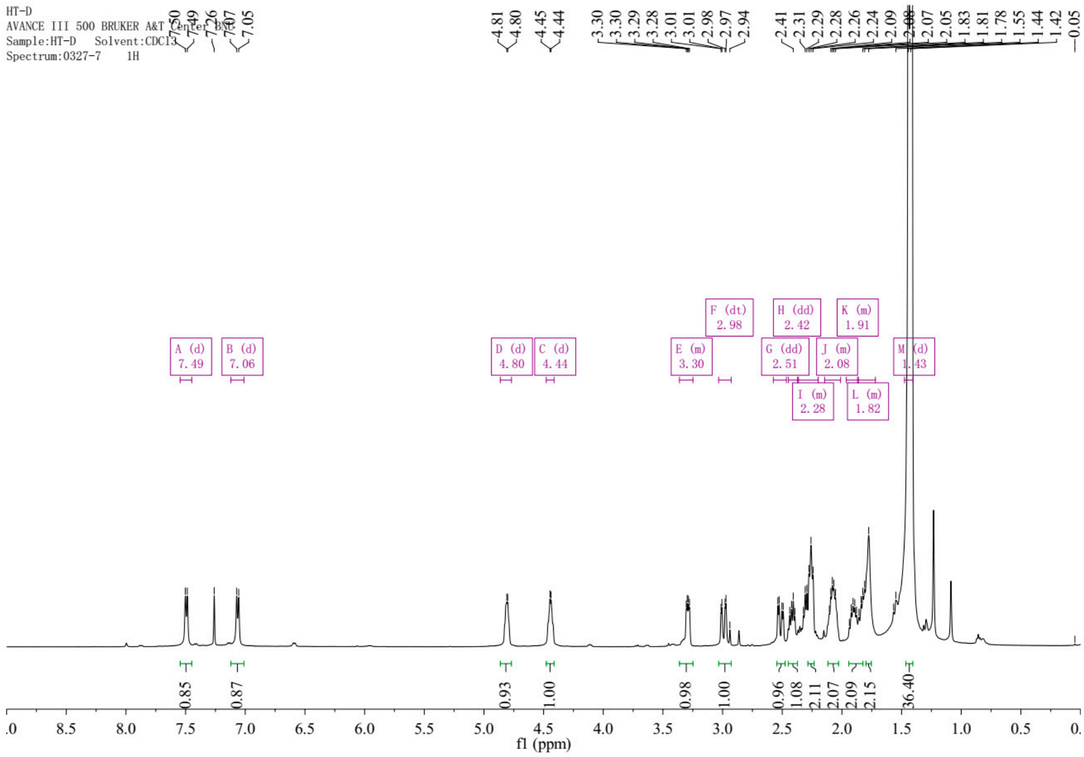

Figure S14: The ^{1}H-NMR of **HT-D**.

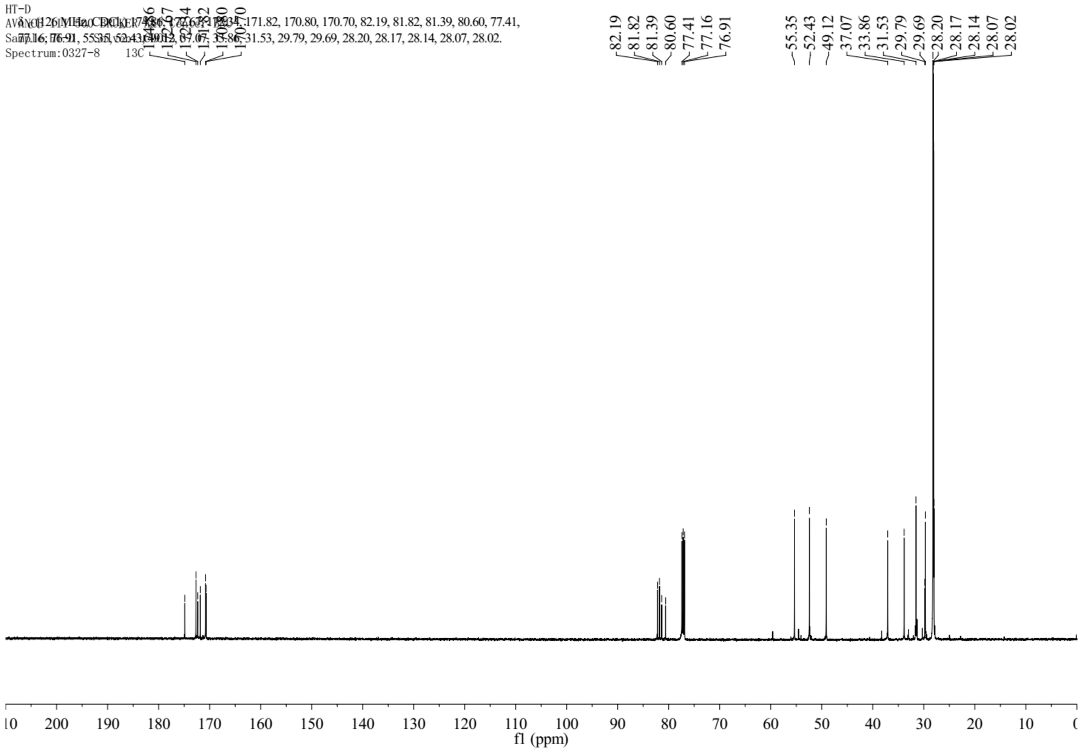

Figure S15: The ^{13}C-NMR of **HT-D**.

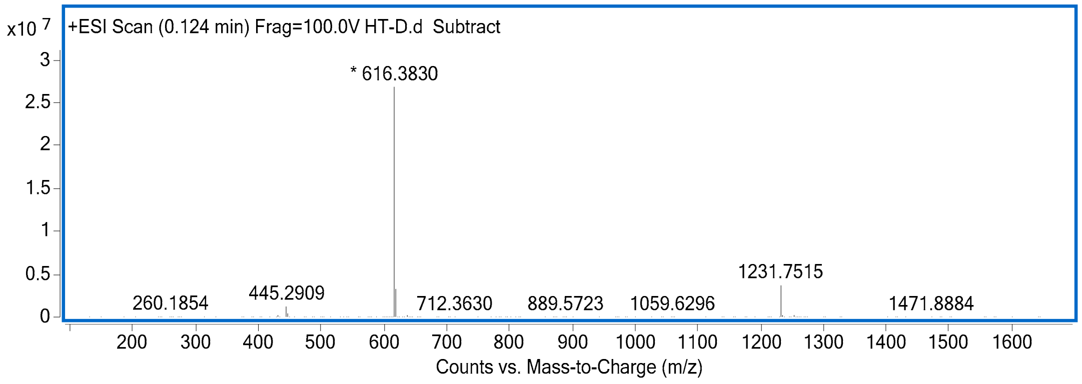

Figure S16: The HRMS of **HT-D**.

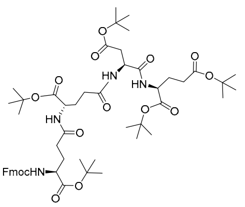

Figure S17: The structure of **HT-E**.

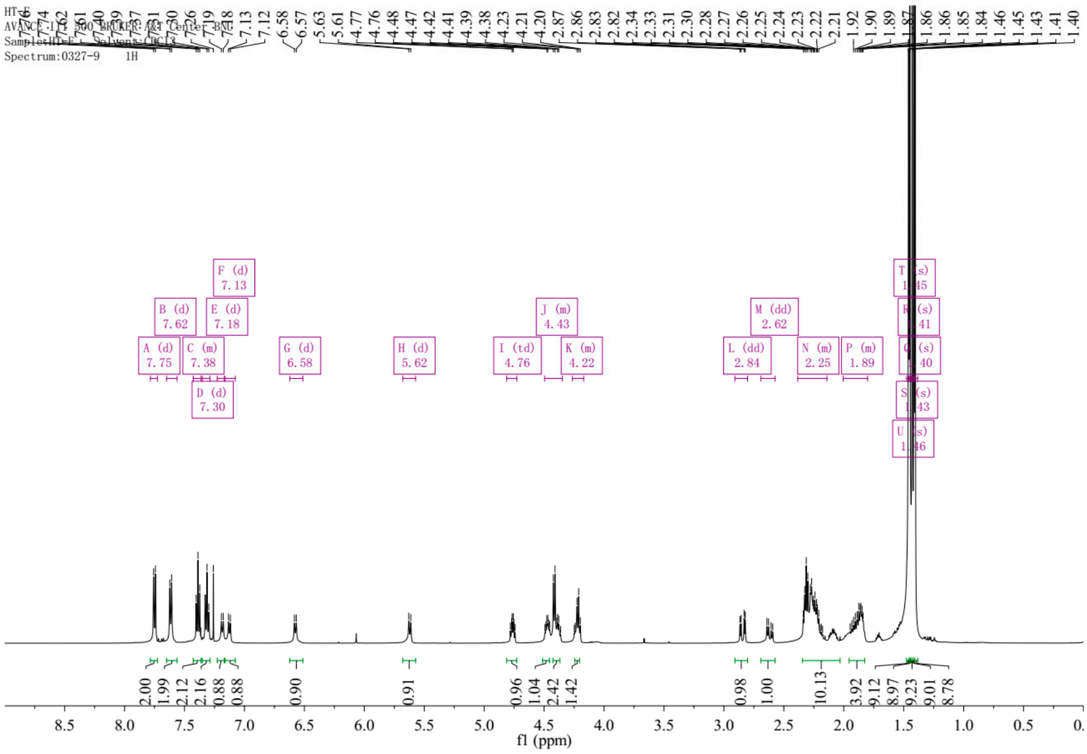

Figure S18: The ^{1}H-NMR of **HT-E**.

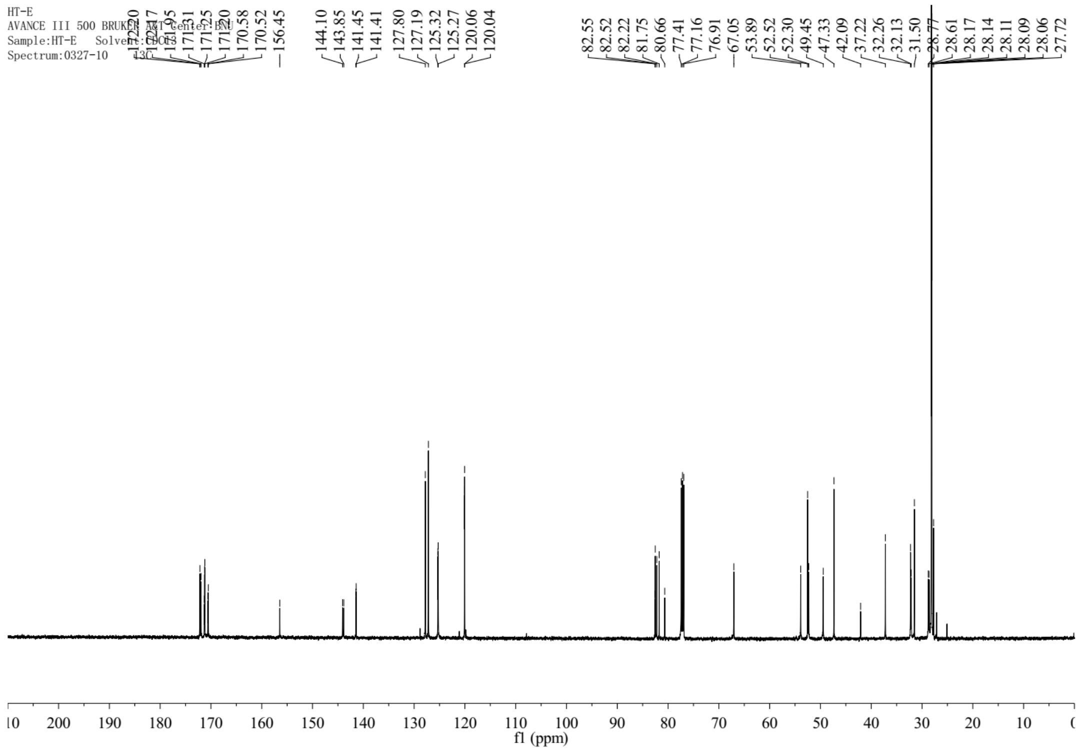

Figure S19: The ^{13}C-NMR of **HT-E**.

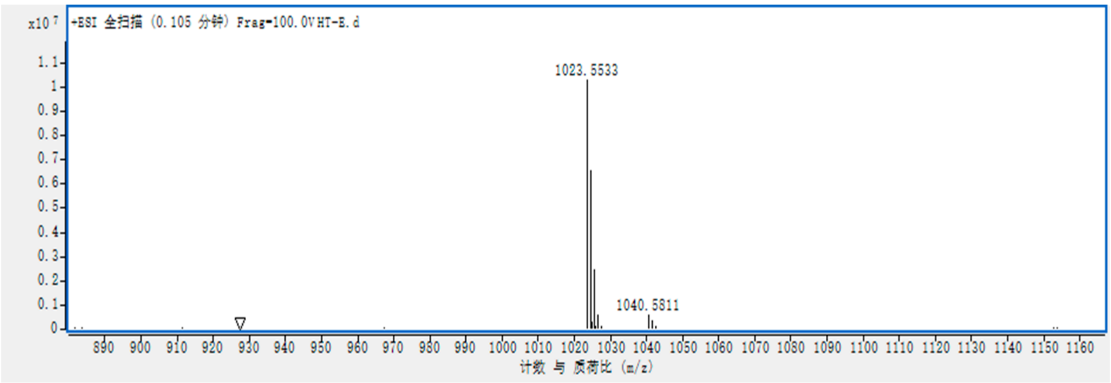

Figure S20: The HRMS of **HT-E**.

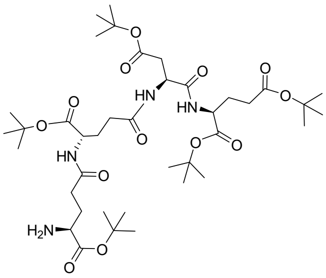

HT-F

Figure S21: The structure of **HT-F**.

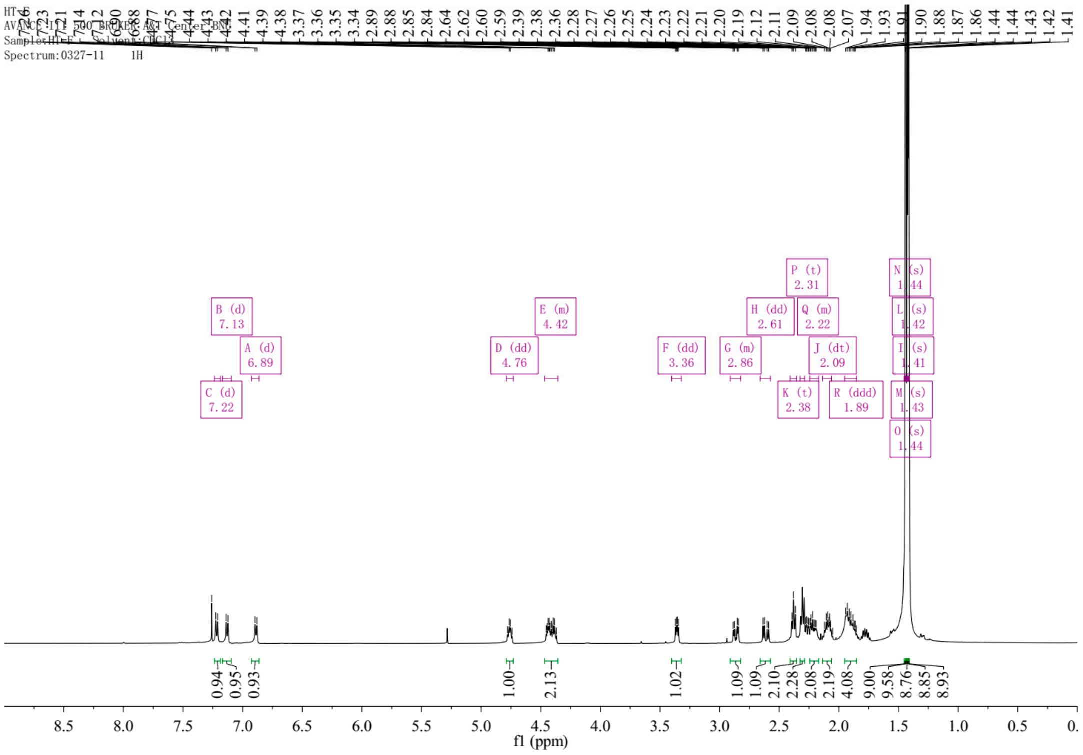

Figure S20: The ^{1}H-NMR of **HT-F**.

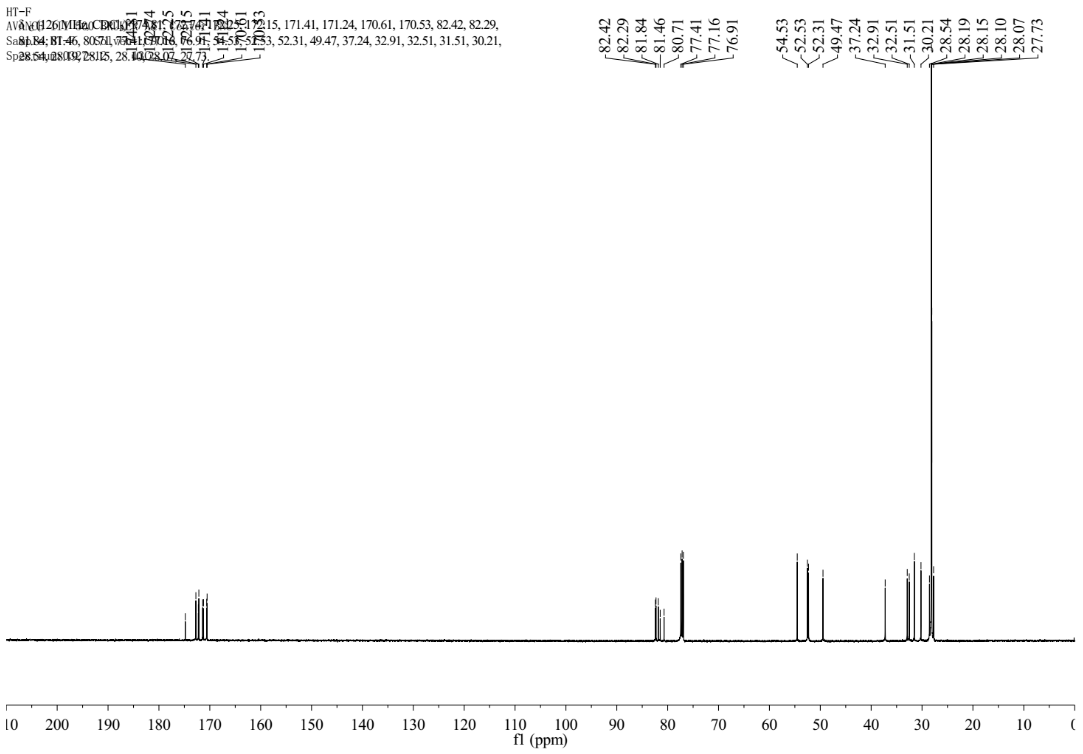

Figure S21: The ^{13}C-NMR of **HT-F**.

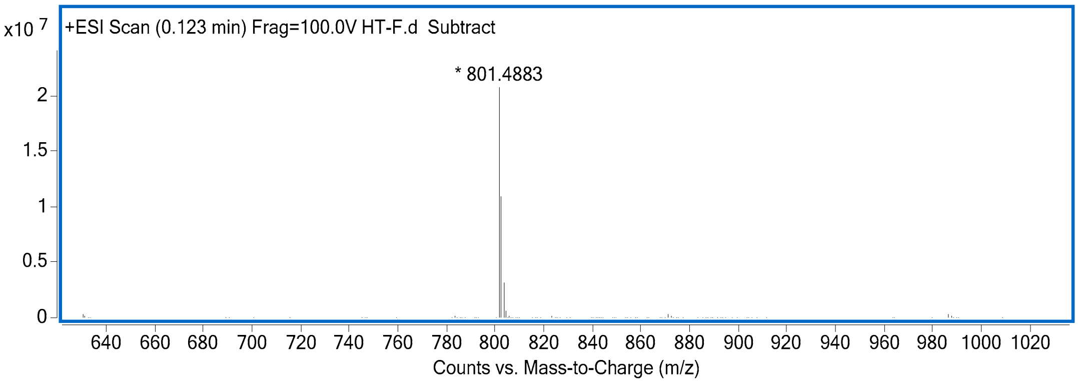

Figure S22: The HRMS of **HT-F**.

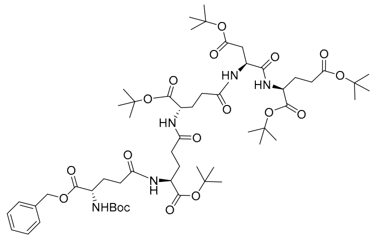

Figure S23: The structure of **HT-I**.

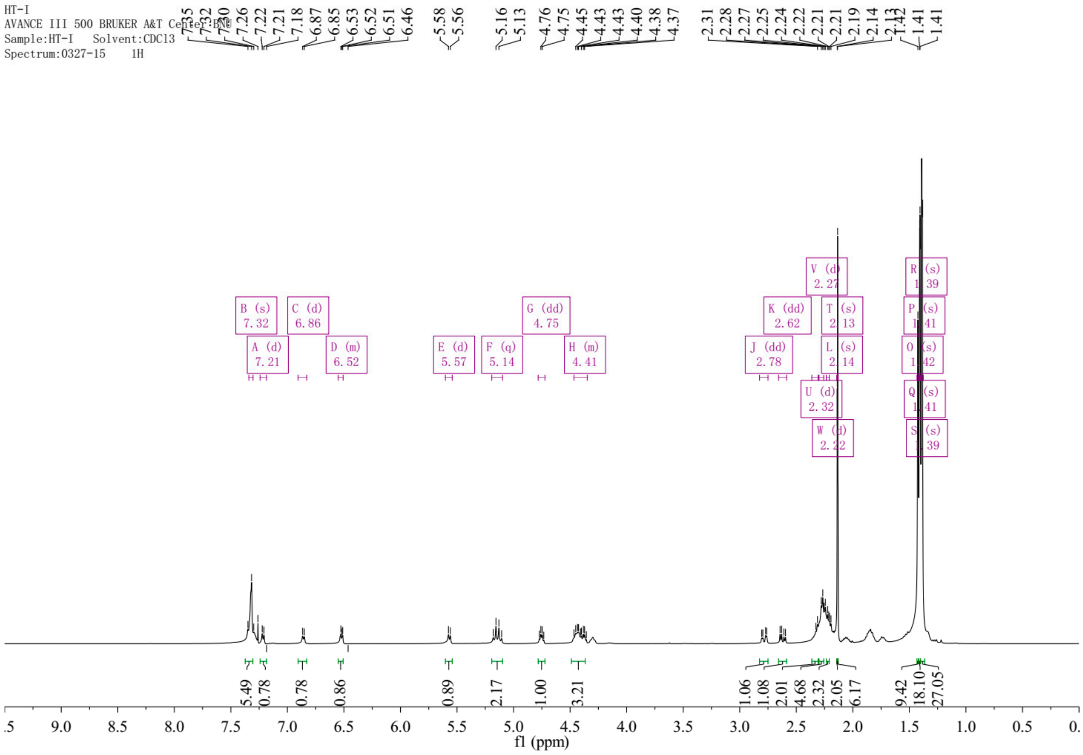

Figure S24: The ^{1}H-NMR of **HT-I**.

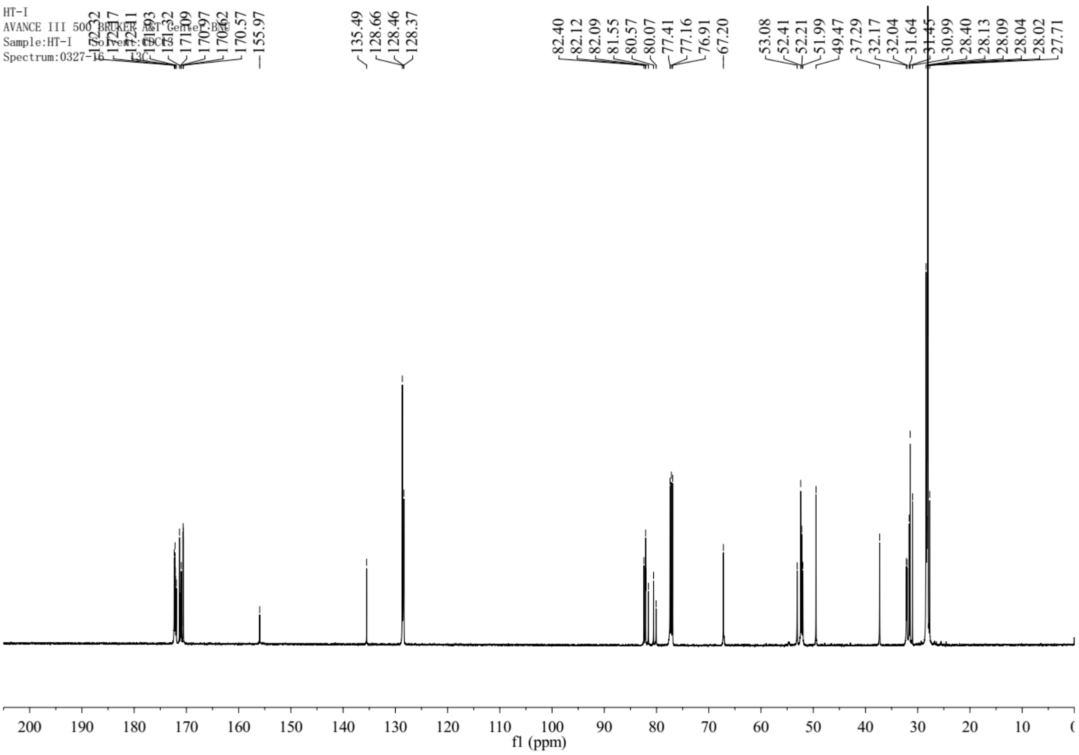

Figure S25: The ^{13}C-NMR of **HT-I**.

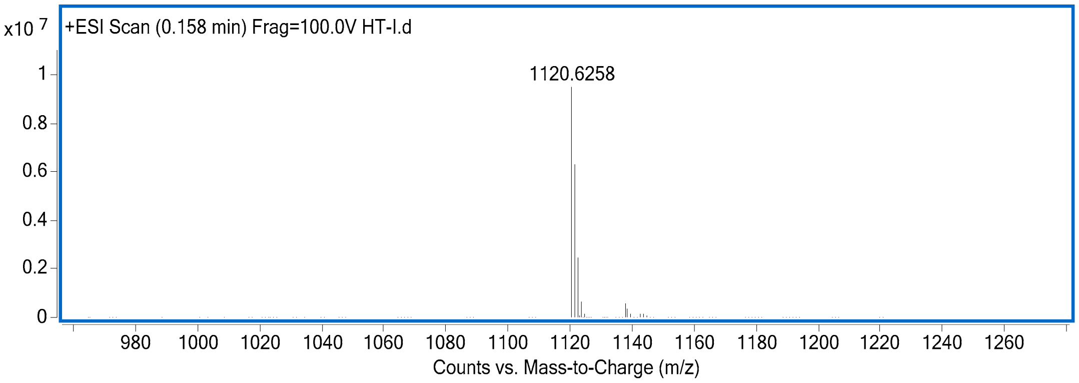

Figure S26: The HRMS of **HT-I**.

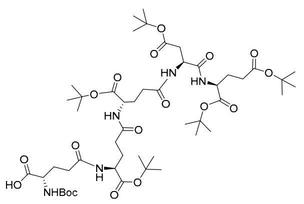

Figure S27: The structure of **HT-J**.

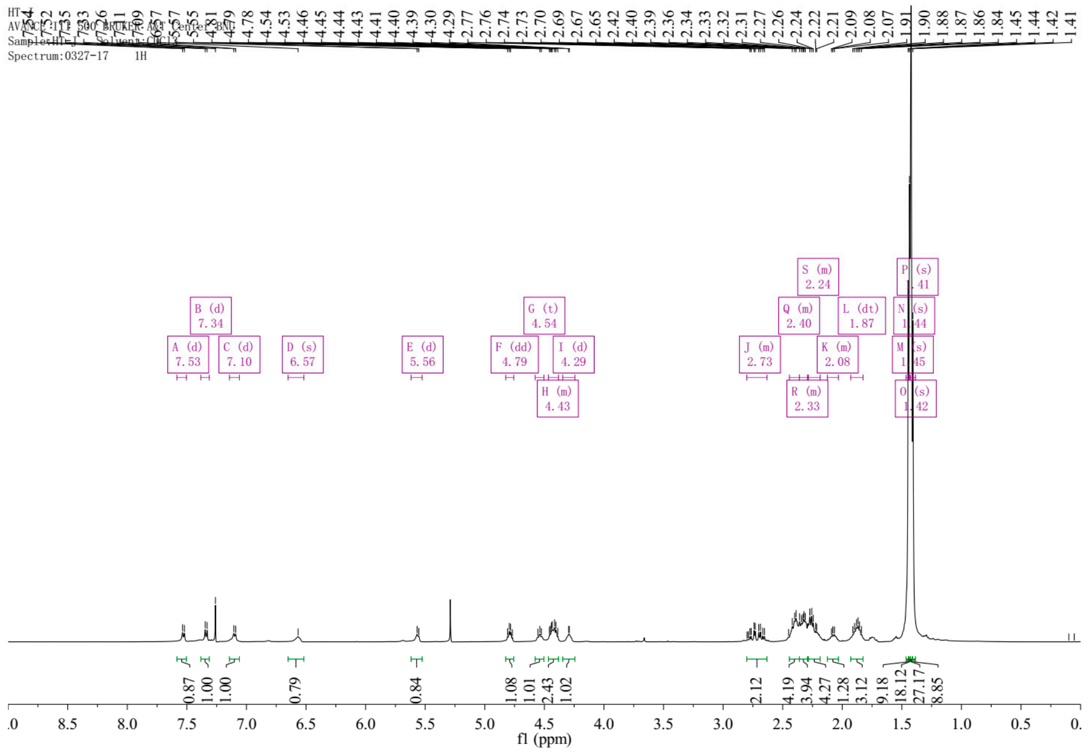

Figure S28: The ^{1}H-NMR of **HT-J**.

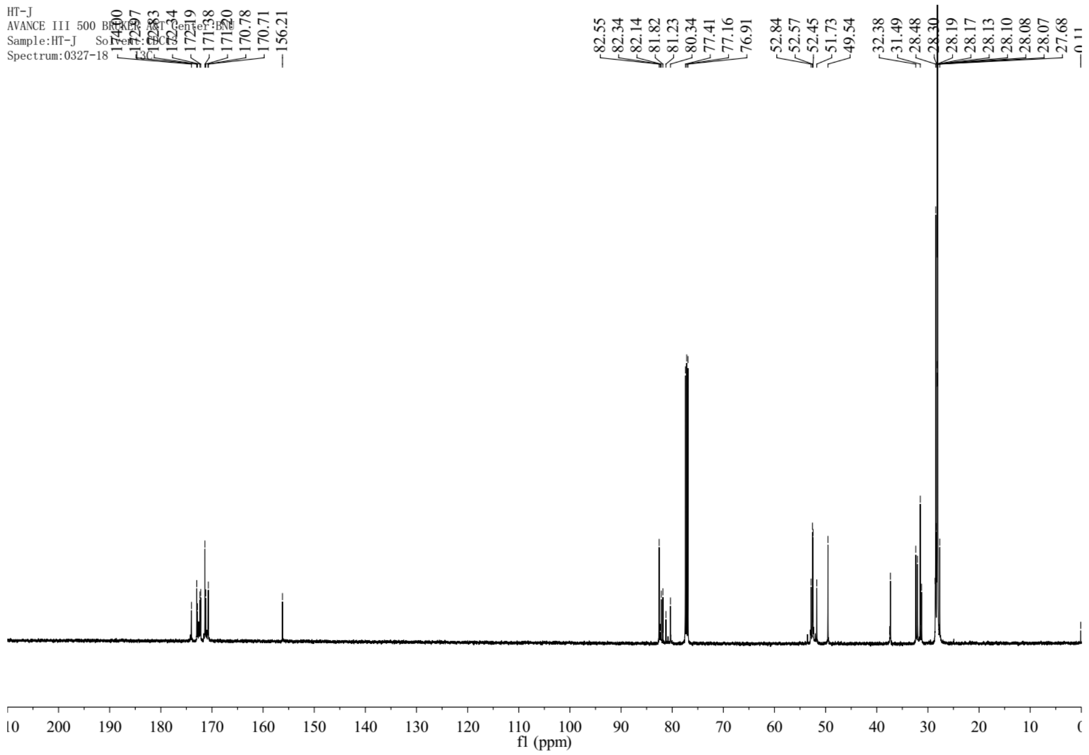

Figure S29: The ^{13}C-NMR of **HT-J**.

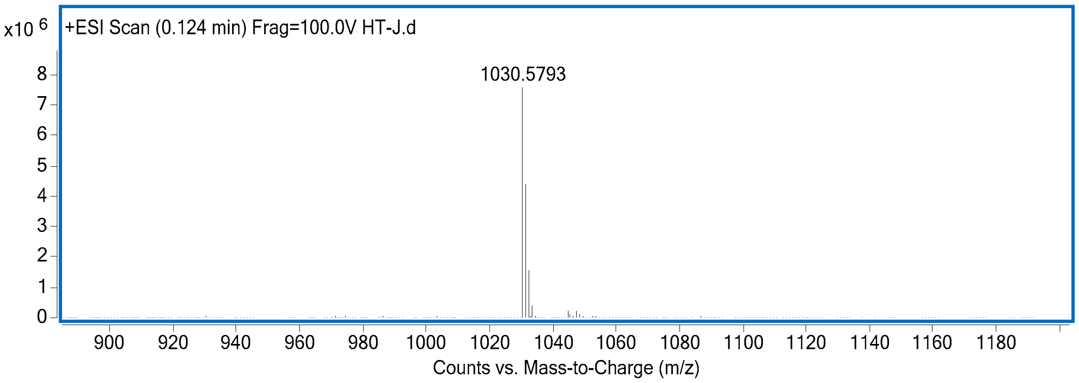

Figure S30: The HRMS of **HT-J**.

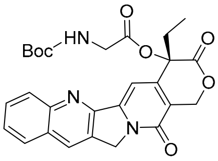

Figure S31: The structure of **CPT-A-L$_2$**.

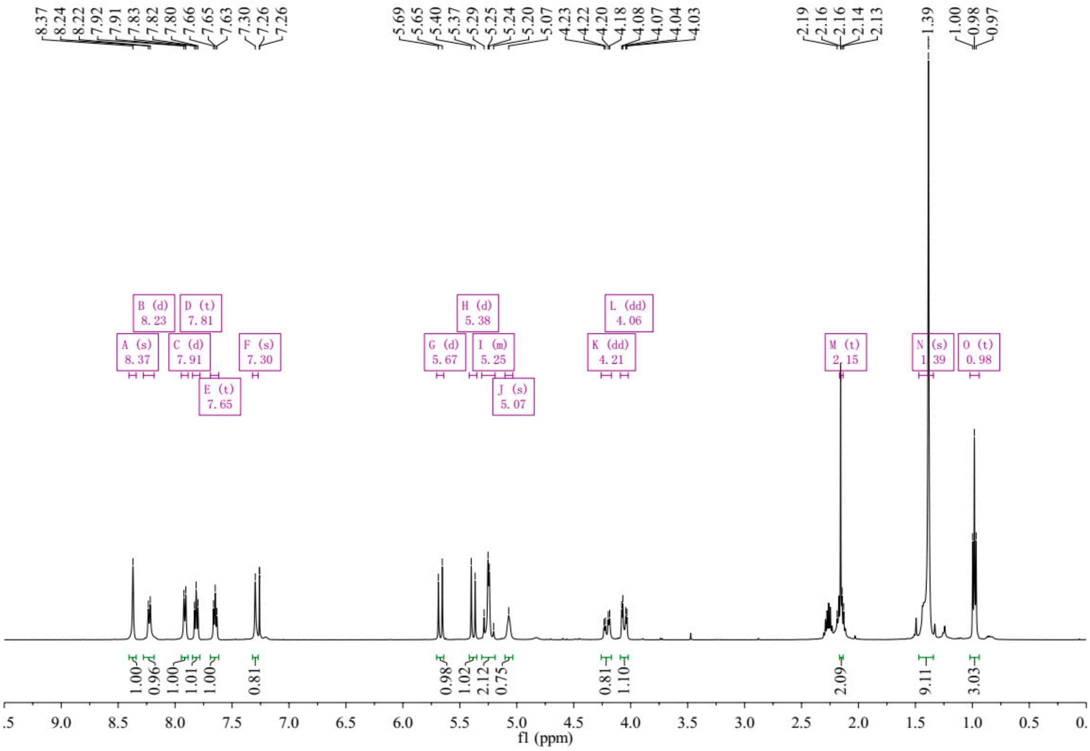

Figure S32: The ^{1}H-NMR of **CPT-A-L$_2$**.

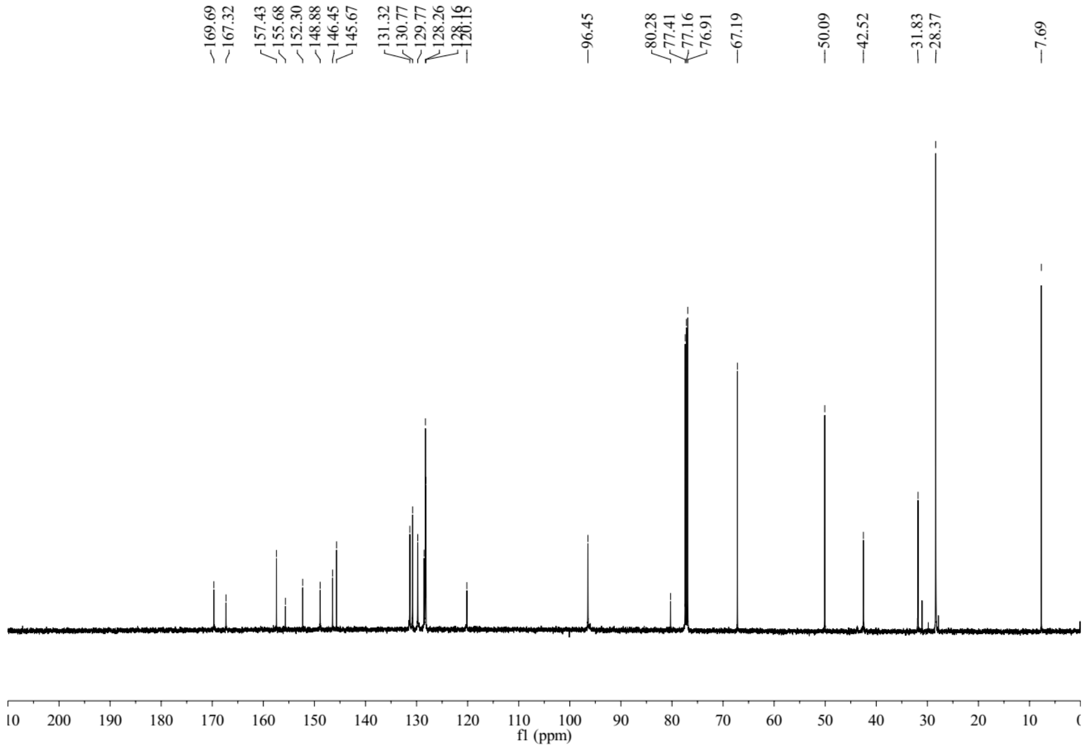

Figure S33: The ^{13}C-NMR of **CPT-A-L2**.

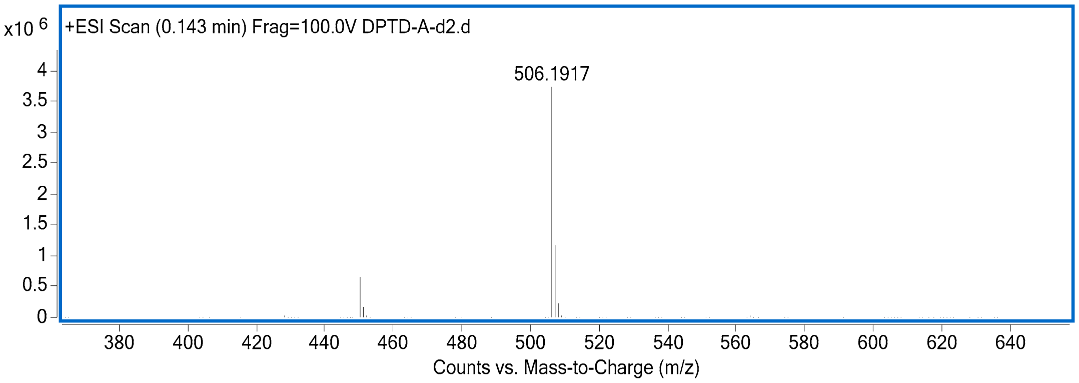

Figure S34: The HRMS of **CPT-A-L2**.

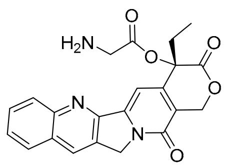

Figure S35: The structure of **CPT-B-L2**.

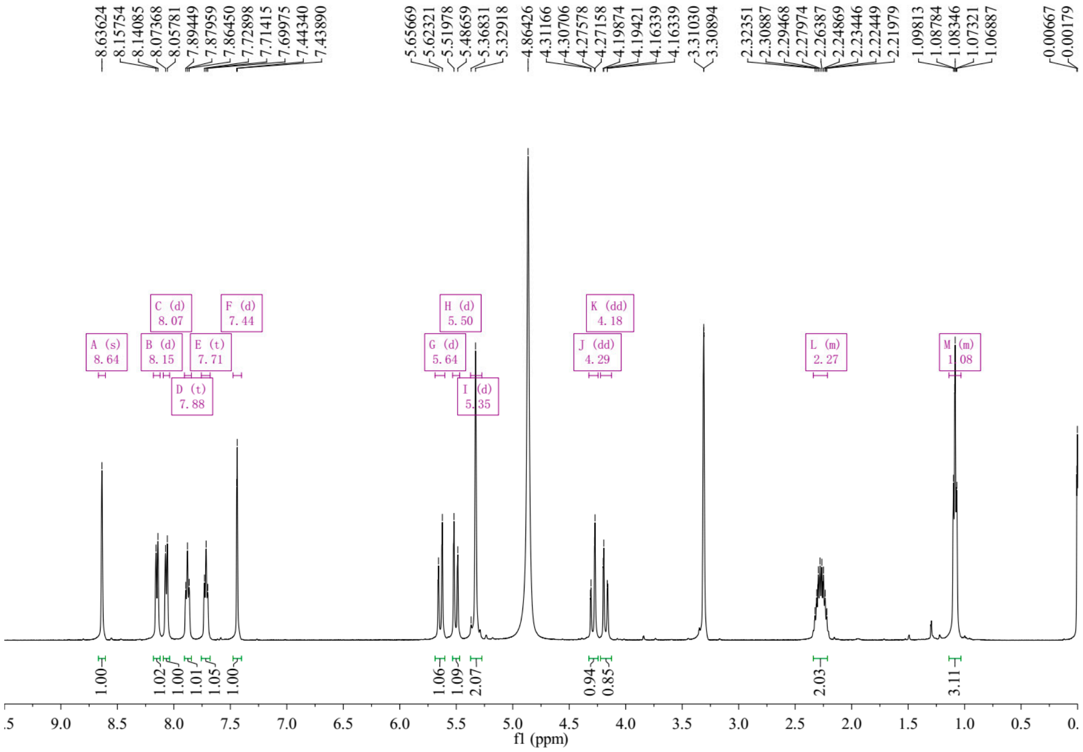

Figure S36: The ^{1}H-NMR of **CPT-B-L2**.

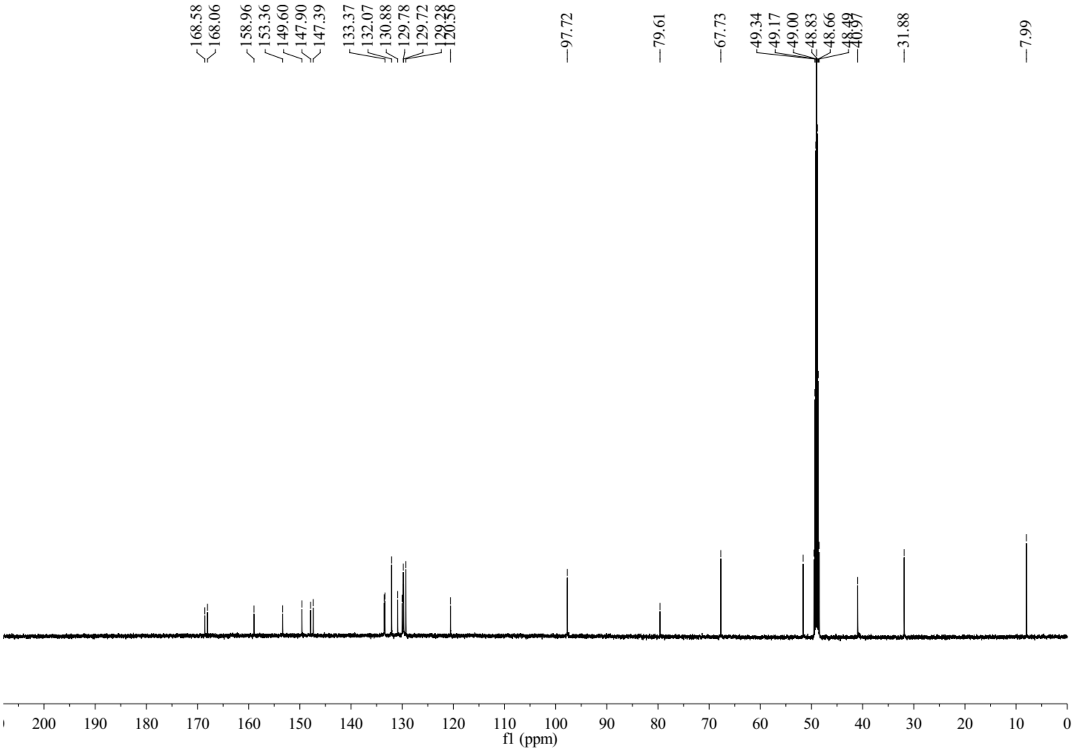

Figure S37: The ^{13}C-NMR of **CPT-B-L2**.

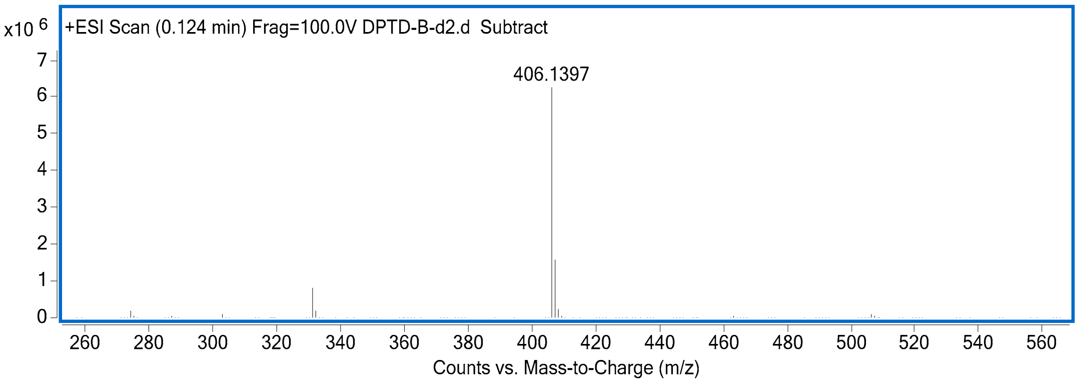

Figure S38: The HRMS of **CPT-B-L2**.

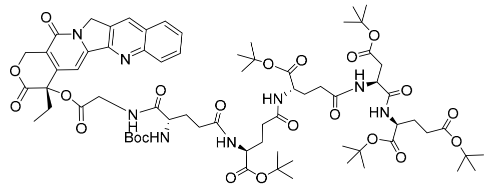

Figure S39: The structure of **CPTD-HT-J-L2**.

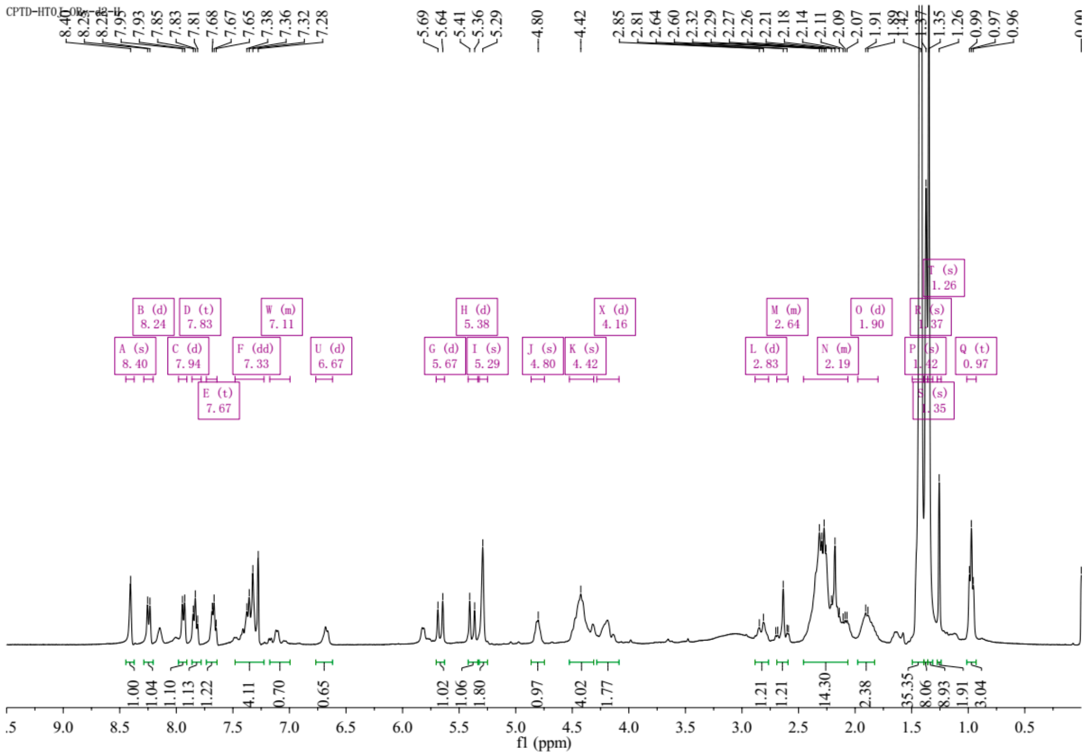

Figure S40: The ^{1}H-NMR of **CPT-HT-J-L2**.

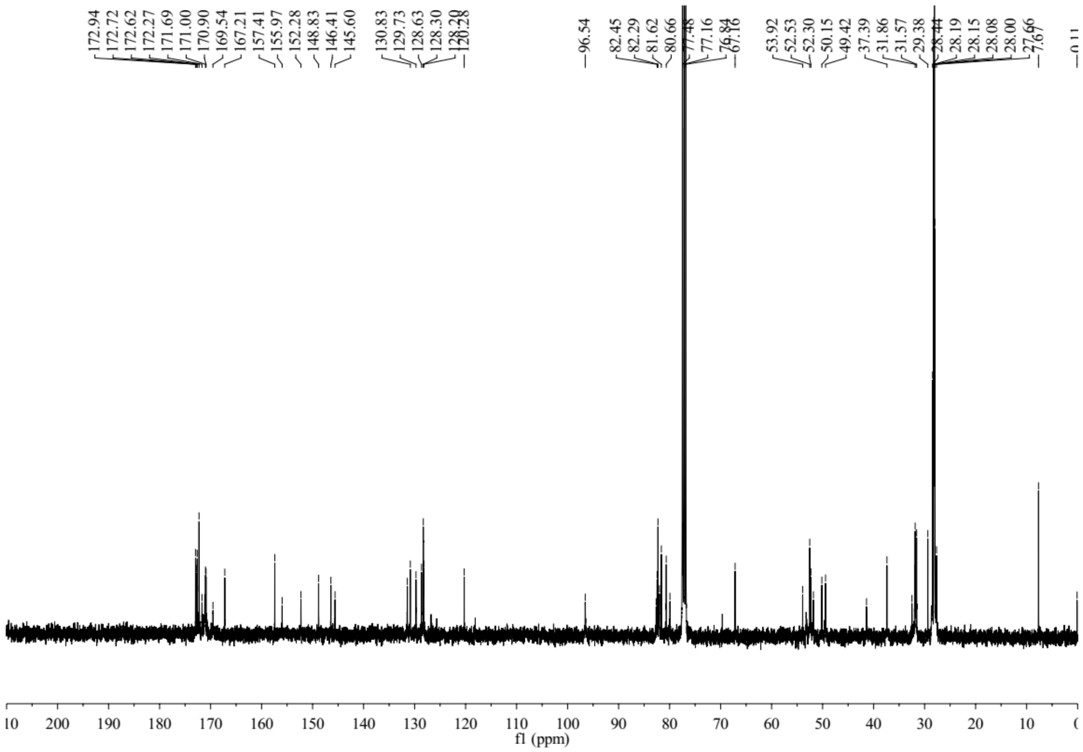

Figure S41: The ^{13}C-NMR of **CPT-HT-J-L2**.

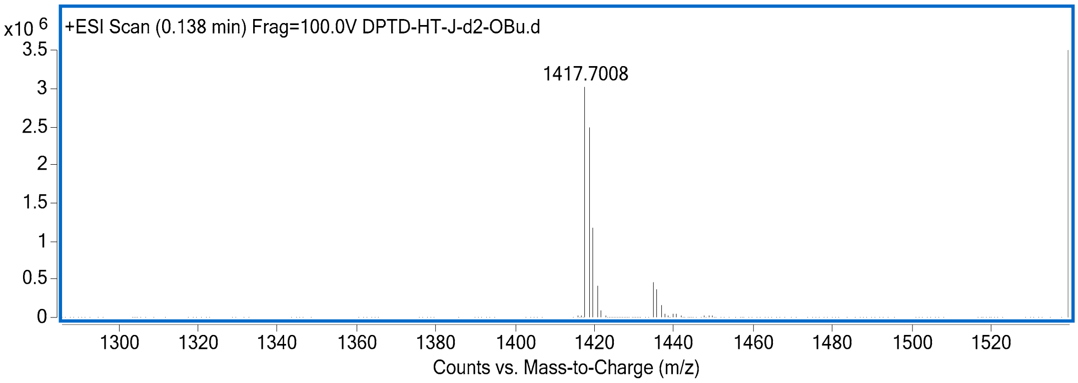

Figure S42: The HRMS of **CPT-HT-J-L2**.

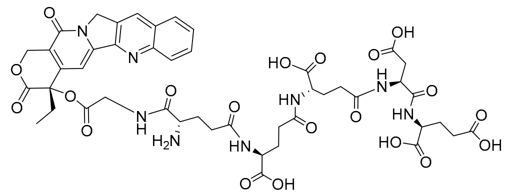

Figure S43: The structure of **CPT-HT-J-ZL2**.

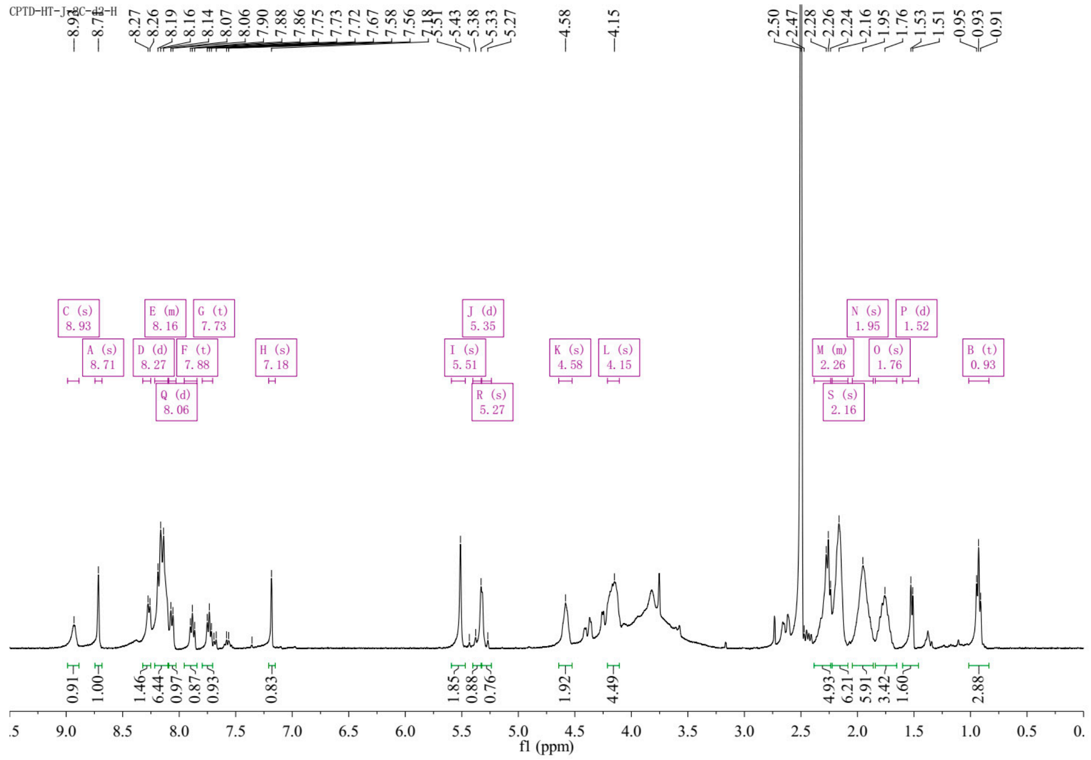

Figure S44: The ^{1}H-NMR of **CPT-HT-J-ZL$_2$**.

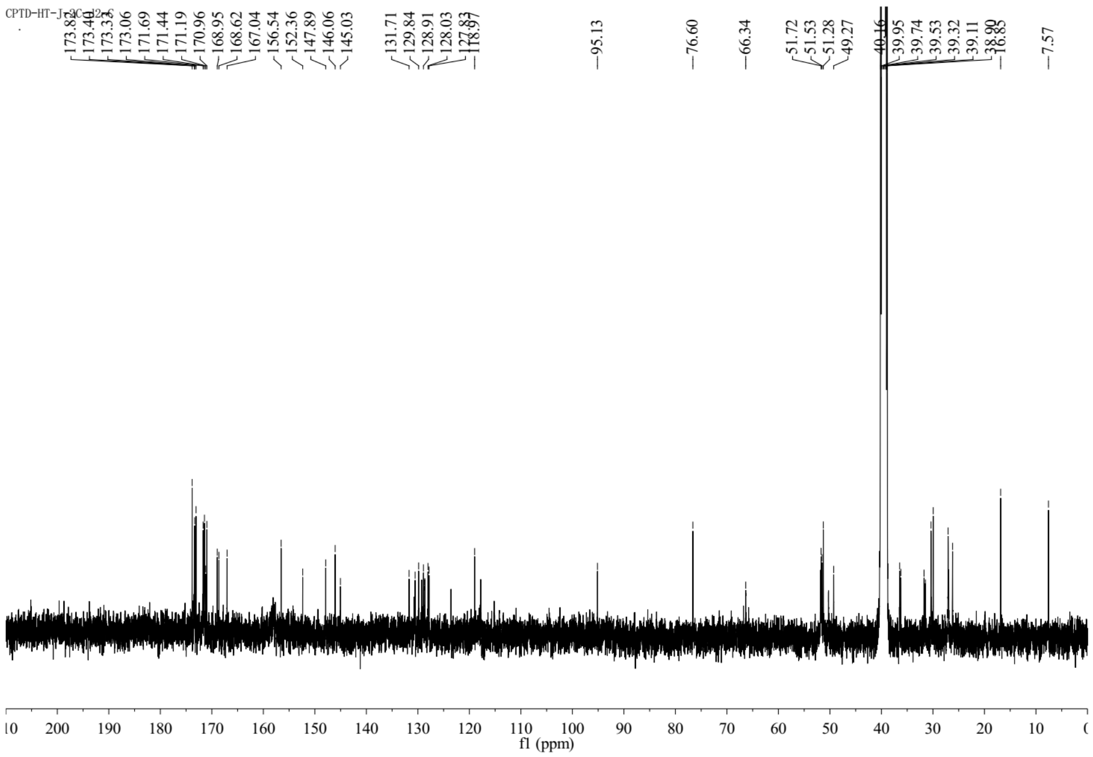

Figure S45: The ^{13}C-NMR of **CPT-HT-J-ZL$_2$**.

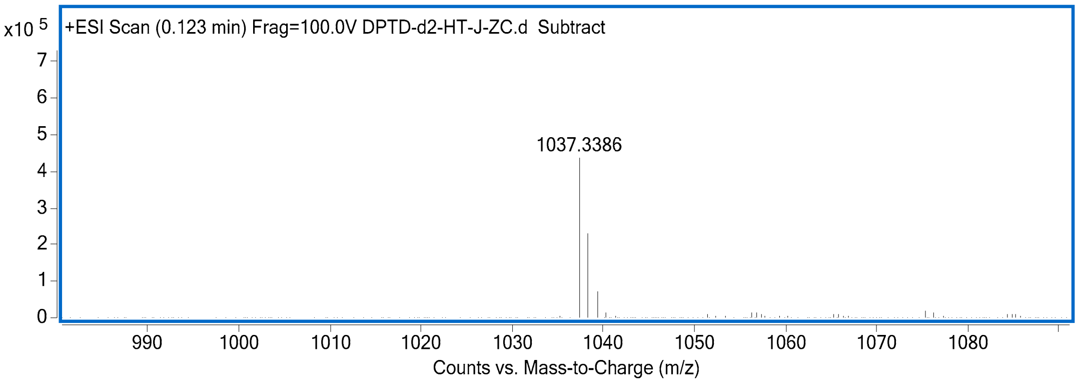

Figure S46: The HRMS of **CPT-HT-J-ZL2**.

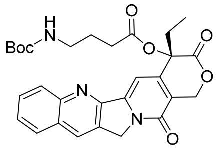

Figure S47: The structure of **CPT-A-L4**.

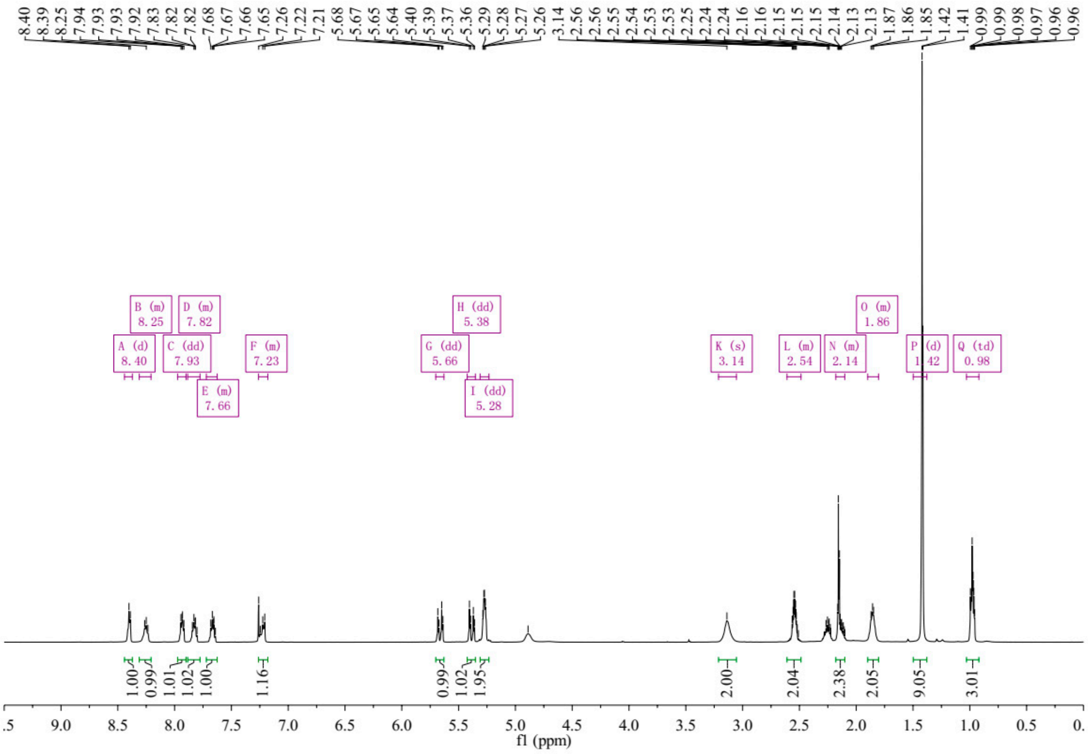

Figure S48: The ^{1}H-NMR of **CPT-A-L4**.

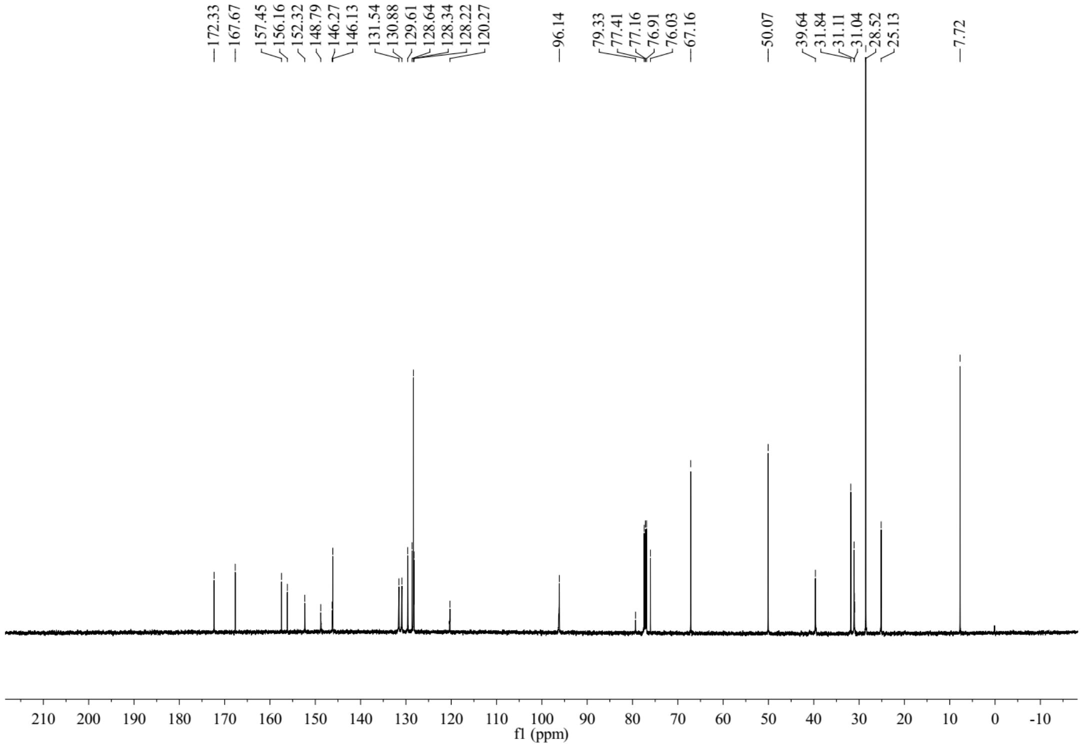

Figure S49: The ^{13}C-NMR of **CPT-A-L4**.

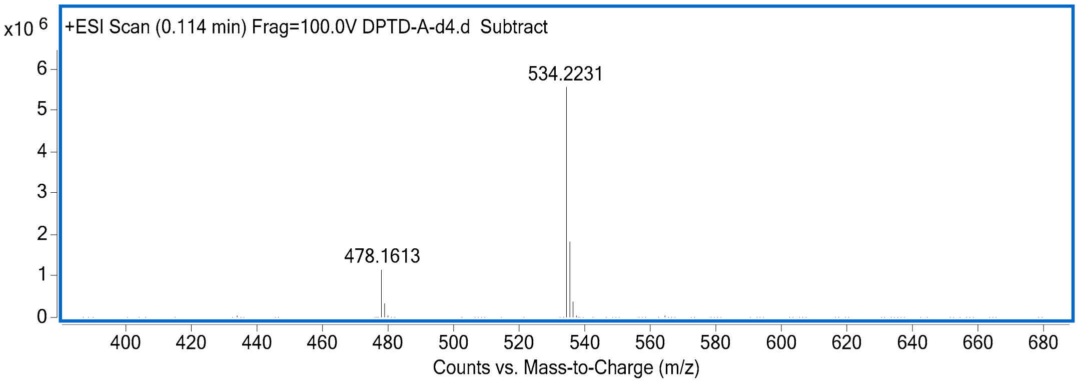

Figure S50: The HRMS of **CPT-A-L4**.

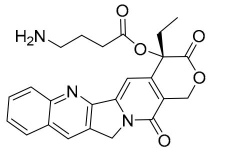

Figure S51: The structure of **CPT-B-L4**.

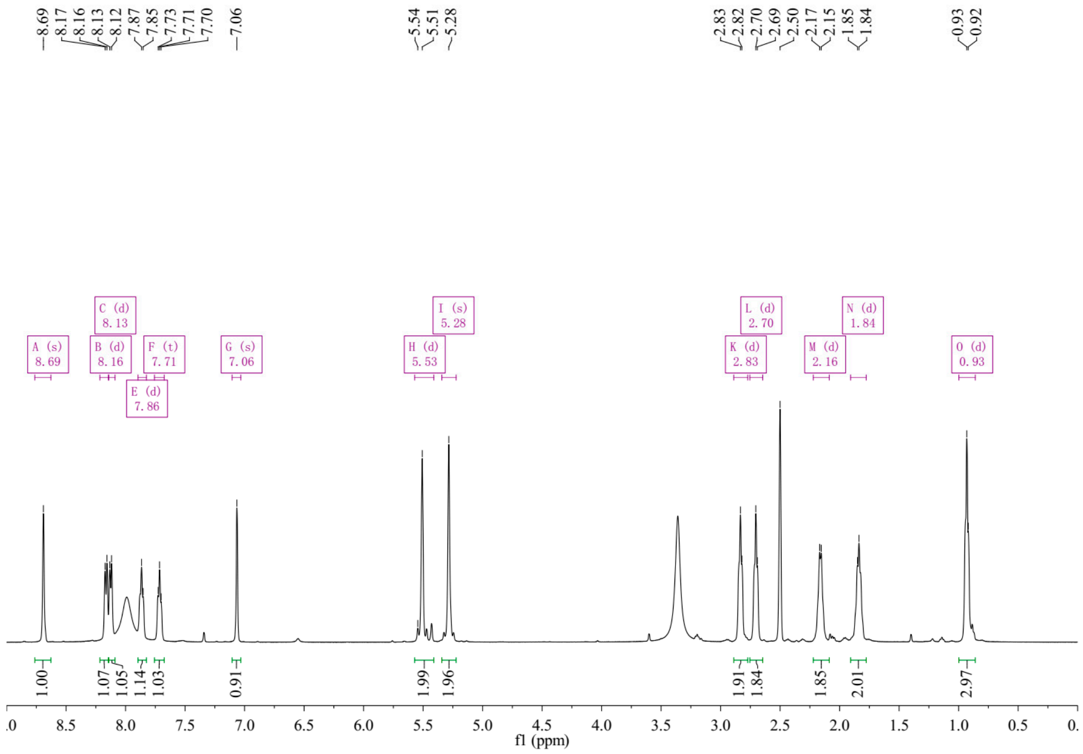

Figure S52: The ^{1}H-NMR of **CPT-B-L₄**.

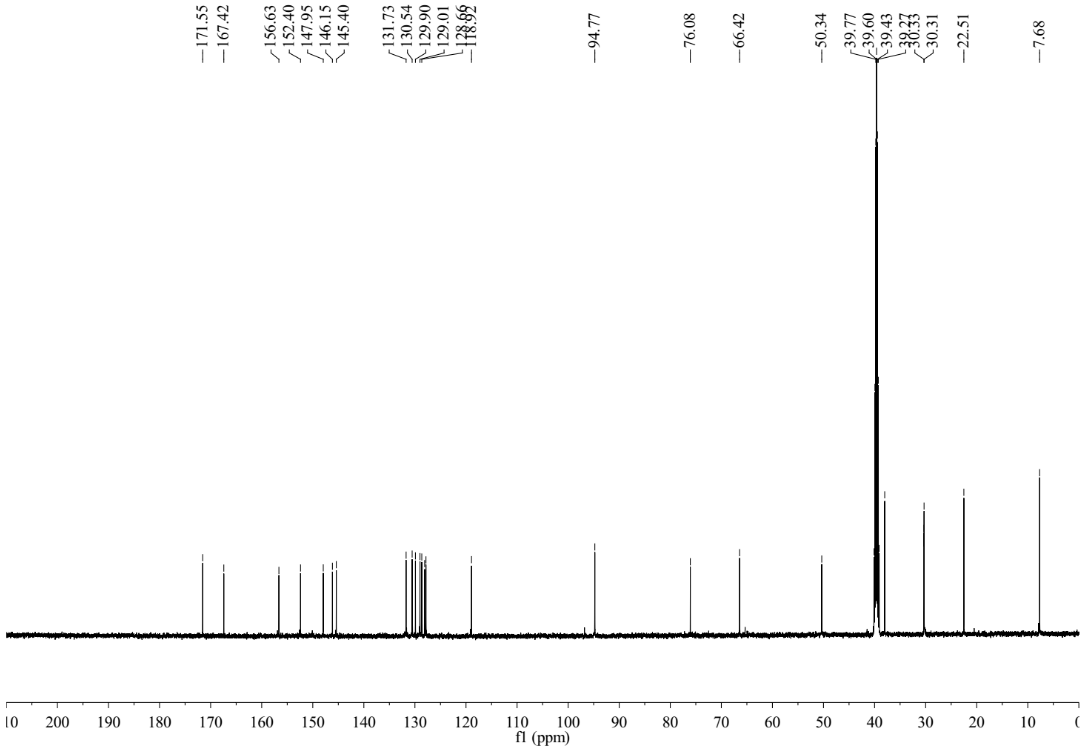

Figure S53: The ^{13}C-NMR of **CPT-B-L₄**.

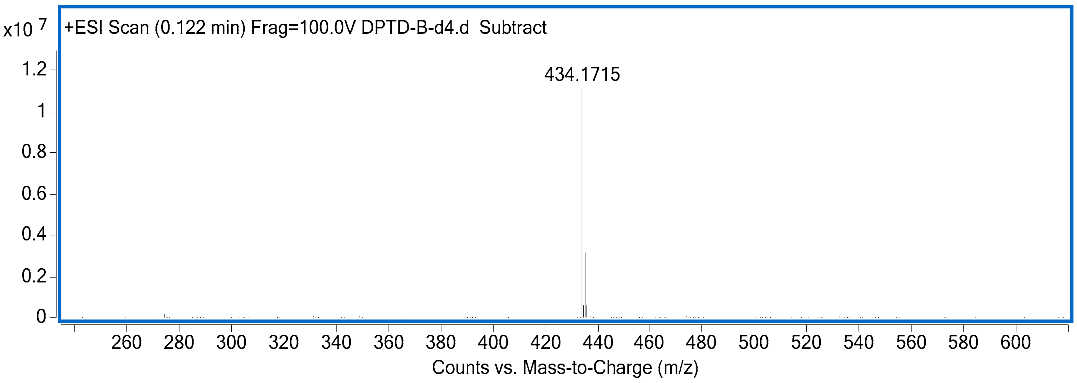

Figure S54: The HRMS of **CPT-B-L4**.

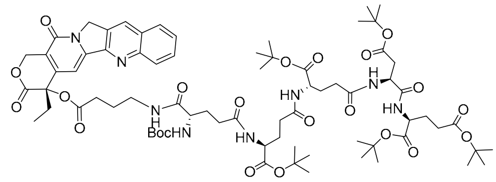

Figure S55: The structure of **CPT-HT-J-L4**.

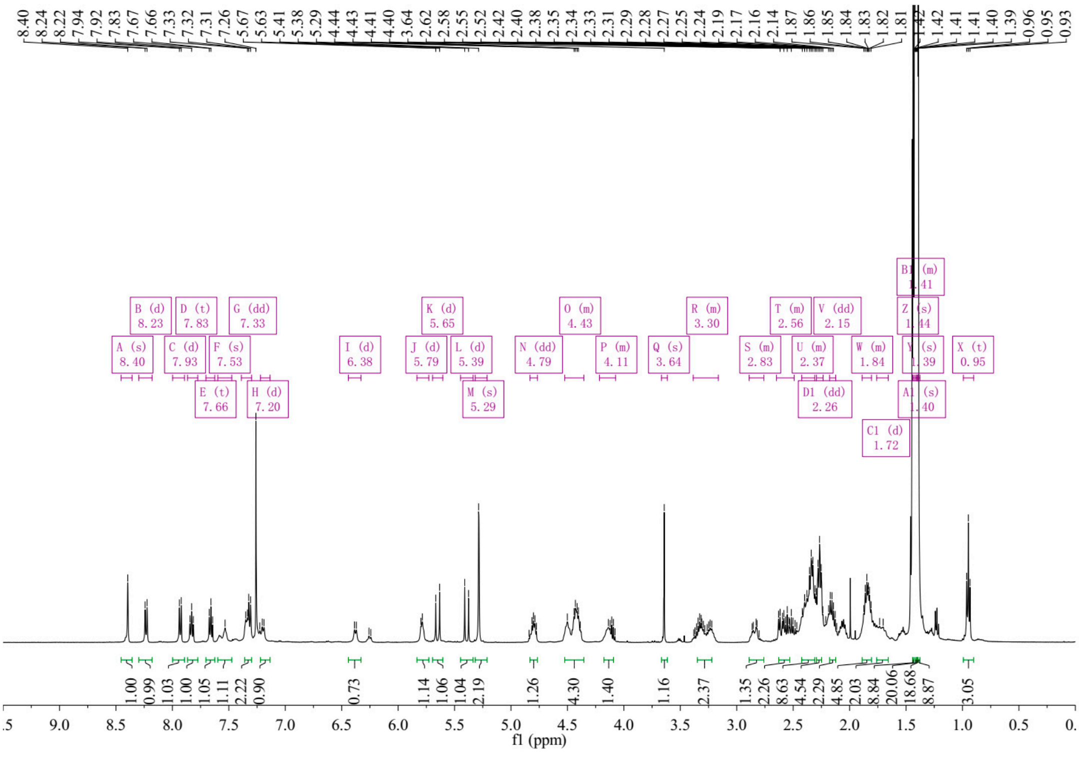

Figure S56: The ^{1}H-NMR of **CPT-HT-J-L4**.

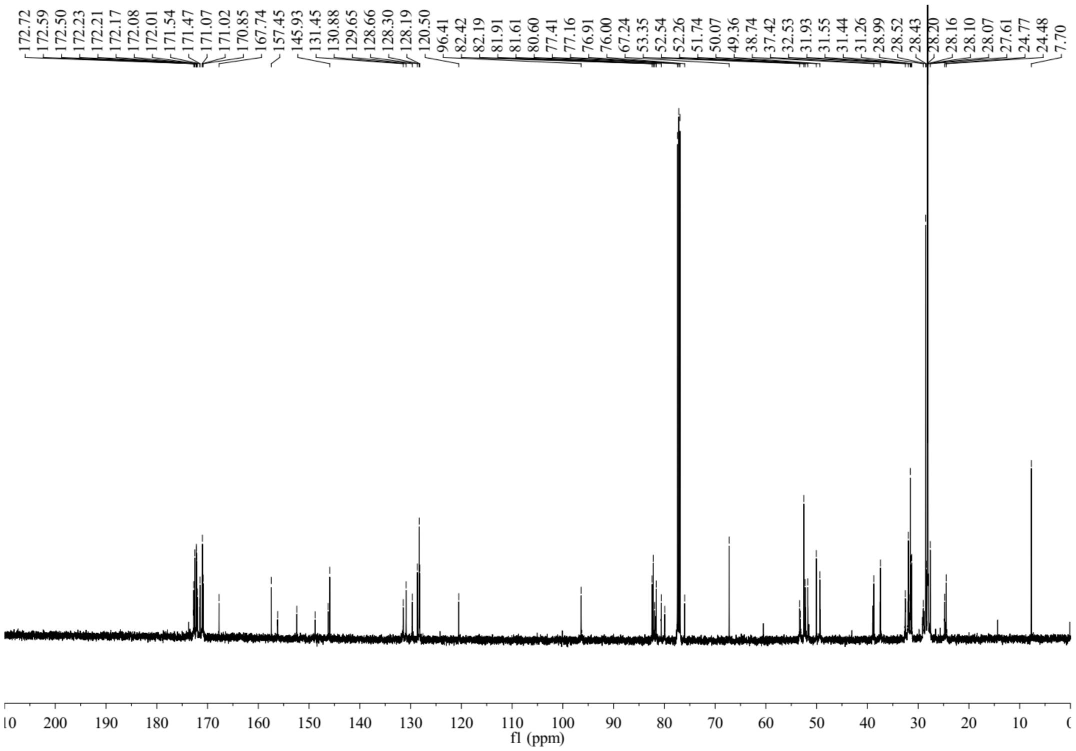

Figure S57: The ^{13}C-NMR of **CPT-HT-J-L$_4$**.

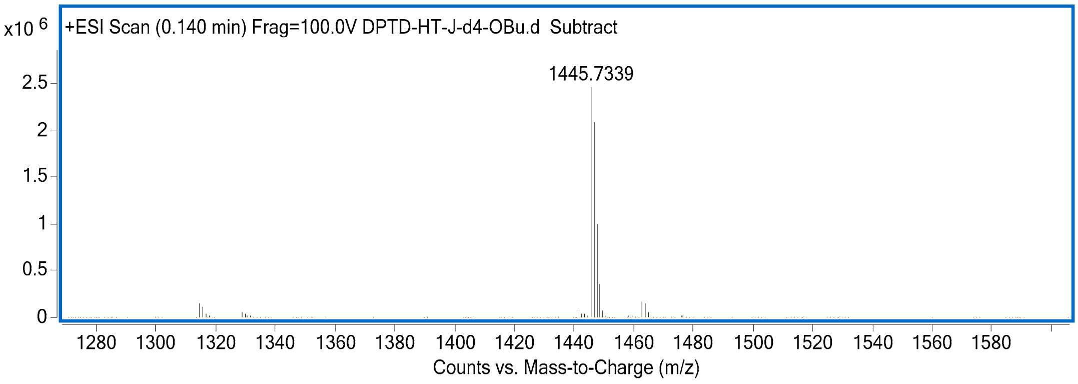

Figure S58: The HRMS of **CPT-HT-J-L$_4$**.

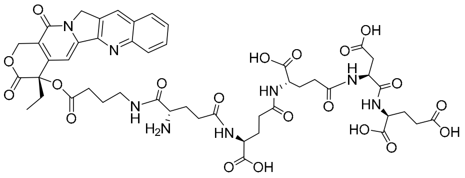

Figure S59: The structure of **CPT-HT-J-ZL$_4$**.

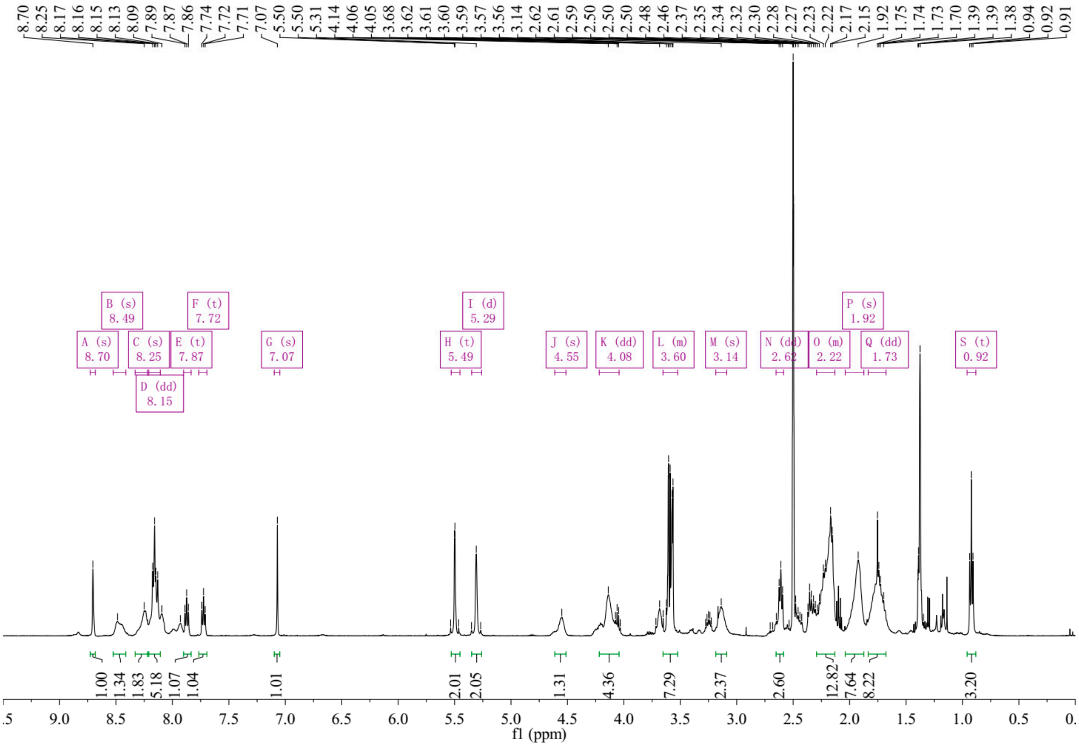

Figure S60: The ^{1}H-NMR of **CPT-HT-J-ZL4**.

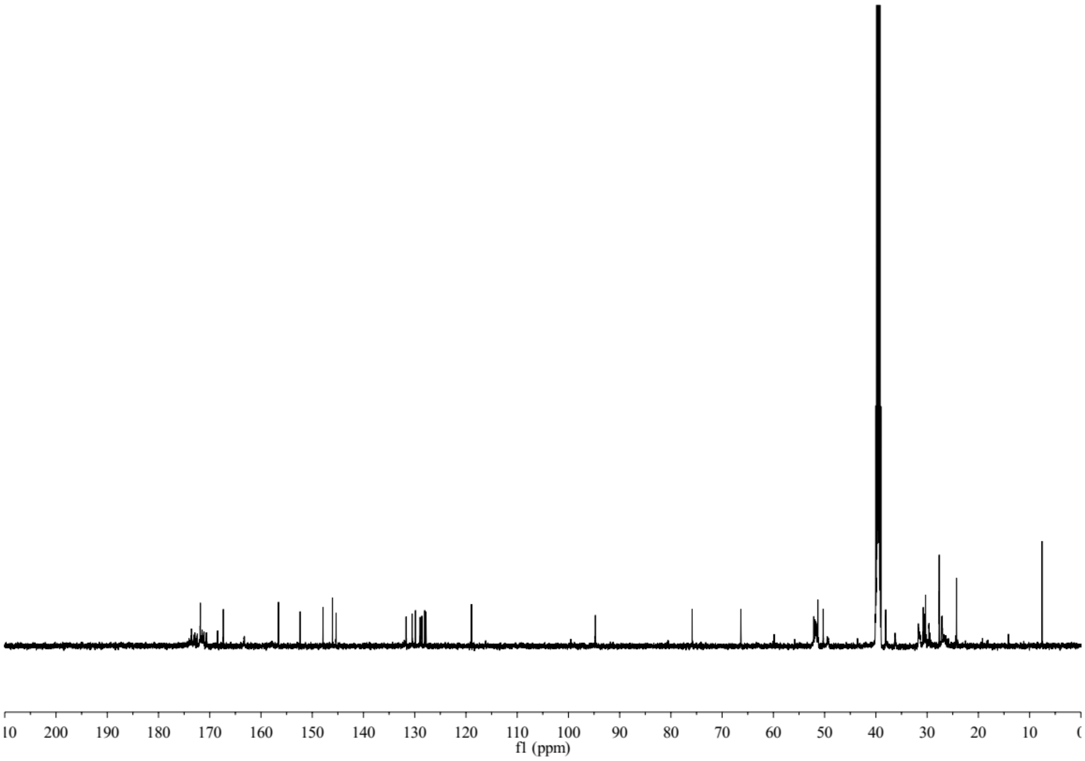

Figure S61: The ^{13}C-NMR of **CPT-HT-J-ZL4**.

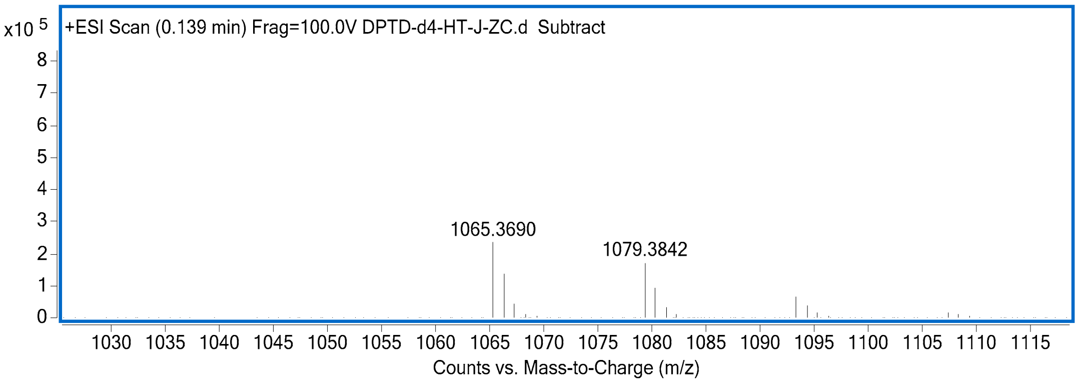

Figure S62: The HRMS of **CPT-HT-J-ZL4**.

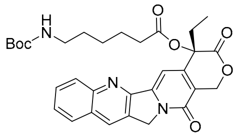

Figure S63: The structure of **CPT-A-L6**.

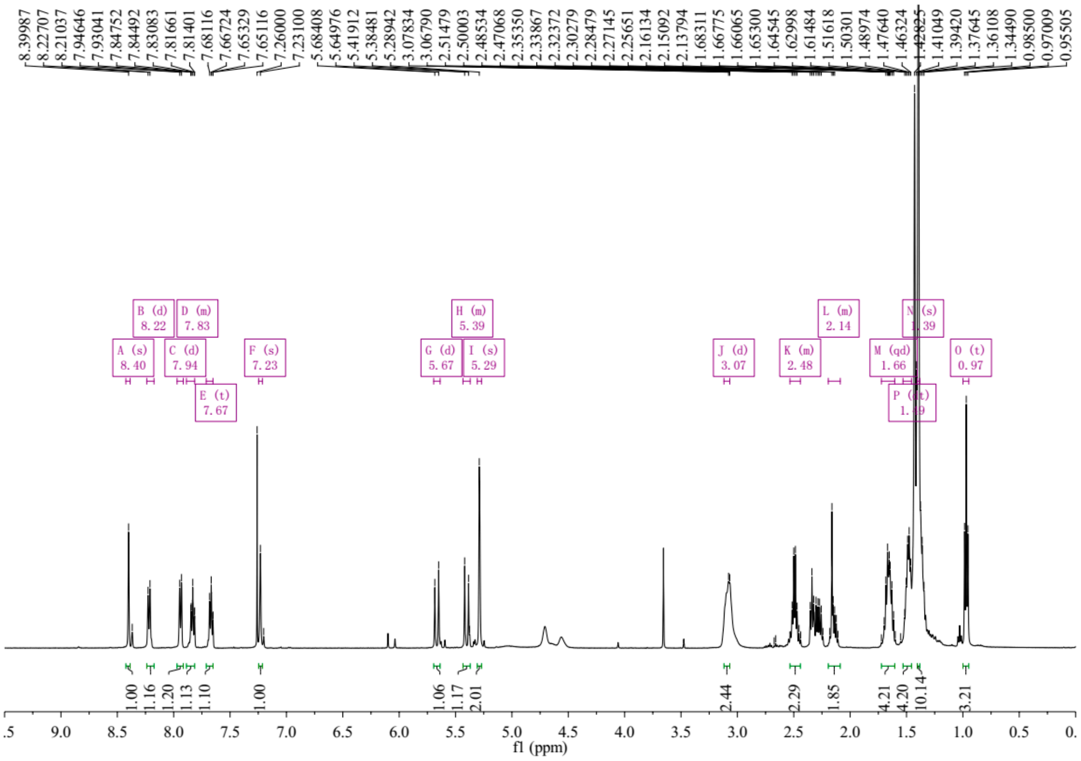

Figure S64: The ^{1}H-NMR of **CPT-A-L6**.

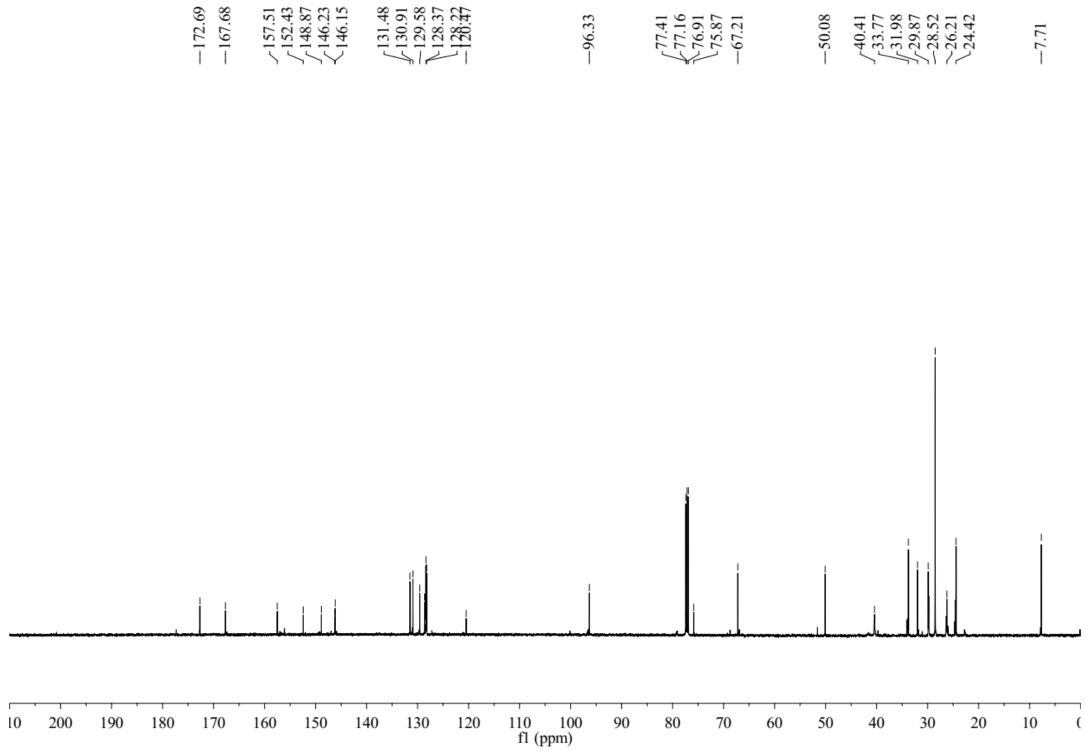

Figure S65: The ^{13}C-NMR of **CPT-A-L$_6$**.

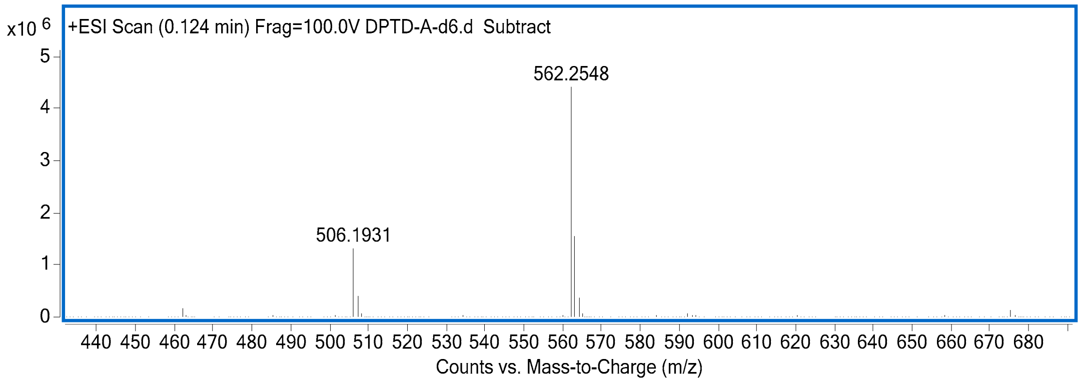

Figure S66: The HRMS of **CPT-A-L$_6$**.

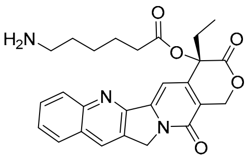

Figure S67: The structure of **CPT-B-L$_6$**.

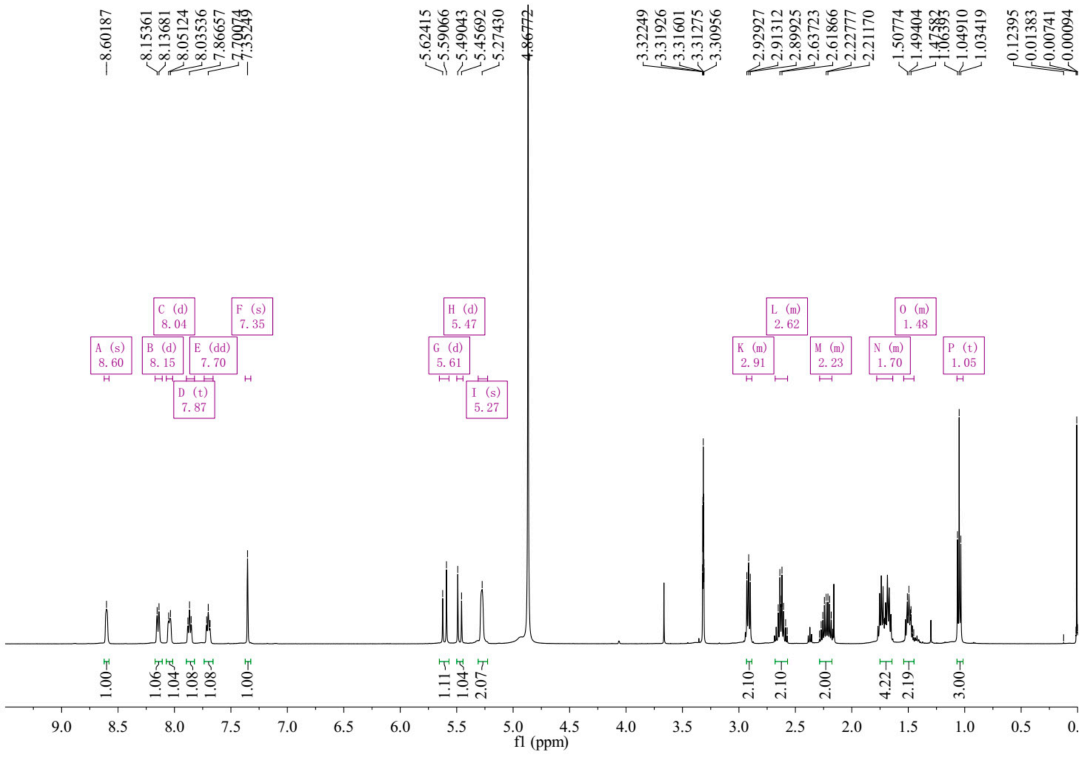

Figure S68: The ^{1}H-NMR of **CPT-B-L$_6$**.

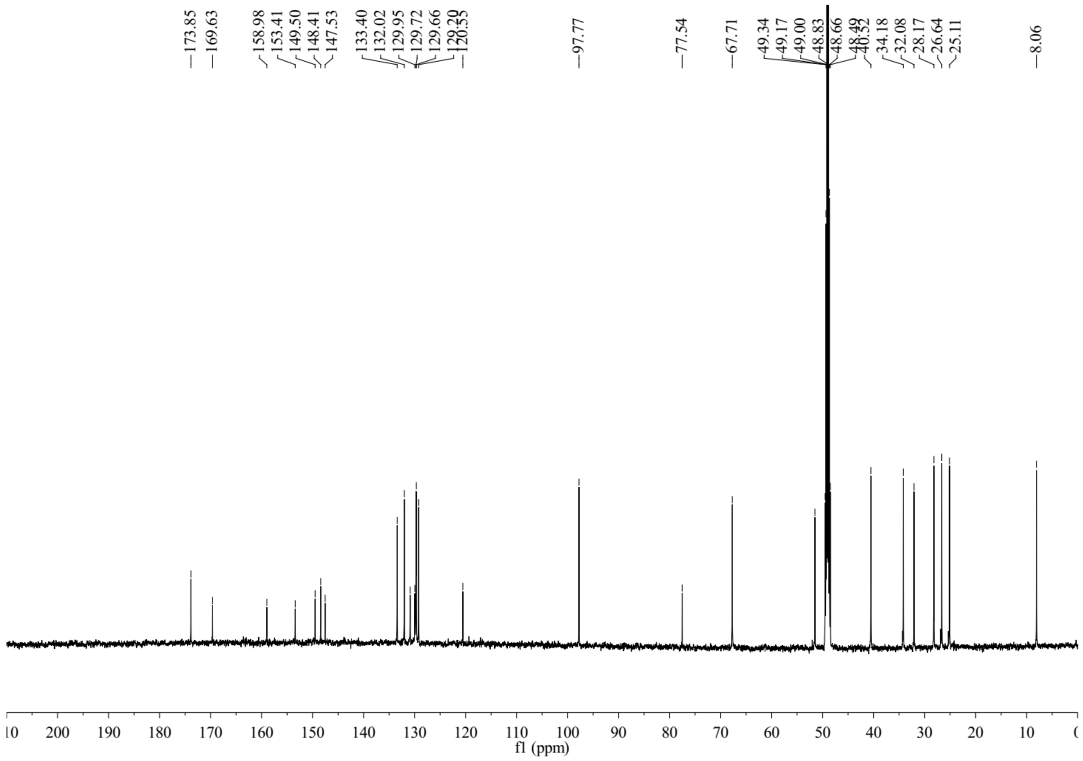

Figure S69: The ^{13}C-NMR of **CPT-B-L$_6$**.

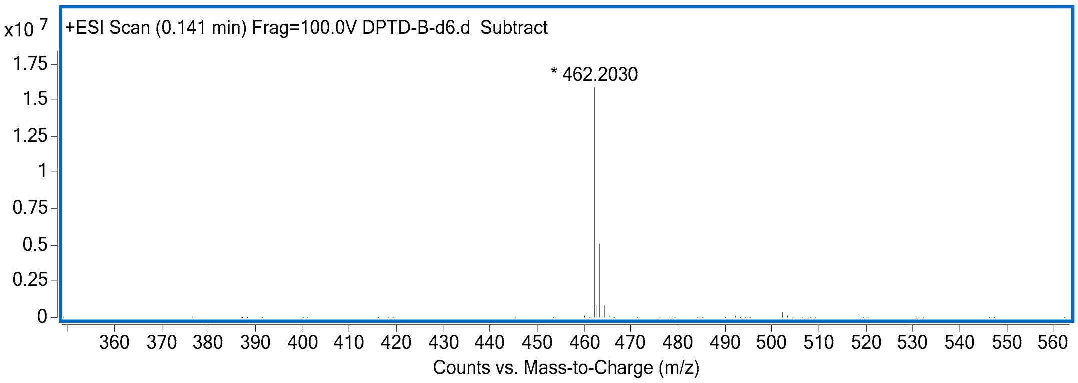

Figure S70: The HRMS of **CPT-B-L₆**.

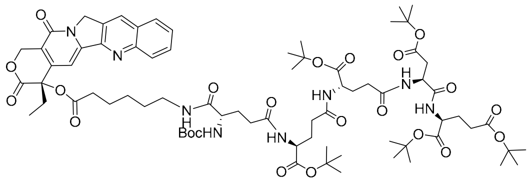

Figure S71: The structure of **CPT-HT-J-L₆**.

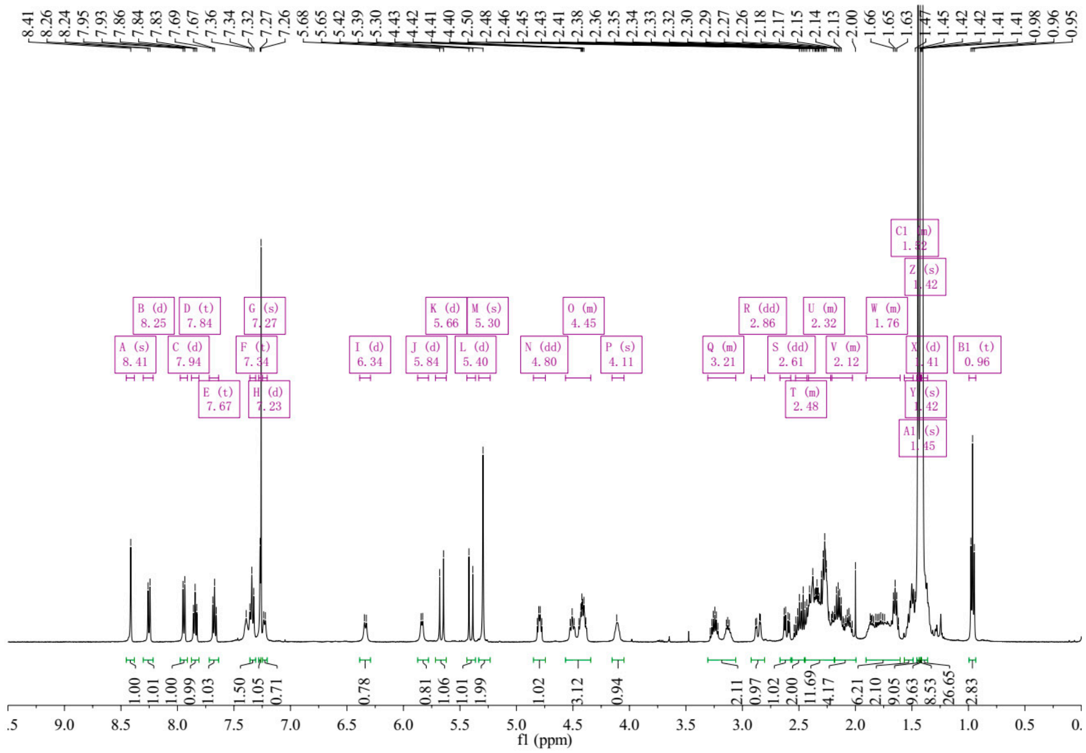

Figure S72: The ^{1}H-NMR of **CPT-HT-J-L₆**.

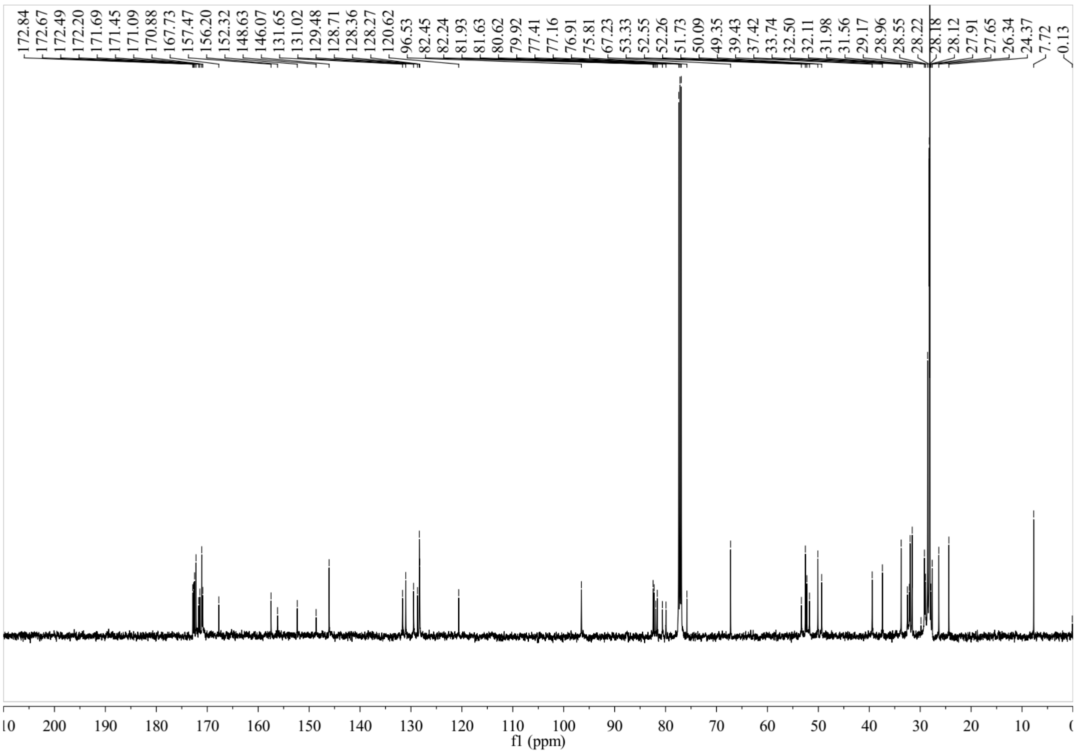

Figure S73: The ^{13}C-NMR of **CPT-HT-J-L6**.

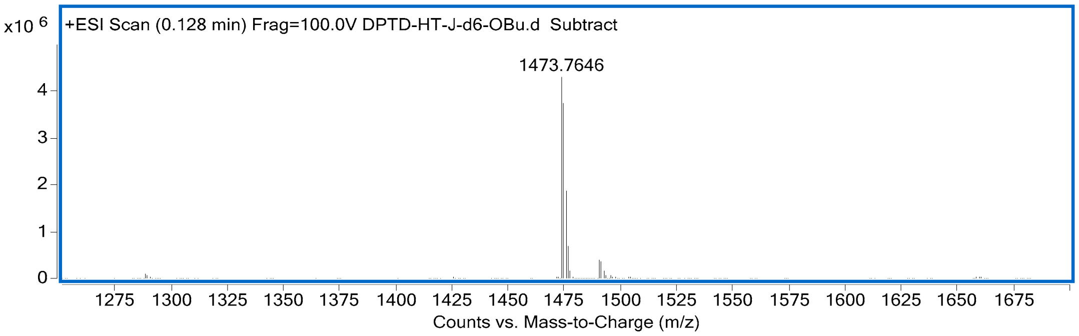

Figure S74: The HRMS of **CPT-HT-J-L6**.

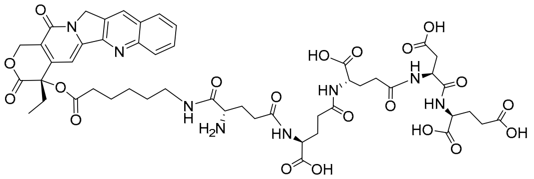

Figure S75: The structure of **CPT-HT-J-ZL6**.

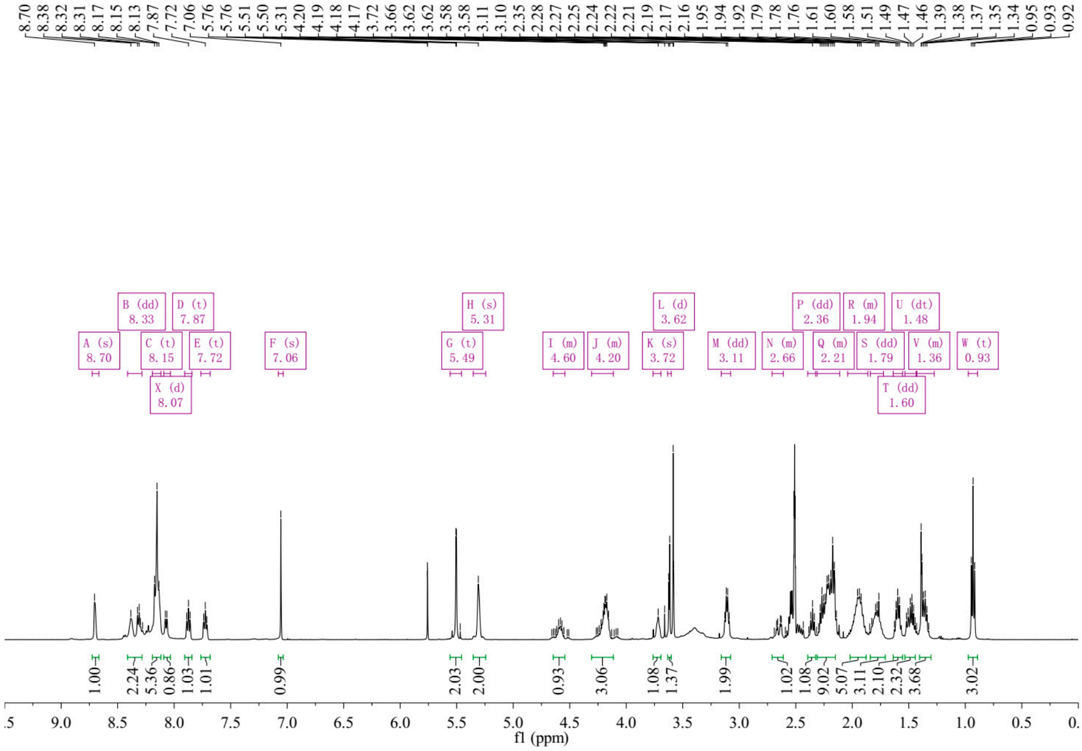

Figure S76: The ^{1}H-NMR of **CPT-HT-J-ZL$_6$**.

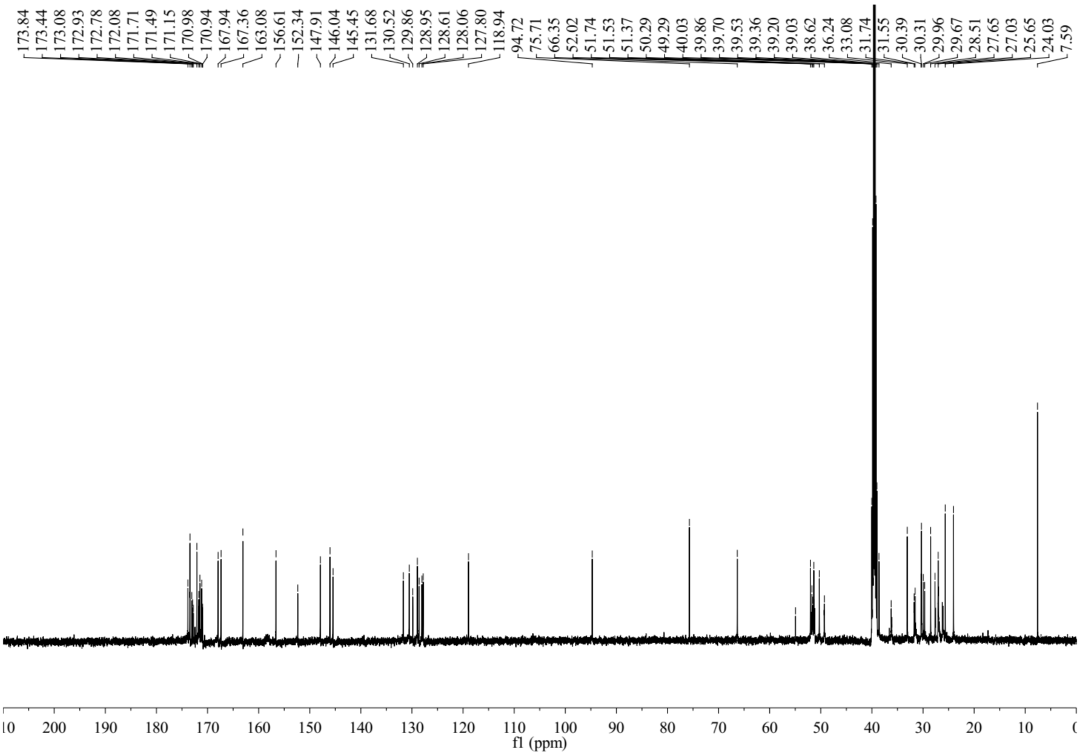

Figure S77: The ^{13}C-NMR of **CPT-HT-J-ZL$_6$**.

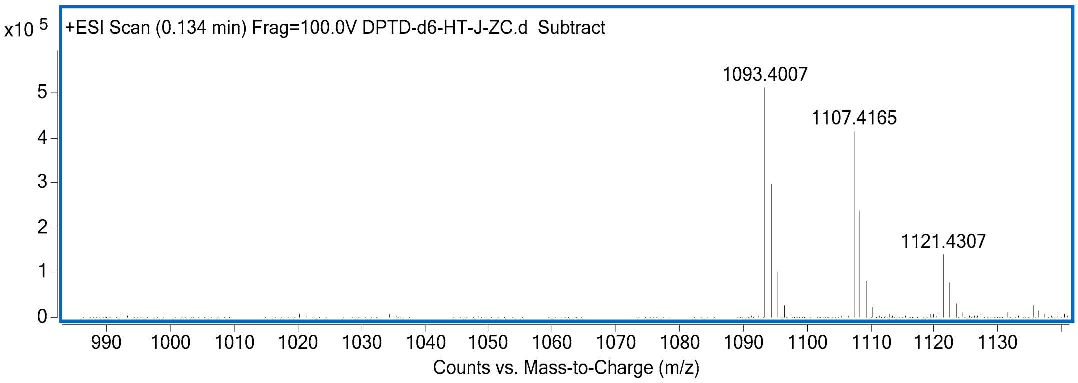

Figure78: The HRMS of **CPT-HT-J-ZL6**.

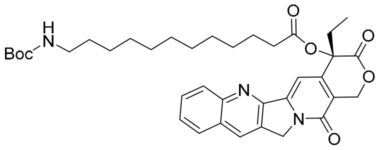

Figure S79: The structure of **CPT-A-L12**.

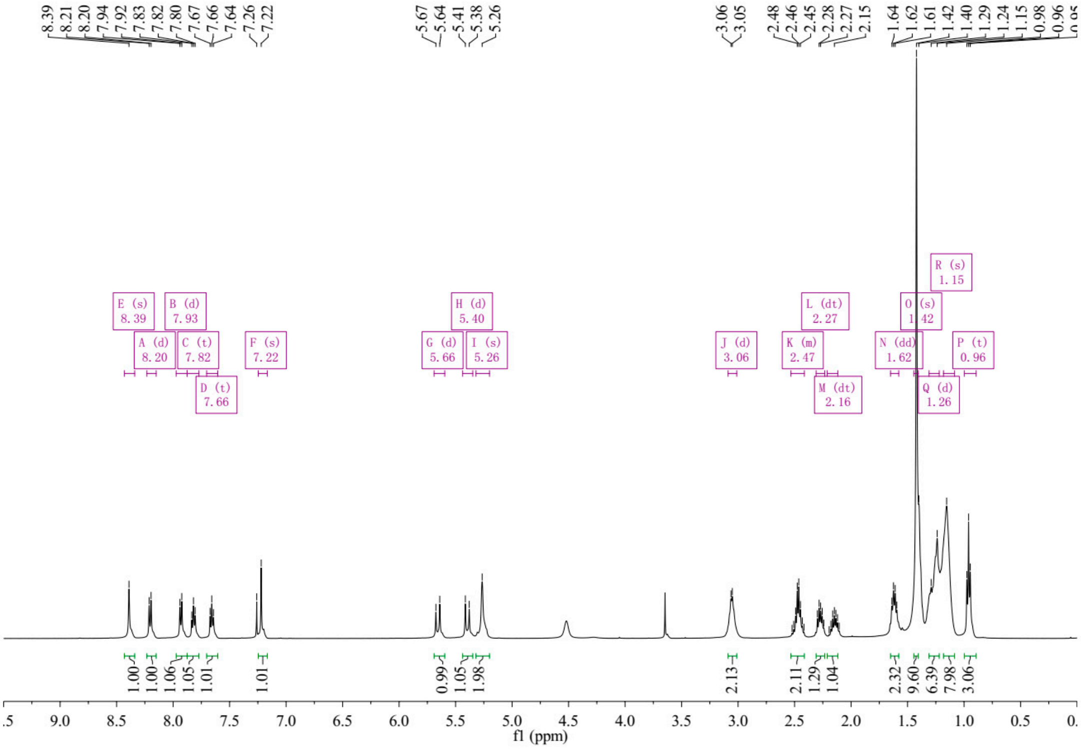

Figure S80: The ^{1}H-NMR of **CPT-A-L12**.

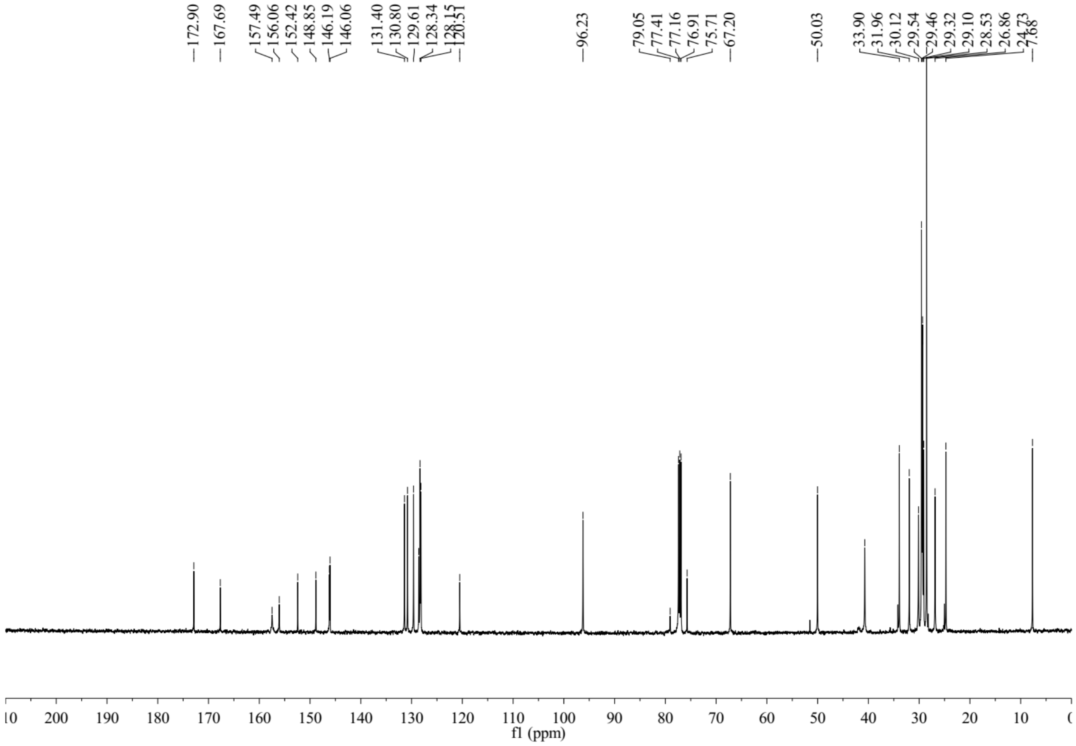

Figure S81: The ^{13}C-NMR of **CPT-A-L12**.

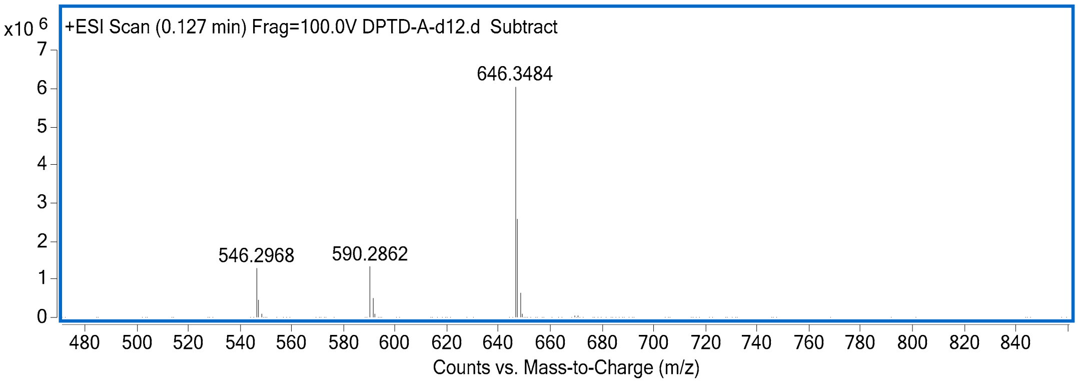

Figure S82: The HRMS of **CPT-A-L12**.

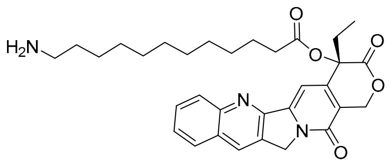

Figure S83: The structure of **CPT-B-L12**.

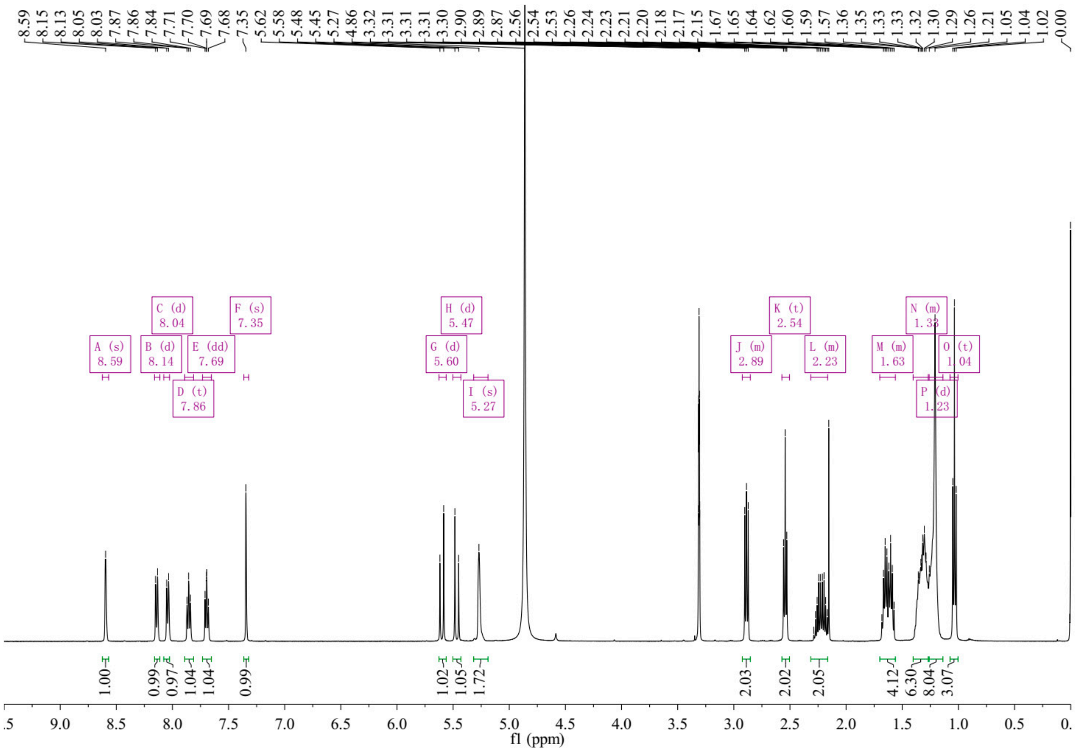

Figure S84: The ^{1}H-NMR of **CPT-B-L**12.

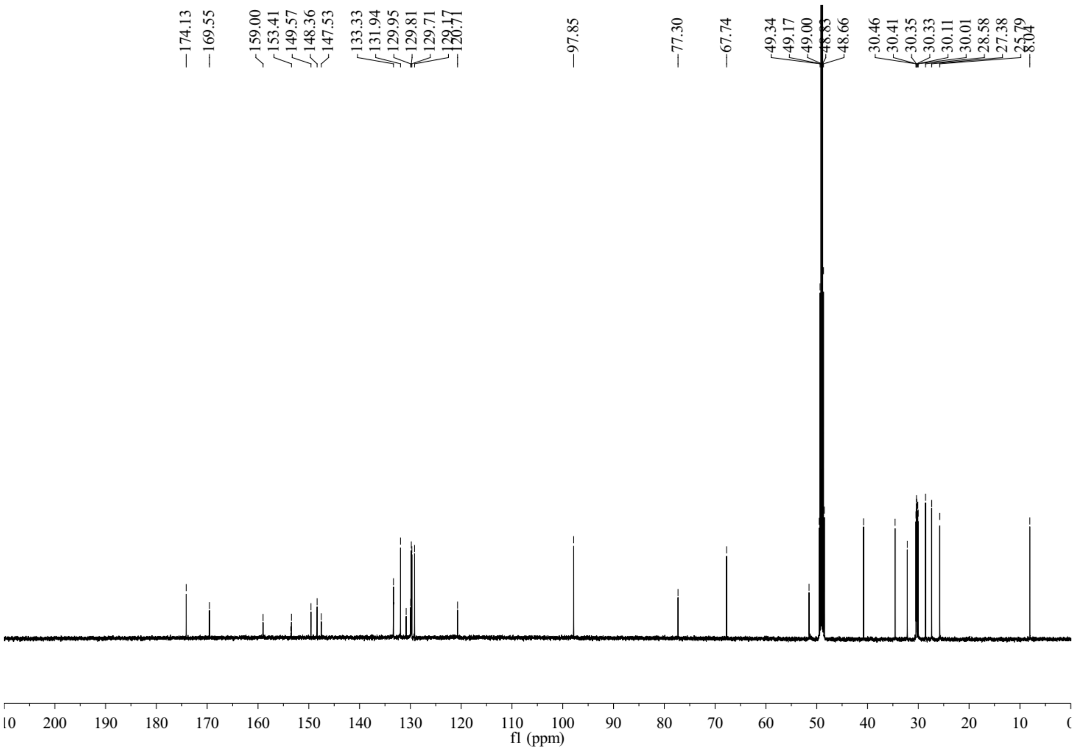

Figure S85: The ^{13}C-NMR of **CPT-B-L**12.

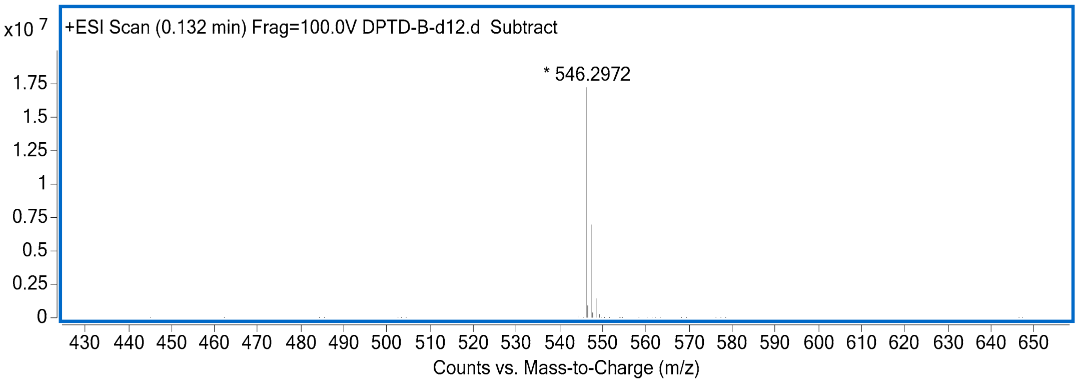

Figure S86: The HRMS of **CPT-B-L12**.

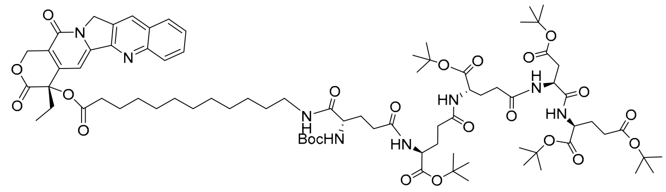

Figure S87: The structure of **CPT-HT-J-L12**.

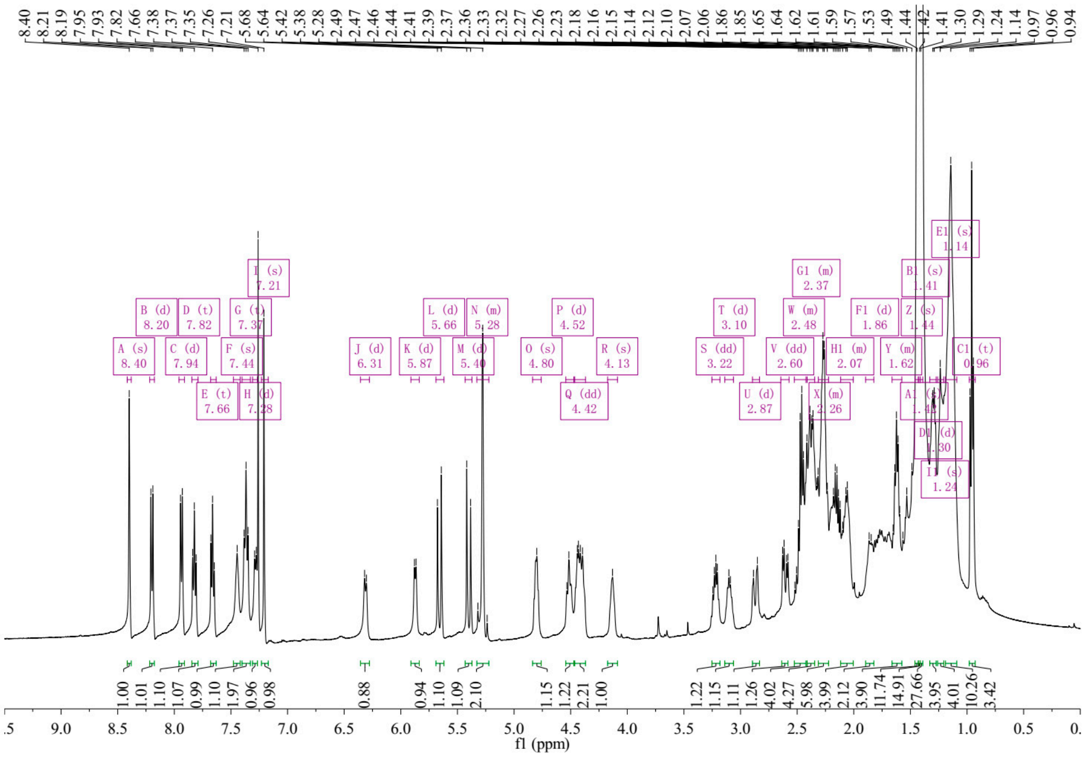

Figure S88: The ^{1}H-NMR of **CPT-HT-J-L12**.

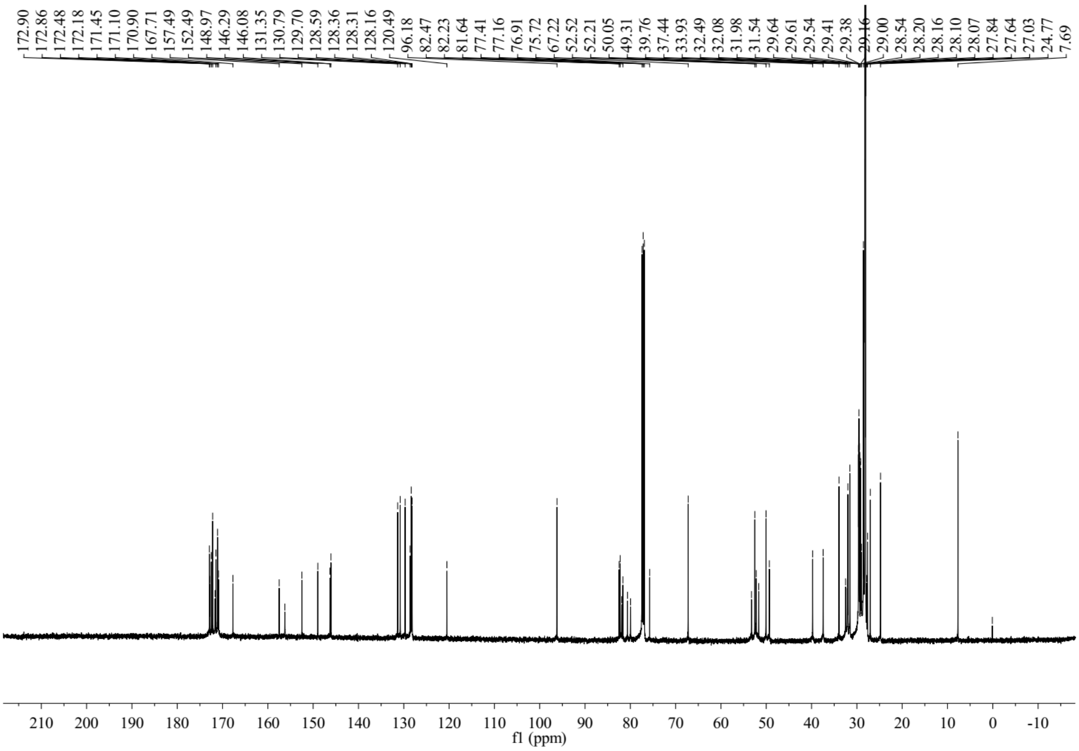

Figure S89: The ^{13}C-NMR of **CPT-HT-J-ZL$_{12}$**.

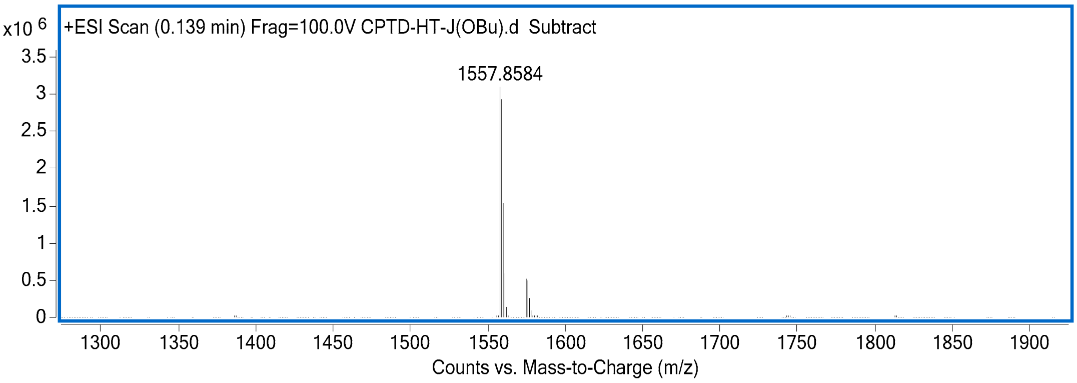

Figure S90: The HRMS of **CPT-HT-J-L$_{12}$**.

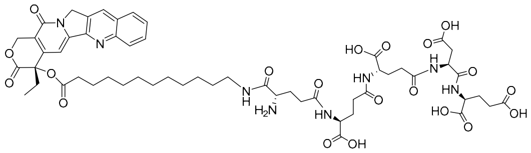

Figure S91: The structure of **CPT-HT-J-ZL$_{12}$**.

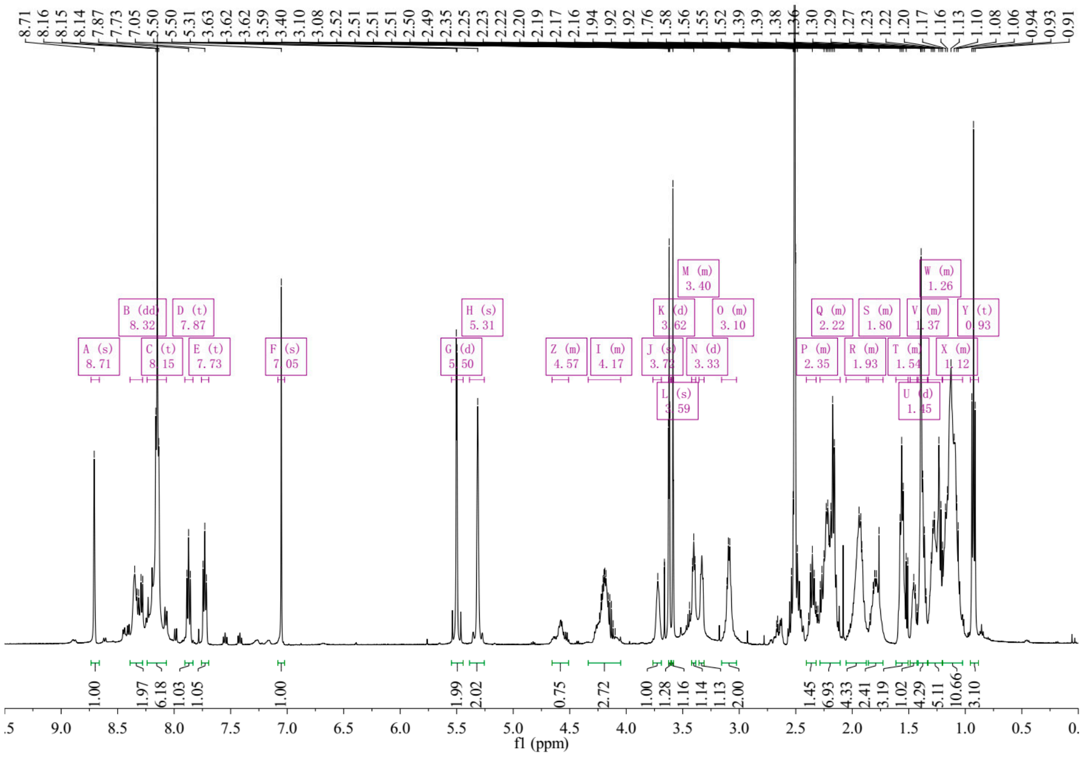

Figure S92: The ^{1}H-NMR of **CPT-HT-J-ZL12**.

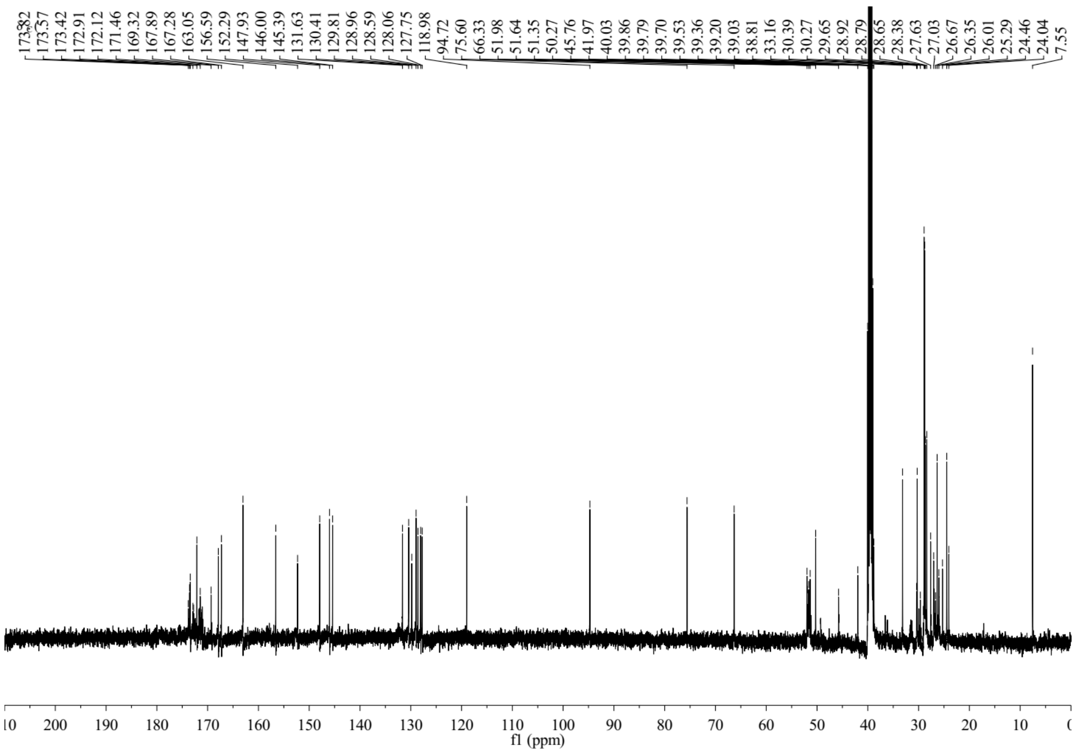

Figure S93: The ^{13}C-NMR of **CPT-HT-J-ZL12**.

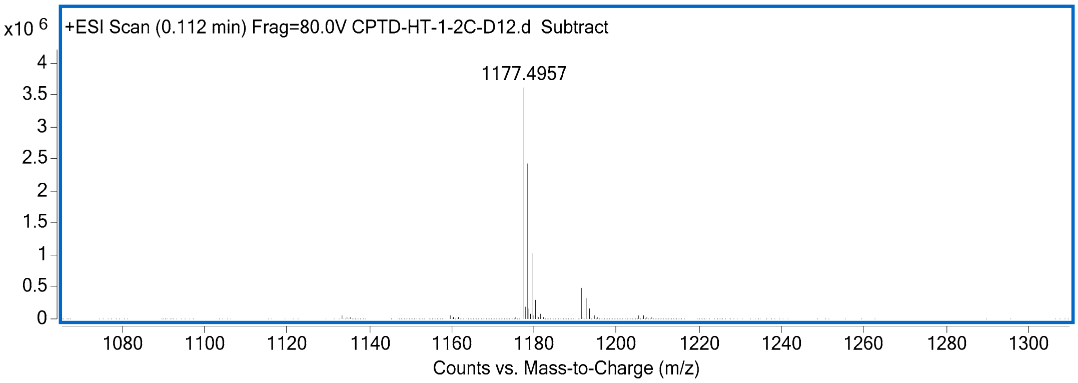

Figure S94: The HRMS of **CPT-HT-J-ZL12**.

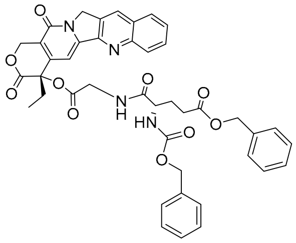

Figure S95: The structure of **CPT-C-GL2**.

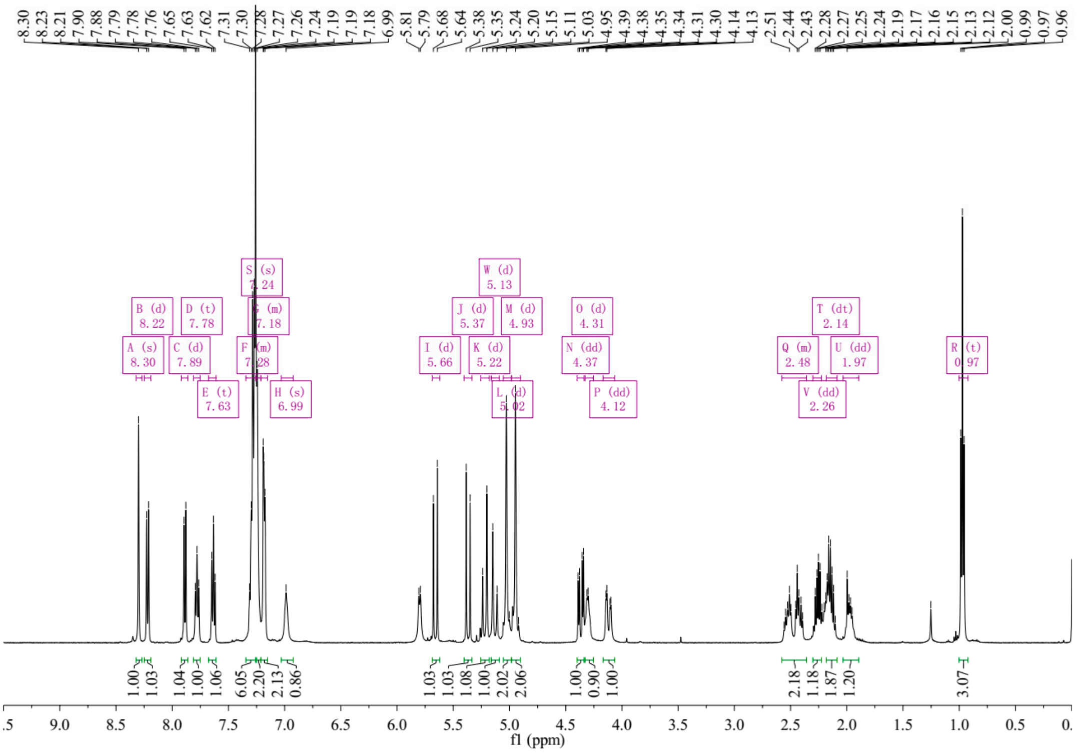

Figure S96: The ^{1}H-NMR of **CPT-C-GL2**.

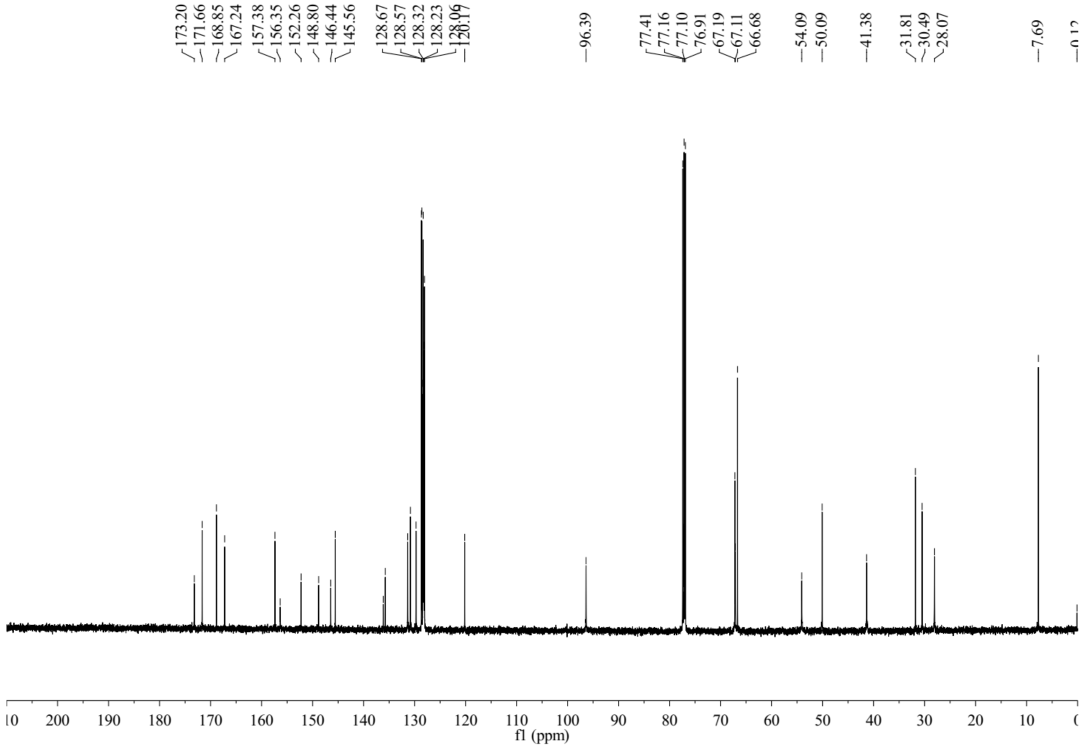

Figure S97: The ^{13}C-NMR of **CPT-C-GL2**.

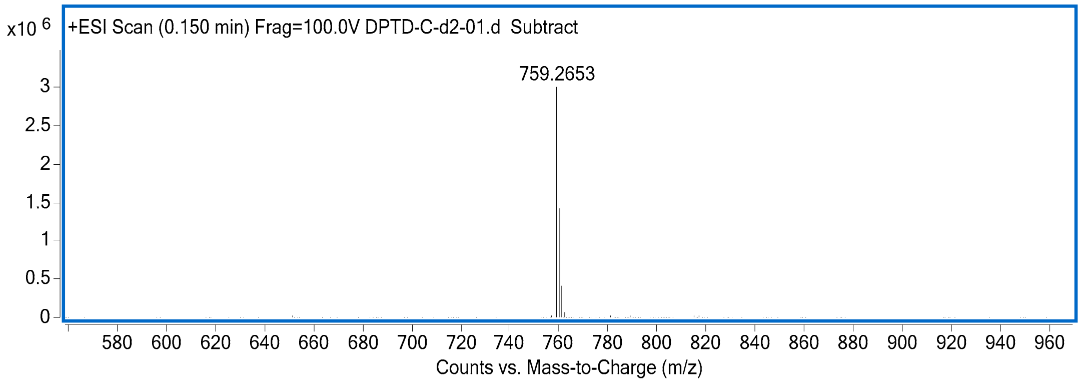

Figure S98: The HRMS of **CPT-C-GL2**.

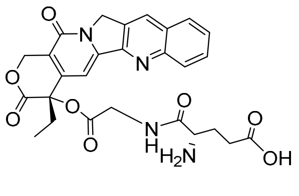

Figure S99: The structure of **CPT-D-GL2**.

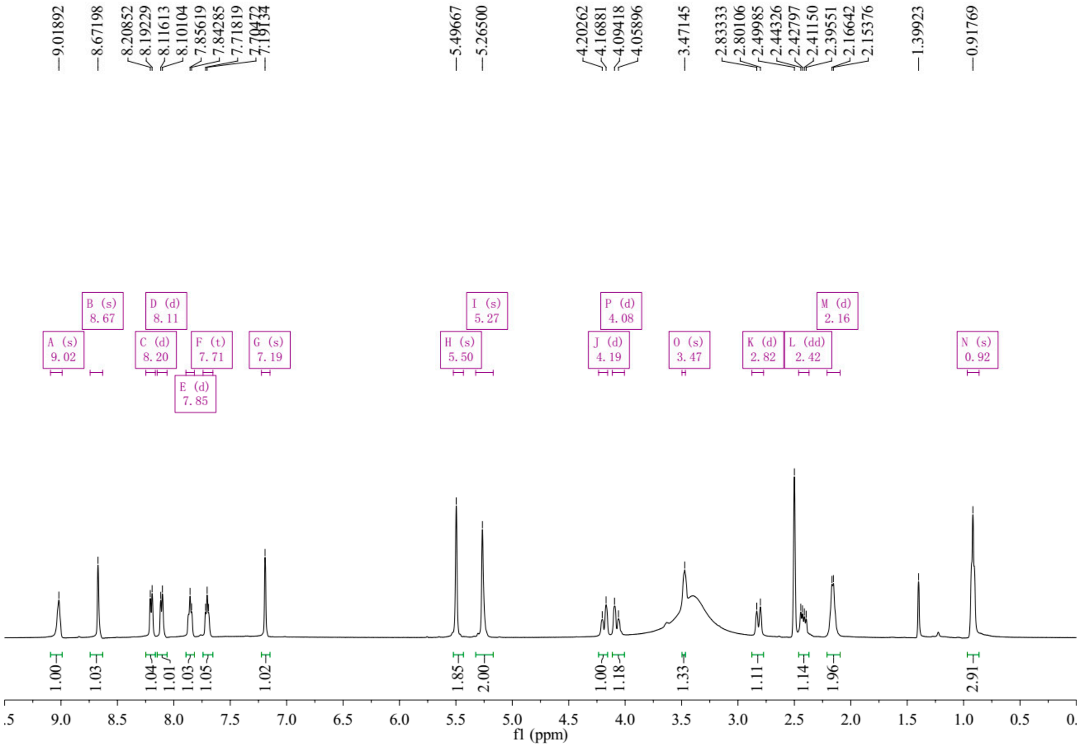

Figure S100: The ^{1}H-NMR of **CPT-D-GL₂**.

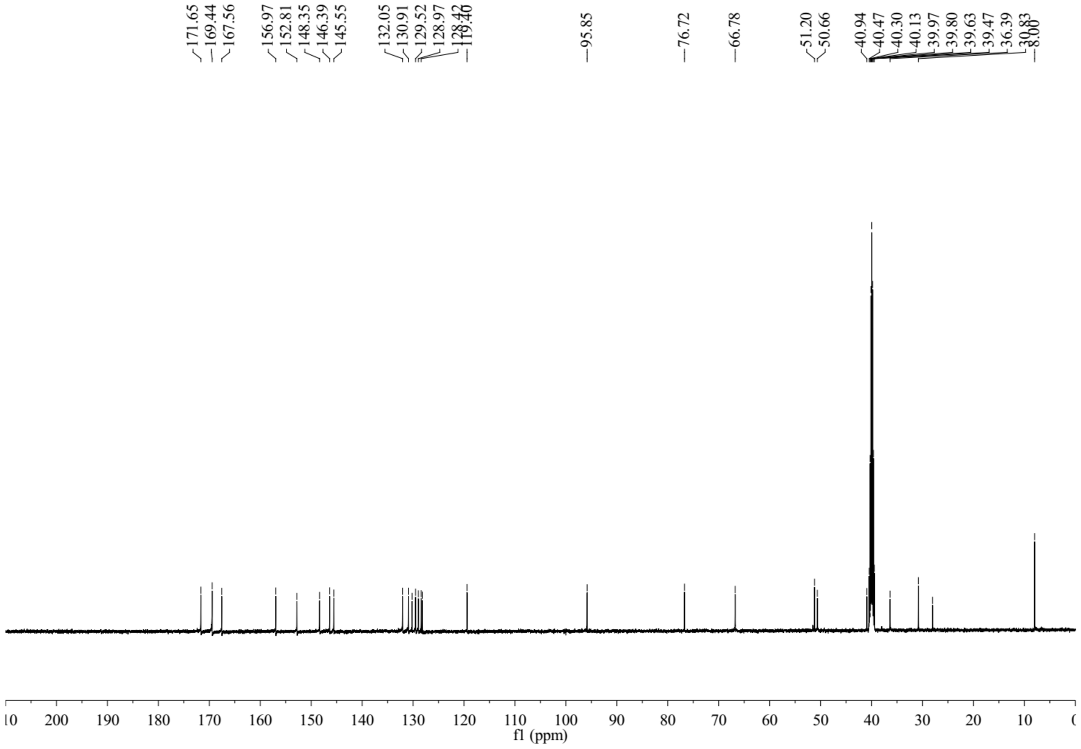

Figure S101: The ^{13}C-NMR of **CPT-D-GL₂**.

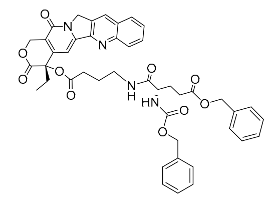

Figure S102: The structure of **CPT-C-GL4**.

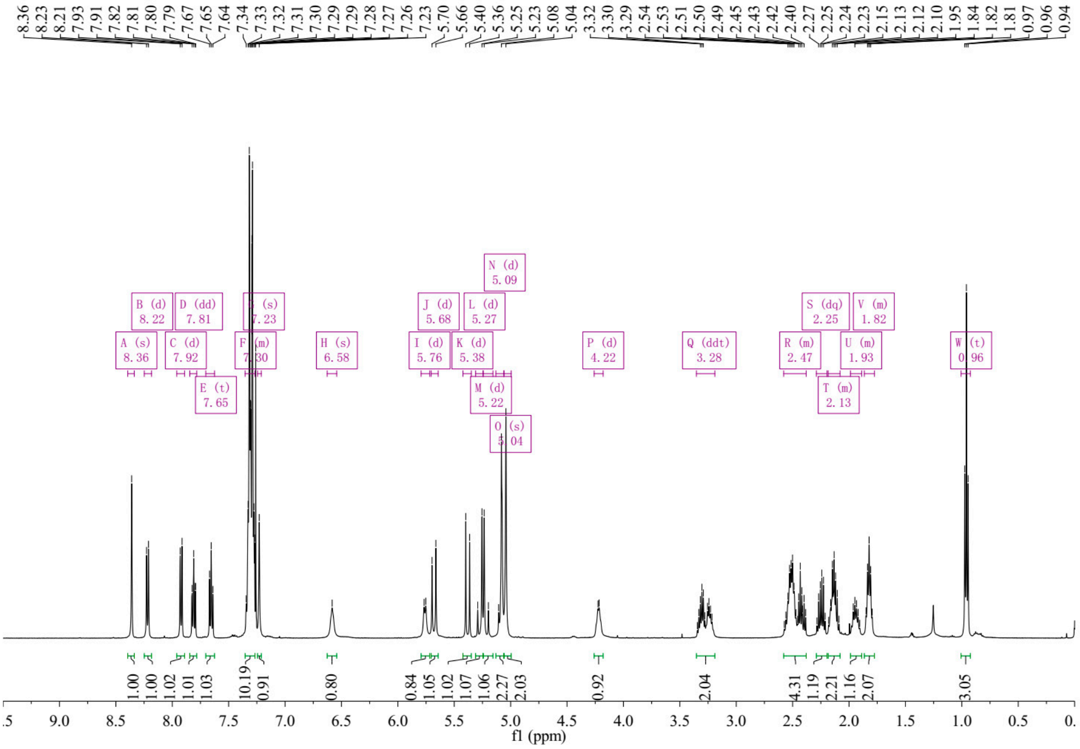

Figure S103: The ^{1}H-NMR of **CPT-C-GL4**.

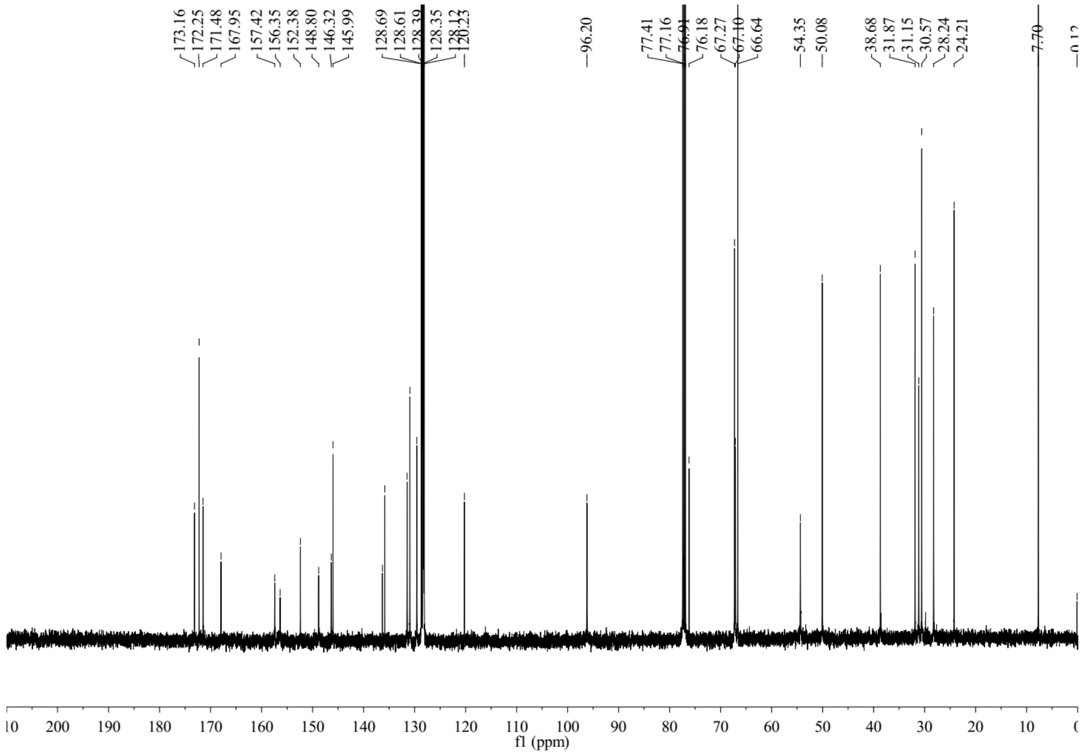

Figure S104: The ^{13}C-NMR of **CPT-C-GL4**.

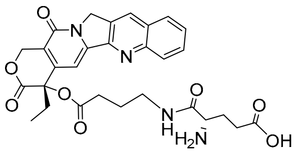

Figure S105: The structure of **CPT-D-GL4**.

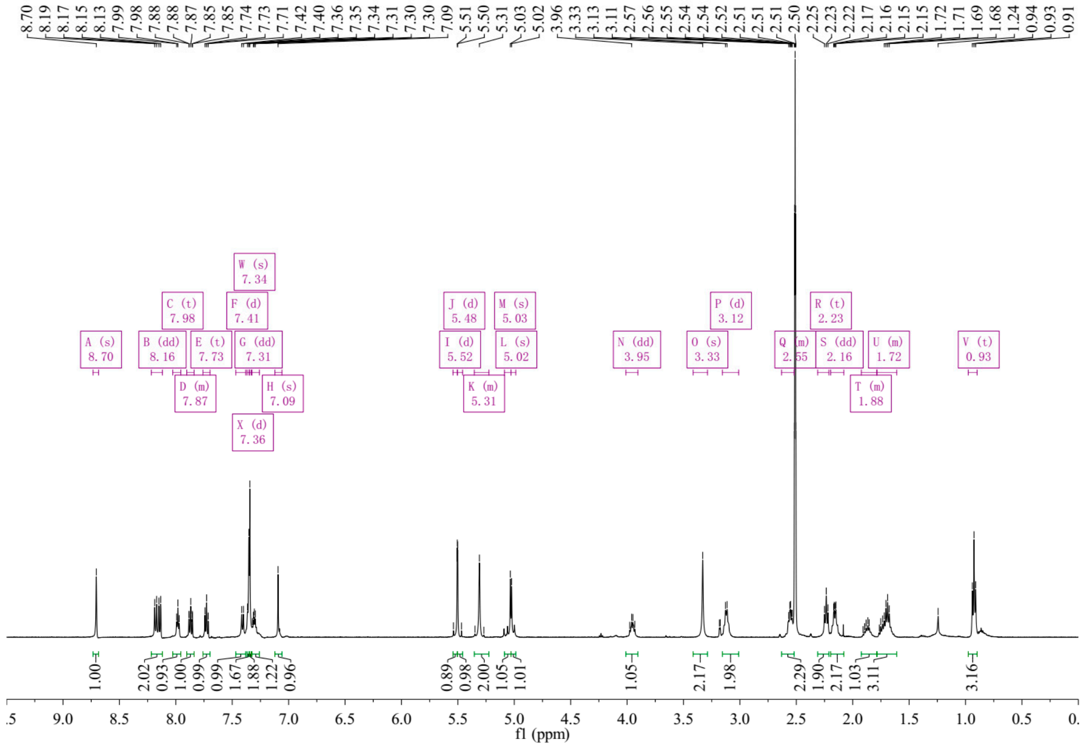

Figure S106: The ^{1}H-NMR of **CPT-D-GL₄**.

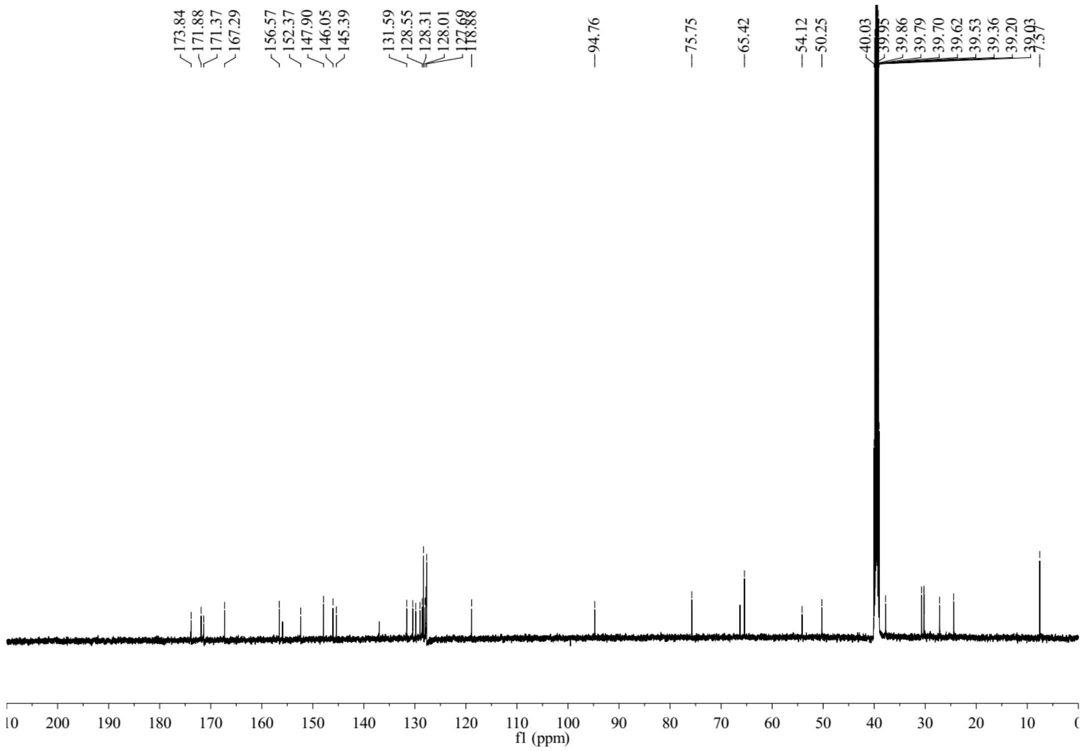

Figure S107: The ^{13}C-NMR of **CPT-D-GL₄**.

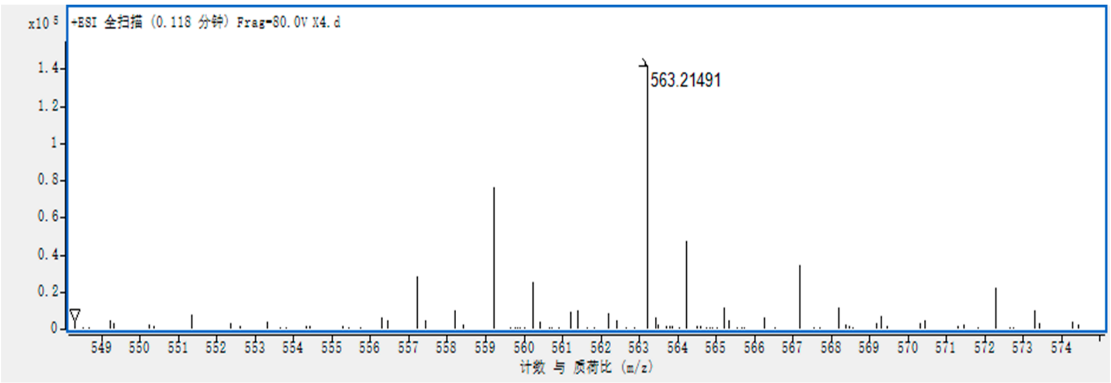

Figure S108: The HRMS of **CPT-D-GL4**.

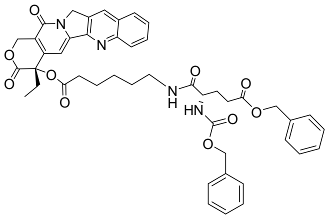

Figure S109: The structure of **CPT-C-GL6**.

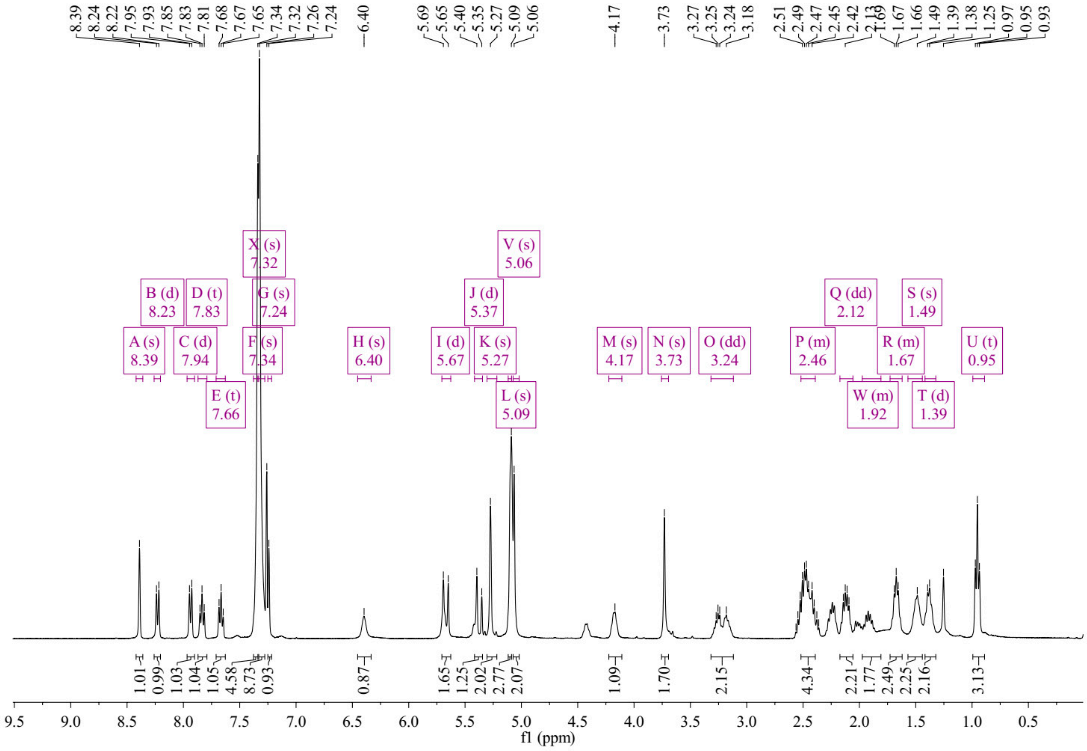

Figure S110: The ^{1}H-NMR of **CPT-C-GL6**.

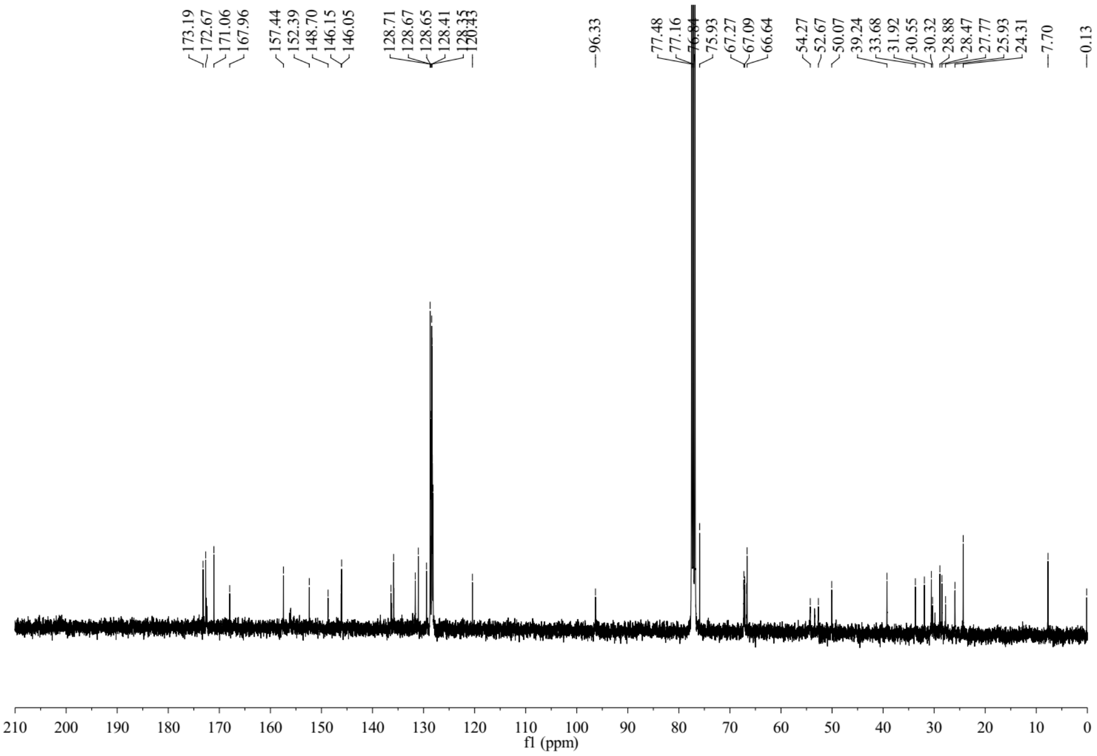

Figure S111: The ^{13}C-NMR of **CPT-C-GL$_6$**.

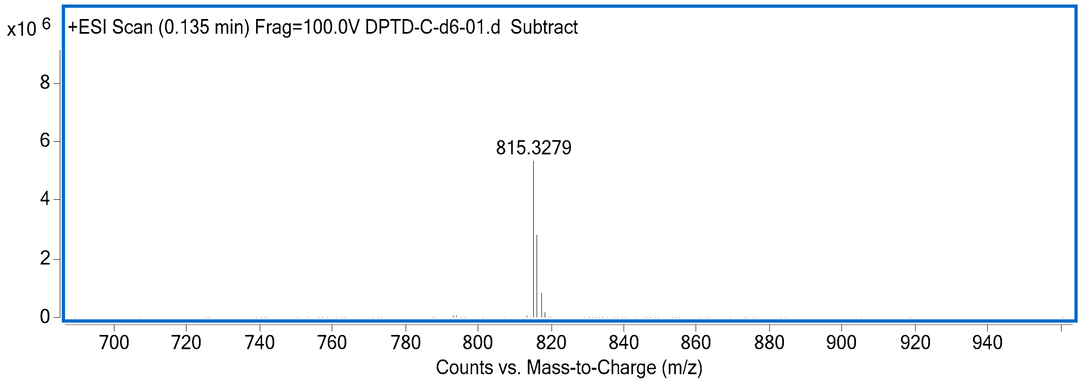

Figure S112: The HRMS of **CPT-C-GL$_6$**.

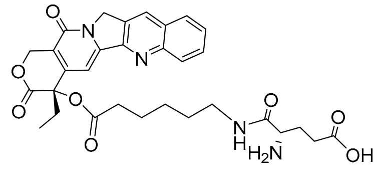

Figure S113: The structure of **CPT-D-GL$_6$**.

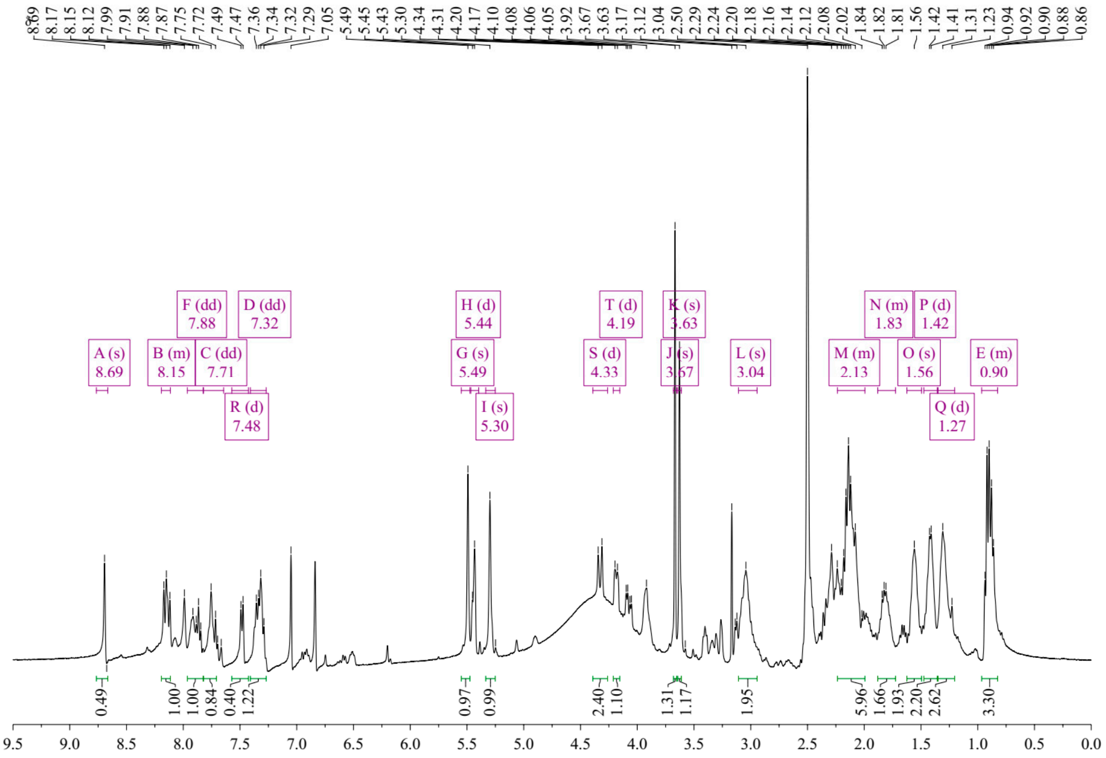

Figure S114: The ^{1}H-NMR of **CPT-D-GL$_6$**.

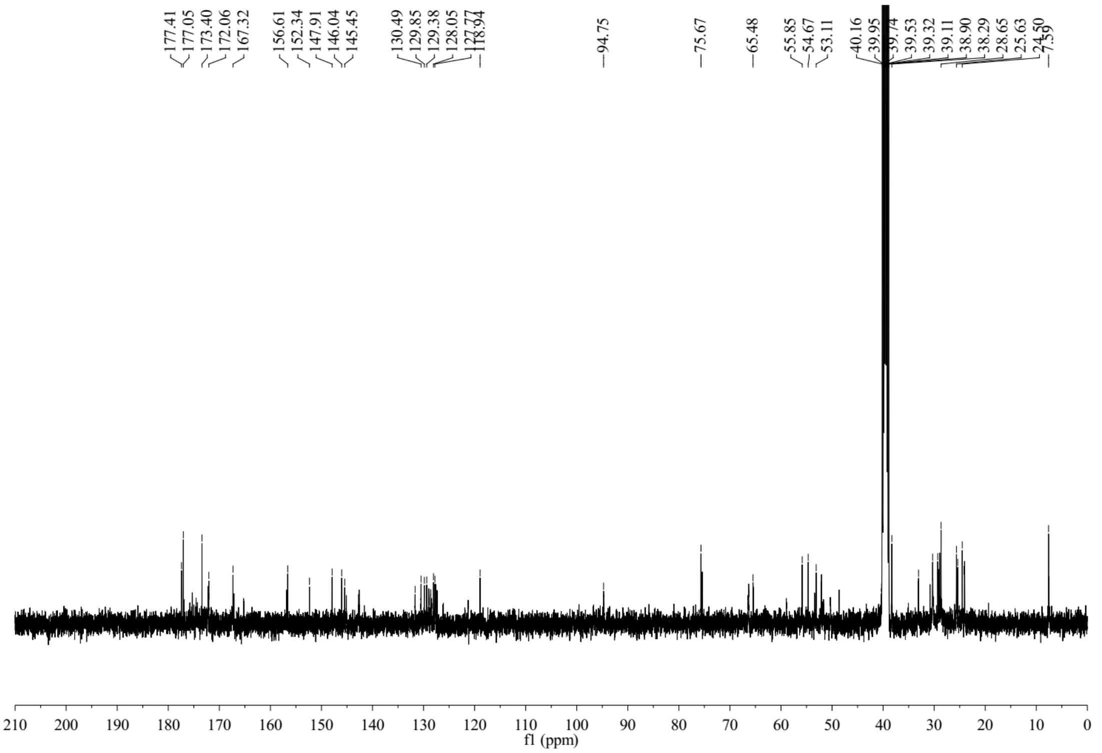

Figure S113: The ^{13}C-NMR of **CPT-D-GL$_6$**.

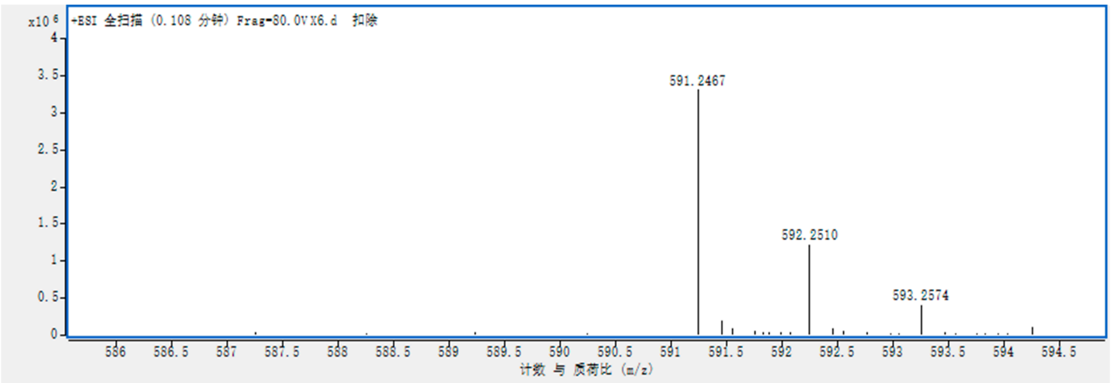

Figure S114: The HRMS of **CPT-D-GL₆**.

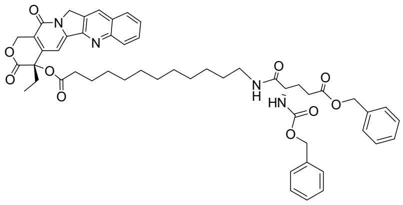

Figure S115: The structure of **CPT-C-GL₁₂**.

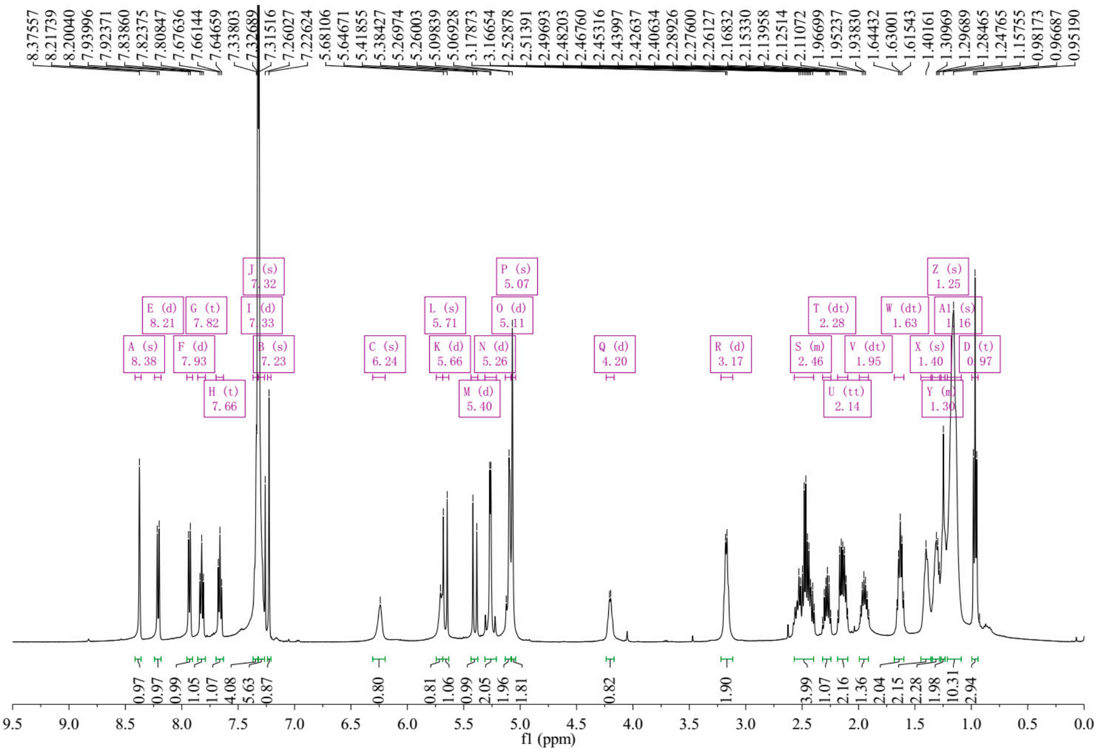

Figure S116: The ^{1}H-NMR of **CPT-C-GL12**.

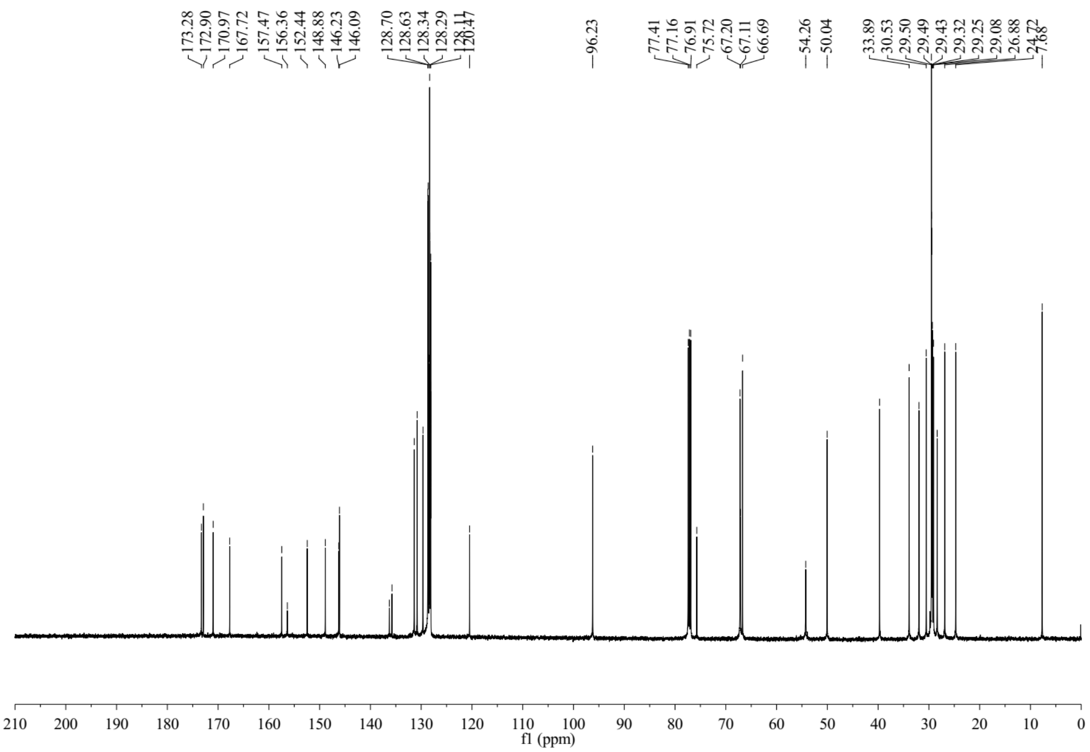

Figure S117: The ^{13}C-NMR of **CPT-C-GL12**.

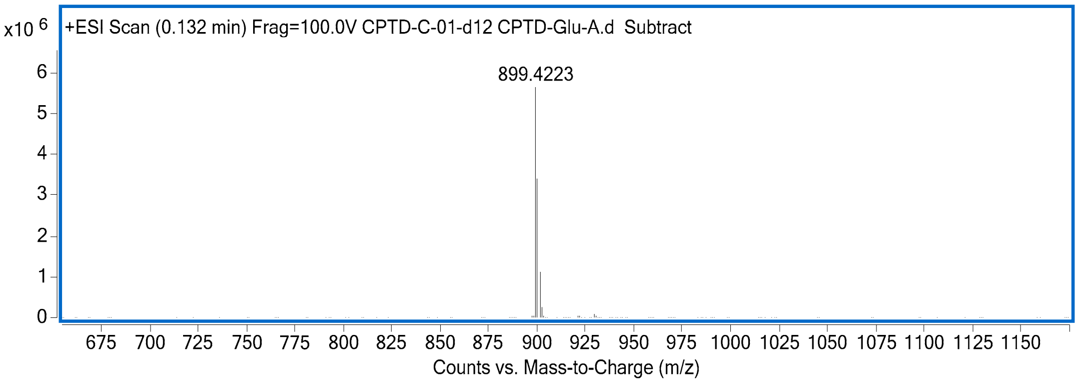

Figure S118: The HRMS of **CPT-C-GL12**.

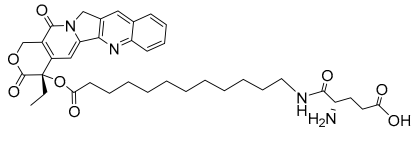

Figure S119: The structure of **CPT-D-GL12**.

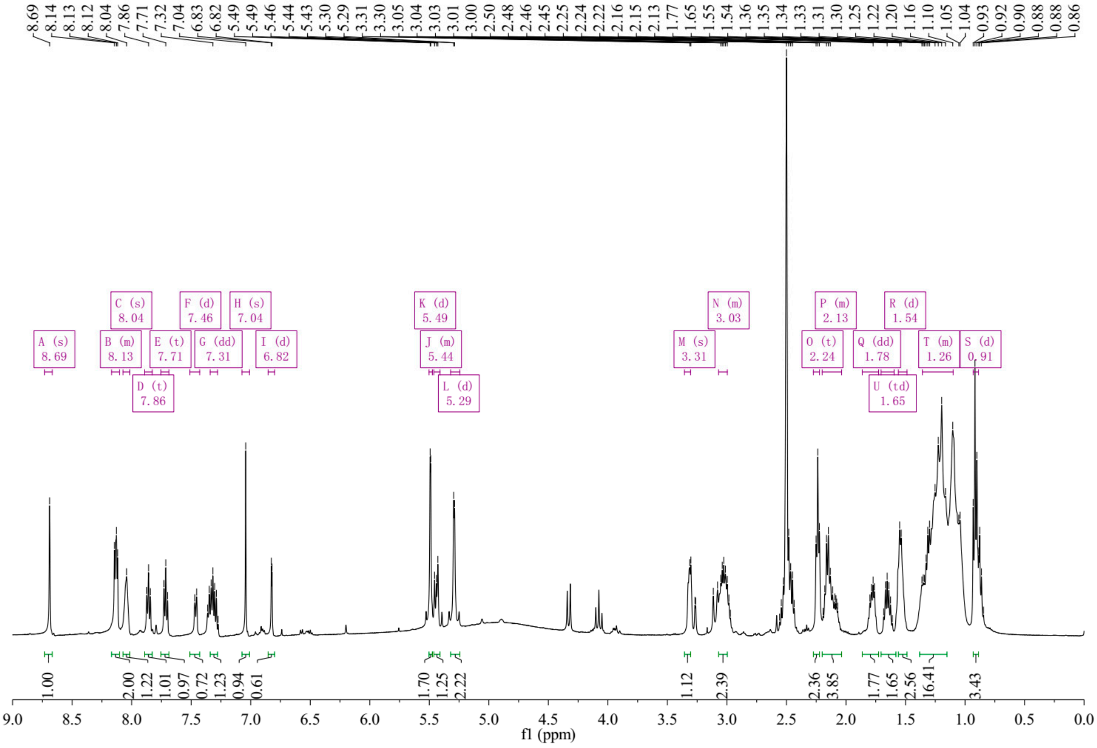

Figure S120: The ^{1}H-NMR of **CPT-D-GL$_{12}$**.

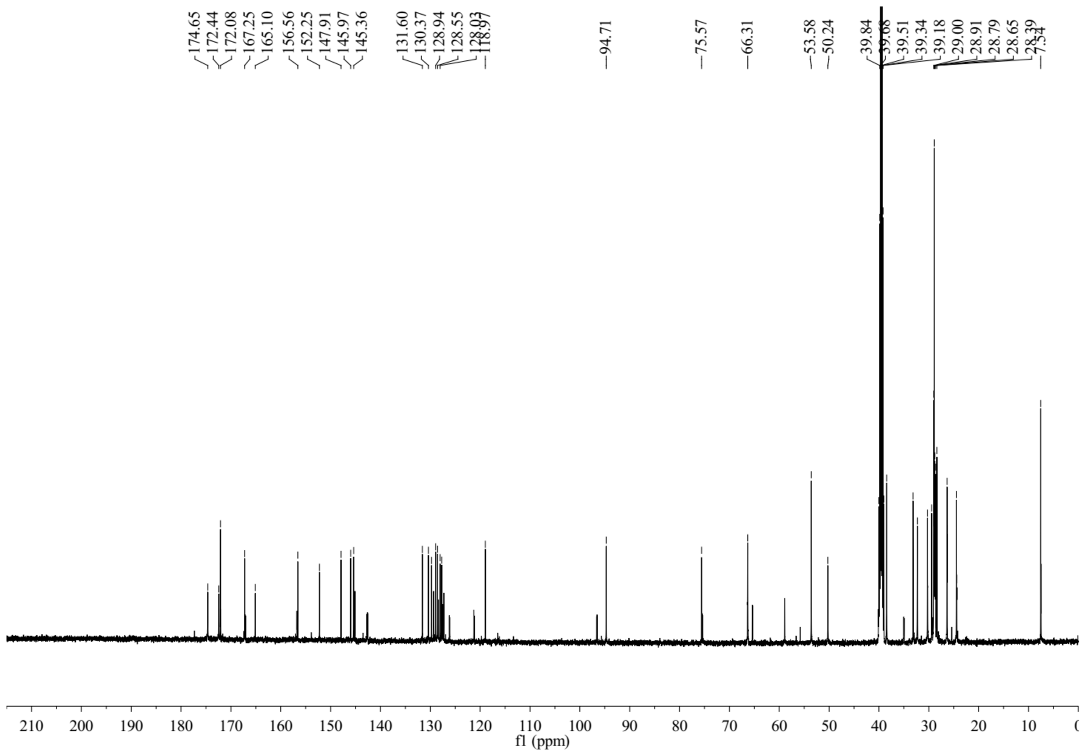

Figure S121: The ^{13}C-NMR of **CPT-D-GL$_{12}$**.

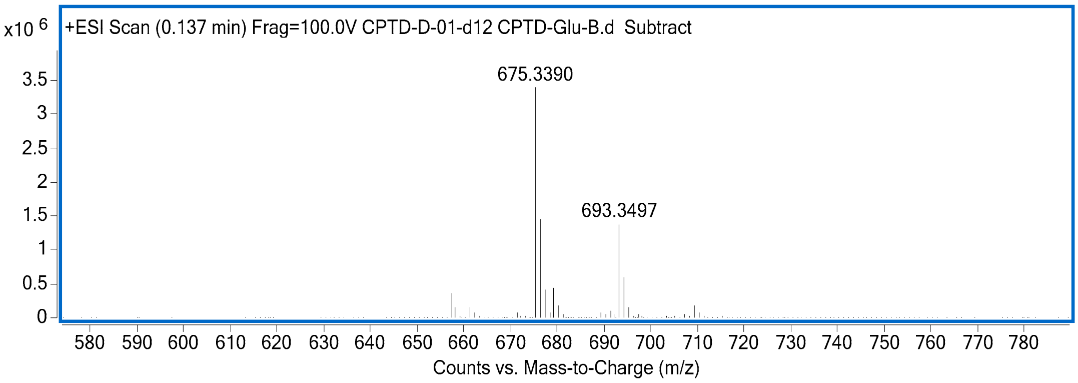

Figure S122: The HRMS of **CPT-D-GL12**.
